# Conjugated Oligo-Aromatic Compounds Bearing a 3,4,5-Trimethoxy Moiety: Investigation of Their Antioxidant Activity Correlated with a DFT Study

**DOI:** 10.3390/molecules21020224

**Published:** 2016-02-17

**Authors:** Huda. S. Kareem, Nurdiana Nordin, Thorsten Heidelberg, Azlina Abdul-Aziz, Azhar Ariffin

**Affiliations:** 1 Department of Chemistry, Faculty of Science, University of Malaya, Kuala Lumpur 50603, Malaysia; hudasalah55@yahoo.com (H.S.K.); csi_ndiana@um.edu.my (N.N.); heidelberg@um.edu.my (T.H.); 2 General Directorate of Curricula, Ministry of Education, Baghdad 3310, Iraq; 3 Department of Molecular Medicine, Faculty of Medicine, University of Malaya, Kuala Lumpur 50603, Malaysia; azlina_aziz@um.edu.my

**Keywords:** thiosemicarbazide, triazole, oxadiazole, antioxidant, hydrogen atom transfer, single electron transfer

## Abstract

A series of heterocyclic compounds bearing the well-known free radical scavenging 3,4,5-trimethoxybenzyloxy group, was synthesized. The key compound 4-(3,4,5-trimethoxybenzyl-oxy)benzohydrazide was converted into thiosemicarbazide derivatives, which were subsequently cyclized with NaOH to provide 1,2,4-triazole derivatives. Alternative treatment of the acid hydrazide with carbon disulfide in the presence of KOH led to the corresponding 1,3,4-oxadiazole and various alkylated derivatives. The newly synthesized compounds were purified and the structures of the products were elucidated and confirmed on the basis of their analytical and spectral data. Their antioxidant activities were evaluated using 2,2-diphenyl-1-picrylhydrazyl (DPPH^•^) and Ferric Reducing Antioxidant Power (FRAP) assays. The thiosemicarbazide derivatives were highly active in both antioxidant assays with the lowest IC_50_ value for DPPH radical scavenging. Theoretical calculations based on density functional theory (DFT) were performed to understand the relative importance of NH, SH and CH hydrogens on the radical scavenging activities of these compounds.

## 1. Introduction

Reactive oxygen species (ROS) is a collective term for different reactive molecules and free radicals derived from molecular oxygen, which are continuously produced as byproducts of mitochondrial electron transport of cellular respiration in the body. The most common ROS include superoxide anion (O_2_^−^), hydrogen peroxide (H_2_O_2_), hydroxyl radical (HO^•^) and singlet oxygen (^1^O_2_) [1]. These species are unstable and easily react with other molecules to achieve stability. The detrimental effects of free radicals occur when they are present in large quantities, *i.e.*, if there is an imbalance between the production and removal/inactivation of ROS. The pro-oxidative state when ROS level exceeds the capability of defense mechanisms is often referred to as “oxidative stress”. This poses a threat to cells, causing damage to DNA, lipids and proteins, potentially leading to disease conditions such as cell ageing, cardiovascular diseases and cancerous tumor growth [2,3]. Antioxidants provide protection against oxidative damage by scavenging free radicals and reducing ROS, hence inhibiting the oxidation of lipids or other molecules in biological systems [4]. For this reason, synthetic compounds are widely studied for their antioxidant activities using different methodologies [5,6].

Thiosemicarbazides are interesting derivatives, being versatile intermediates for the synthesis of important heterocycles, such as triazoles, thiadiazoles, oxadiazoles and thiazolidinones [7]. They form a large group of thiourea derivatives. The conjugated N-N-S tridentate ligand system of thiosemicarbazide (NH_2_-CS-NH-NH_2_) is responsible for various biological activities, which exhibit strong inhibitory effects on lipoprotein (LP) levels and substantially higher scavenging effects on the DPPH radicals than their cyclic analogues, 1,2,4-triazole-3(4*H*)-thiones [8]. The ability of heterocyclic compounds possessing 1,3,4-oxadiazole to undergo various chemical reactions make them privileged building blocks for pharmaceutically active compounds [9].

The presence of methoxy groups on aromatic systems is important in microtubule-binding and cytotoxic behavior for cancer chemotherapy [10,11]. An example is 10-(3,4,5-trimethoxy)benzyl-9(10*H*)-acridinone, which has shown high activity in *in vitro* antitumour tests against CCRF-CEM leukemia cells [12]. Structure activity relationship (SAR) studies on combretastatin A-4, which is an anti-tumour drug from the combretastatin group [13], have shown that the 3,4,5-trimethoxyphenyl groups are important for its cytotoxic activity [14,15].

Another example of compounds with a 3,4,5-trimethoxy benzyl group is trimethoprim (TMP), one of the most frequently detected antibiotics in the environment, that has been detected in municipal wastewater effluent at concentrations of several hundred nanograms per liter [16,17,18,19]. The antifolate TMP is used in the treatment of *Pneumocystis carinii* (pc) and *Toxoplasma gondii* (tg), the leading causes of mortality and morbidity in patients with AIDS [20]. Methoxy groups on aromatic also have been extensively investigated for their ferrous ion-chelating activities [10], which may enhance the stability of the radical due to the electron conjugation effect.

**Figure 1:**
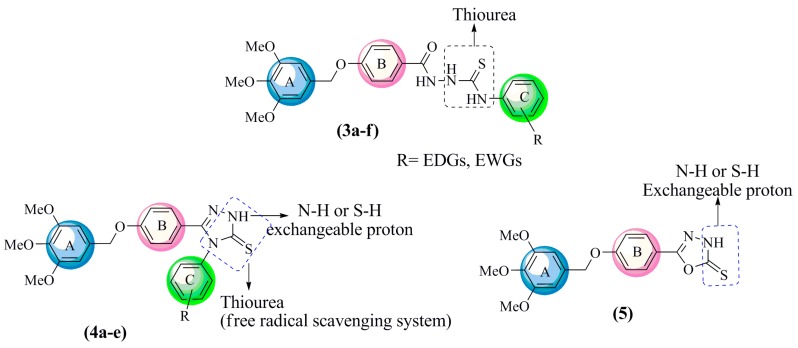
SAR analysis of 1-arylthiosemicarbazides **3a**–**f**, 1,2,4-triazoles **4a**–**e** and 1,3,4-oxadiazole **5**.

The aim of this study was to synthesize a new series of hybrid molecules containing oxadiazoles, triazoles and their open-chain analogs thiosemicarbazides, and to determine their radical scavenging capacities. The new compounds were designed to have built-in multipotent antioxidants (MPAO) in one structure. This feature can be strongly correlated to C=O and C=S bonds, likewise, the presence of three amide NH groups in thiosemicarbazide, and the exchangeable proton in 1,2,4-triazole and 1,3,4-oxadiazole. The structures of these compounds are shown in Figure 1. The synthesized compounds have been characterized by IR, NMR and mass spectral analyses. The antioxidant activities were experimentally verified using DPPH and FRAP assays, and further rationalized based on computational studies using DMOL3 on DFT-1.

## 2. Results and Discussion

### 2.1. Synthesis

**Scheme 1:**
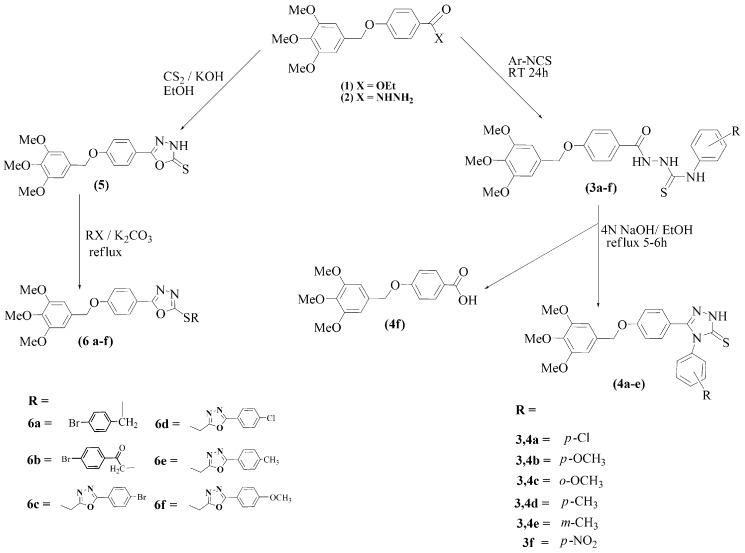
Synthesis of target compounds **3**, **4**, **5** and their derivatives.

**Scheme 2:**
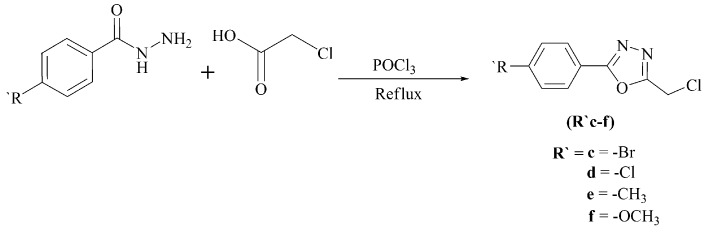
Synthesis of 2-chloromethyl-5-aryl-1,3,4-oxadiazoles (**R′c**–**f**).

The syntheses are outlined in Scheme 1 and Scheme 2. All structures were confirmed by IR, NMR and mass spectral analyses. The synthesis of starting materials **1** and **2** followed a previous publication [21]. The thiosemicarbazides **3a**–**f** were obtained in high yields by treatment of acid hydrazide **2** with aryl isothiocyanates in absolute ethanol [22]. Subsequent reflex with 4 M sodium hydroxide in ethanol resulted in the formation of 1,2,4-triazoles **4a**–**e** [23]. However, **3f** led to saponification of the hydrazide. Refluxing hydrazide **2** with carbon disulfide in ethanolic KOH [24], provided the 1,3,4-oxadiazole **5**, as shown in Scheme 1. It was alkylated with various alkyl halides, leading to compounds **6a**–**f**.

The synthesis of non-commercial halides is shown in Scheme 2. Refluxing aryl acid hydrazides with chloroacetic acid in the presence of phosphoryl chloride provided 2-chloromethyl-5-aryl-1,3,4-oxadiazoles (**R′c**–**f**) [25].

### 2.2. In Vitro Free Radical Scavenging Activities

In general, the synthesized compounds **3**–**6** were tested for their antioxidant activities, *in vitro* using two widely used antioxidant assays, 2,2-diphenyl-1-picrylhydrazyl (DPPH^•^) and Ferric Reducing Antioxidant Power (FRAP) assays [26,27]. These assays deactivate radicals by two major mechanisms: the hydrogen atom transfer (HAT) and single electron transfer (SET) mechanism. The DPPH assay is based on either a HAT or a SET mechanism, while FRAP assay is based on a SET mechanism. Owing to the different reaction mechanisms, DPPH and FRAP determined antioxidant activities differ in value. The HAT mechanism is solvent and molecular structure independent and usually quite rapid, typically completed in seconds to minutes [28]. HAT measures the classical ability of an antioxidant to quench free radicals by hydrogen donation. SET measures the ability of a potential antioxidant to transfer one electron to reduce the radicals [29]. SET requires the initial formation of a cation radical from ArXH by transfer of an electron to the radical, followed by rapid deprotonation of ArXH^+•^, which compensates for the charge of the initial anion R¯ [30].

#### 2.2.1. DPPH Free Radical Scavenging Activities

The synthesized compounds **3**–**6** were tested against DPPH at different concentrations and were compared with ascorbic acid (AA) and butylated hydroxytoluene (BHT), as standard antioxidants. All results are presented as IC_50_ values and maximum radical scavenging in Table 1.

**Scheme 3:**
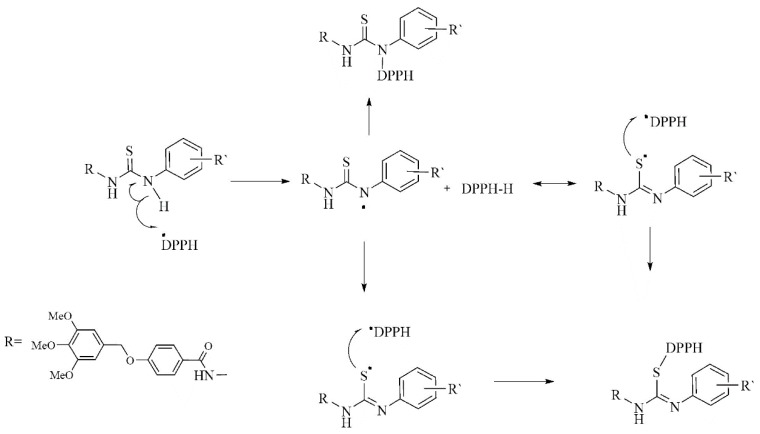
Proposed mechanism for the scavenging of DPPH**^•^** by thiosemicarbazide derivatives **3a**–**f**.

The higher free radical scavenging activities of thiosemicarbazide compounds **3a**–**f**, could be attributed to the presence of an NH group from the aromatic amines in the thiosemicarbazides, which can donate a hydrogen atom via a HAT mechanism leading to neutralization of the DPPH radical [31]. The resulting free radical was stabilized by delocalization of the odd electron into the aromatic ring [32]. The DPPH radical scavenging potential of thiosemicarbazide compounds **3a**–**f** can be explained according to the proposed mechanism in Scheme 3 [33]. The higher free radical scavenging activities of compounds **3a**–**f**, could be attributed to the presence of an NH group attached to the aromatic ring in thiosemicarbazides, which can donate a hydrogen atom HAT mechanism, leading to neutralization of the DPPH radical [31]. The scavenging mechanism showed that the arylthiourea in thiosemicarbazides is able to neutralize two DPPH radicals. Due to their steric hindrance a reaction of two DPPH molecules is not possible [34]. Support for the proposed mechanism is provided by a previous report on the antioxidant activities of aromatic amine derivatives [35], and five-membered heterocyclic amines [36]. The aromatic amines form an important class of antioxidants, similar to phenolic derivatives [37]. They can easily transfer their amine hydrogen to peroxyl radicals, making them good H-donors [38].

**Figure 2:**
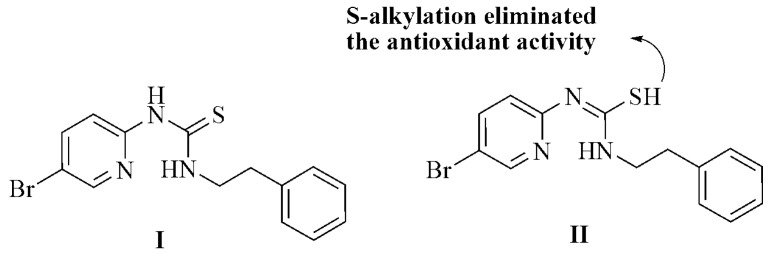
Thiol-thione tautomerism and S-alkylation in phenethyl-5-bromo-2-pyridyl thiourea.

Further evidence can be found in the antioxidant activity of phenethyl-5-bromo-2-pyridyl thiourea, which exists in both thiol and thione form. The thiol has been shown to exhibit antiradical activities and all phenethyl-5-bromo-2-pyridyl thiourea compounds exhibited antioxidant activities (Figure 2I). The thiol-thione tautomerism has been evaluated based on implications from the antioxidant activities.

The results using S-alkylated derivatives indicated that an unalkylated thiourea group is critical for antioxidant activity and that S-alkylation virtually eliminates this activity (Figure 2II). This result suggests that the thiol group (II) is responsible for the antioxidant activities due to its favorable electron-donating characteristics [39].

**Scheme 4:**
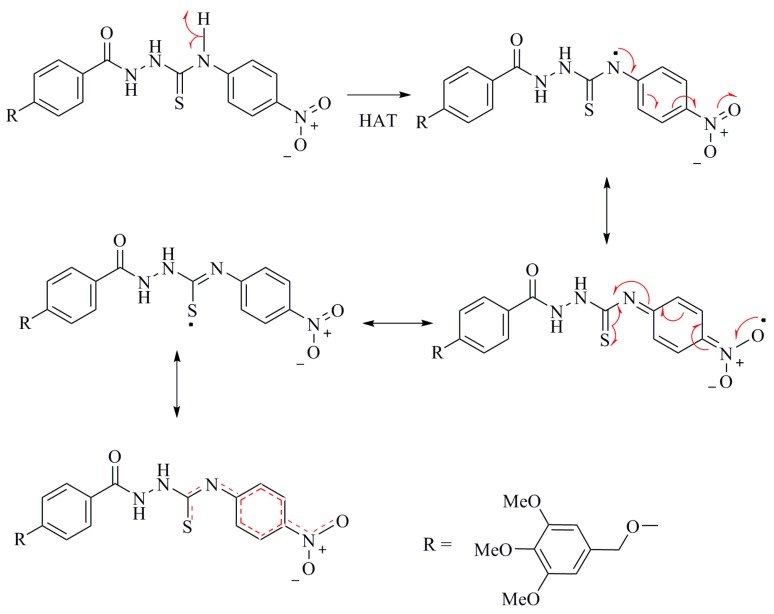
Delocalization of the electron in the radical of compound **3f**.

As can be seen from Table 1, the thiosemicarbazides **3a**–**f**, exhibited stronger inhibitory effects in both antioxidant assays than triazoles **4a**–**e** or oxadiazole **5** and its derivatives **6a**–**f**. All thiosemicarbazides achieved >50% inhibition of the DPPH radical. The order of the activity is as follows: **3f** > **3d** > **3b** > **3a** > **3c** > **3e**. These activities are comparable to ascorbic acid (AA) and butylated hydroxytoluene (BHT) standards [40,41]. Among these, compound **3f** with IC_50_ 29 ± 2 and maximum inhibition of 89% ± 1.9% demonstrated the highest activity. The presence of a strong electron-withdrawing group, such as NO_2_ on the phenyl ring, had a great impact on the activity. The electron withdrawing effect polarizes the π-electrons of the phenyl ring, which enhances the acidity of the conjugated NH group, exceeding the acidity of the other NH groups in the compound. Therefore, the thioamide hydrogen can be abstracted to form a structure stabilized by delocalization of electrons throughout the molecule, as shown in Scheme 4. Compounds **3f**, **3d**, **3b** and **3a**, with substitution on the *para*-position of the phenyl ring, regardless of whether it is electron donating or withdrawing, are likely to be more active than the *ortho*- and *meta*-substituted compounds **3c** and **3e**, respectively. A pattern of increasing activity follows the order, *para* > *ortho* > *meta* [42]. The *p*-methoxy substituents exhibit higher activity than the *ortho*-analog. This is in agreement with other reports describing the decrease in radical scavenging activities of phenol (or aniline), upon substitution in the *ortho*-position.

**Figure 3:**
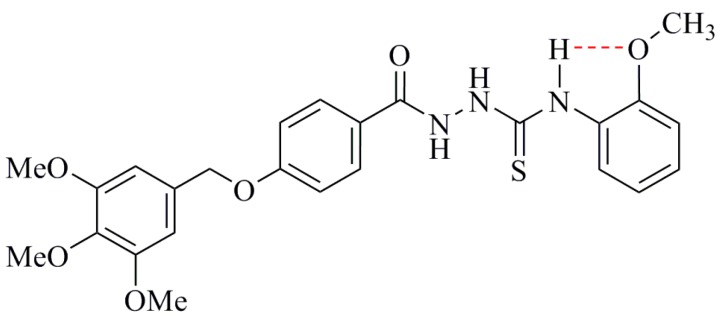
Formation of intramolecular hydrogen bond in compounds **3c**.

The reason for the decreased radical scavenging activity of *ortho*-alkoxy compounds is an intramolecular hydrogen bonding involving the NH as donor and free electron pairs on the oxygen as acceptor [43]. This hydrogen bond strengthens the binding of the thioamidic NH-hydrogen, requiring more energy for its removal and, hence, reduces the DPPH scavenging activity [44]. This H-bond increases the energy needed to abstract the hydrogen atom from a phenolic hydroxyl group [45], a similar effect is to be expected for the NH group of compound **3c** based on the hydrogen bonding shown in Figure 3.

On the other hand, the 1,2,4-triazole compounds **4a**–**e**, as well as the hydrolyzed compound **4f** exhibited only moderate to low DPPH radical scavenging activities, comparable with BHT as listed in Table 1. The activities decrease in the order, **4a** > **4d** > **4b** > **4f** > **4c** > **4e**. Compound **4a**, which has a p-chlorophenyl group with an IC_50_ = 154 ± 11 and 46% ± 1.6% inhibition, has better activity than the other compounds [46]. The compounds with electron donating substituents, such as *p*-CH_3_ (**4d**) and *p*-OCH_3_ (**4b**) exhibited higher antioxidant activities than the remaining compounds **4f**, **4c** and **4e** [47]. The stereoelectronic effects of the p-methoxy group stabilized the aryloxyl or arylaminyl radical through a p-type lone-pair orbital on the *para* heteroatom as reported in the literature [47,48].

##### DFT Study of the DPPH Radical Scavenging Activities of Thiosemicarbazide

**Figure 4:**
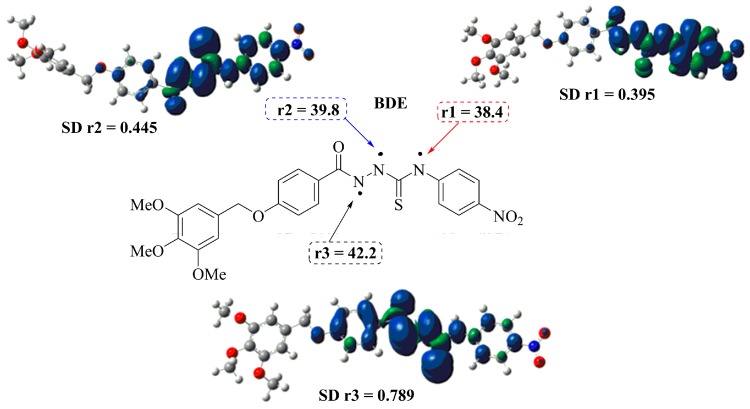
Density distributions of the SOMOs, BDE and SD calculated on the **r1-N**, **r2-N** and **r3-N** atoms in their radicals of compound **3f**.

In order to rationalize the experimental observations, density functional theory (DFT) calculations have been carried out to estimate the actual energetics for different reactions, and evaluate the stability of radicals generated by H-abstraction from NH groups in the synthesized compounds. Bond dissociation enthalpies (BDEs) have also been calculated at the B3LYP theory level for the respective H atom elimination paths. The calculations applied DMSO solvent-phase conditions. The two main factors determining the free radical scavenging activity of antioxidants are the strength of the bond for the hydrogen atom and the electron donating ability of the antioxidants. Lower BDEs are associated with higher antioxidant activity [38]. Table 2 lists the calculated value in DMSO solvent-phase BDEs for the studied compound **3f**. Comparing the BDE values suggests abstraction of H in **r1**, *i.e.*, at the thioamide group. For this radical, the *p*-type orbital of the nitrogen atom at the nitro group delocalizes the unpaired electron, stabilizing the radical and lowering the BDE. As expected, the decrease in the electron density at N1 for **3f-r1** is reflected in the low spin density. The density distributions of the semi-occupied orbital (SOMOs), spin densities and BDE of the respective radical species of compound **3f** in different positions were determined separately. The results for these separate calculations are summarized in Figure 4.

The BDE in **3f-r2** increased due to the involvement of the hydrogen in an intramolecular hydrogen bond with the hydrazide carbonyl, which requires more energy to be abstracted. The same applies for **3f-r3** based where a thiocarbonyl is involved. In fact, the position of **r3** requires even more energy for the abstraction of the H.

##### DPPH Free Radical Scavenging Activities for Oxadiazole

**Scheme 5:**
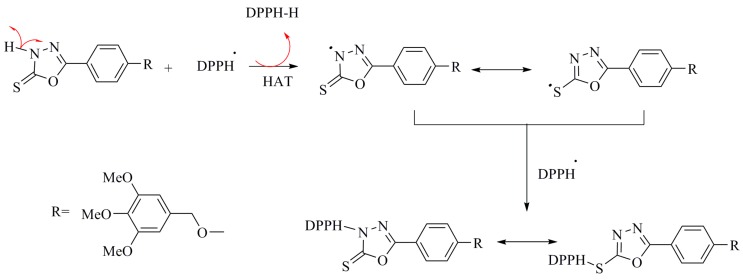
Proposed HAT mechanism for scavenging of DPPH^•^ by 1,3,4-oxadiazole.

Compound **5** exhibited strong scavenging effect on the DPPH radical, with an IC_50_ value of 45 ± 2 µg/mL and 73% ± 1.6% maximal inhibition. This inhibition is greater than that of BHT. The strong radical scavenging activity of compound **5** is attributed to the acidic proton in the oxadiazole moiety based on a HAT mechanism. Conjugation of the primary radical, resulting from the removal of an H-atom from the heterocycle, stabilizes the radical, thus lowering its energy. A proposed mechanism for the scavenging of the DPPH free radical by compound **5** is presented in Scheme 5.

Previously reported work has described the proposed mechanism [39], in which both thiol and thione tautomeric forms are responsible for the antioxidant activity. The interaction of the oxadiazole derivatives with the DPPH free radical indicates their free radical scavenging ability. Compounds **6a**–**f** did not reach 50% inhibition of the DPPH radicals within the concentration range investigated in this study. In contrast, S-alkylation of compound **5** eliminated its activity, which indicates the critical role of the exchanging proton for the antioxidant behavior of the compound, thus suggesting the involvement of this bond in a HAT mechanism. Previous studies have already stated the importance of non-S-alkylated thioamide [49], especially the antioxidant activities for the thiourea group [50]. It is assumed that the low remaining radical scavenging activities following S-alkylation originates from a SET mechanism, which involves electron transfer followed by proton abstraction.

The DPPH free radical scavenging activity of compounds **6a**–**f** can arise either from a methylene (CH_2_) group attached to the 3,4,5-trimethoxyphenyl ring or from the CH_2_ near the oxadiazole moiety. A reactive free radical can undergo electron transfer or abstract an H-atom from either of these two sites [51,52]. The low activity of S-alkylated derivatives of **5** indicate that hydrogen abstraction from the -CH_2_ group in these compounds by DPPH is not a favored process. The order of DPPH radical scavenging activities based on maximal inhibition, of the oxadiazole derivatives **6a**–**f** were **6f** > **6e** > **6d** > **6b** > **6c** > **6a**. Compound **6b** was more active than compound **6a**, though both bore the *p*-bromophenyl. This is due to the presence of the carbonyl substituent that enhanced the activity of compound **6b**. Despite increasing the aromaticity of the substituent in the synthesized compounds **6c**–**f**, the majority of these compounds showed low interaction with the DPPH radical.

However, the substitution of electron donating or electron withdrawing (EDG or EWG, respectively) on the aromatic ring effected DPPH^•^ scavenging activities for the synthesized compounds. In compounds **6e** and **6f** the presence of EDGs, methyl and methoxy groups on the phenyl ring at the *para*-position might favor the activity, whereas the remaining compounds with EWGs showed less antiradical activities. The results from the above experiments thus confirm that the antioxidant activities are more pronounced in oxadiazole compound **5** having the NH group compared to the ones with S-alkylation. This may be due to the presence of the NH group, which can easily donate hydrogen compared to a CH_2_ group.

##### DFT Study of the DPPH Radical Scavenging Activities of Oxadiazole

In the absence of either OH or NH groups, H-abstraction from the two CH_2_ groups in the oxadiazole derivatives has been compared by density functional theory (DFT) calculations. Computational studies were carried out on compounds **6a** and **6b** in order to compare the energy between the abstraction of an H atom from the CH_2_ groups at the two different sites (Table 3). The BDE for each CH radical position is achieved by taking the difference between the total energy of the parent molecules and the total energy of the free radical species.

Table 3 shows the figure of radical molecules in which the H atom has been abstracted at two positions, **r1** and **r2**. The BDE of **6a-r2** is slightly lower than BDE for **6a-r1** showing that the abstraction of the H atom from the **r2** position gave a more stable radical than **r1**. This is probably due to the phenyl ring attached to the bromine atom, creating an electron withdrawing group and thus, increasing the radical stability at the **r2** position.

For compound **6a** the bond lengths for H-CH (**r1**) and H-CH (**r2**) were calculated and the values are 1.095 Å and 1.099 Å. The total energy for **6a-r2** was higher than **6a-r1**, which showed that the hydrogen abstraction from **6a-r2** to form radical is more favorable compared to **6a**-**r1**. As shown in Table 3, the dissociation processes are calculated to be barrier-less and the energy required to break the H-CH (**r1**) and H-CH (**r2**) bonds are predicted with values 290.0 kcal/mol and 278.2 kcal/mol, confirming that the H-CH (**r2**) was weaker. However, bond lengths for H-CH (**r1**) and H-CH (**r2**) for compound **6b** are 1.096 Å and 1.082 Å, which means the bond distance of H-CH (**r2**) is shorter and the process of H abstraction is possibly easier in H-CH (**r1**) than H-CH (**r2**). The energy of the radicals suggests that in compound **6b**, the H-CH (**r1**) is more stable than the H-CH (**r2**), thus the abstraction of the radical in **r1** is preferred over **r2**.

The BDE value of **6b-r1** is lower than **6b-r2**, confirming that the H-CH (**r1**) bond is weaker and the spin density of the H-CH (**r1**) and H-CH (**r2**) are 0.921 and 0.979 respectively, showed that the abstraction of H from H-CH (**r1**) is possible compared to H-CH (**r2**) [38]. Furthermore, the highest occupied molecular orbital (HOMO) energy which characterizes the ability of electron-giving is appropriate to represent the free radical scavenging efficiency of the compounds because the process to inhibit auto-oxidation may include the electron transfer besides the abstraction of the H-atom [53]. On the other hand, the atomic sites characterized by high density of the HOMO distribution are very sensitive to the attack of free radicals and other reactive agents. The higher energy HOMO orbital is delocalized, the more numerous are the electron sites, and more redox reaction will occur [54].

**Figure 5:**
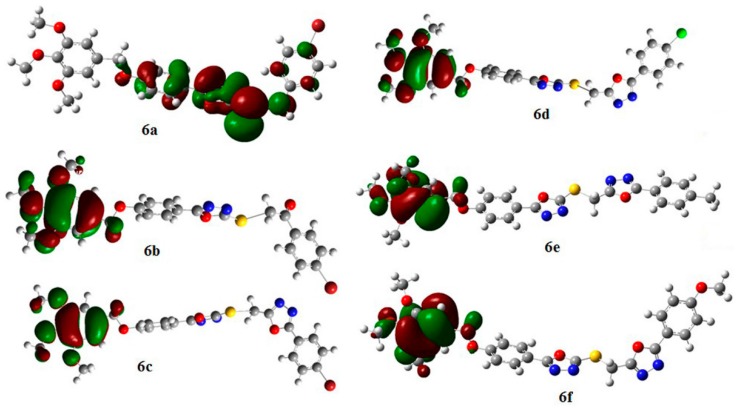
HOMO distribution for compounds **6a**–**f**.

The HOMO distribution for compounds **6b**–**f** (Figure 5) gave a picture that supports the explanation of the hydrogen abstraction above. The delocalization of electron distribution occurred surrounding the **r1** position, the trimethoxybenzyl site, which suggests that the electron distribution is quite stable in this region, and the favorable mechanism for these compounds may be the SET mechanism.

#### 2.2.2. Ferric Reducing Antioxidant Power (FRAP) Activities

The FRAP assay measures the ability of antioxidants to reduce the ferric ion Fe^3+^ [55,56], which may indirectly reflect the antioxidant capacity. The mechanism of FRAP assay is based totally on SET. Electron-donating ability is determined by the one-electron oxidation potential of the parent antioxidants, expressed by definition as the reduction potential of the corresponding radicals. As seen in Table 1, the FRAP values show a similar trend with the DPPH radical scavenging results indicating higher activities for thiosemicarbazides **3a**–**e** and oxadiazole **5**, and much lower activity for triazoles **4a**–**e** and oxadiazoles **6a**–**f**. However, compound **3f** which showed high DPPH radical scavenging activity, had low activity in the FRAP assay. This may be an indication of structural dependence of the antioxidant reaction in the DPPH assay. The activity order for the thiosemicarbazides based on the substituents in compounds **3e** > **3b** > **3d** > **3c** > **3a** > **3f** reflects the role of electron donating and withdrawing substituents to increase and decrease antioxidant capacity in this assay.

Compounds **3e** and **3b** bearing *m*-methyl and *p*-methoxy groups were highly active, with FRAP values of 2519 ± 103 and 2476 ± 64 µM, respectively, compared to ascorbic acid and BHT. Compounds **3d** and **3c** bearing *p*-methyl and *o*-methoxy groups also showed higher activity than BHT but less than ascorbic acid (AA), with values 1905 ± 42 and 1566 ± 26 µM respectively. Compounds **3a** and **3f** with electron withdrawing substituents showed much lower activities. The results clearly show that the higher activities for thiosemicarbazides while their conversion to triazole thiols **4a**–**e** reduced the activity, which might be attributed to the disappearance of the H atom on the nitrogen of thiosemicarbazide structure and and to fewer resonance structures. Oxadiazoles **6a**–**f** showed rather limited activities in FRAP, whereas compound **5** exhibited higher activity than BHT with a value of 1688 ± 12. Thus, from this feature of the synthesized compounds virtually all of the thiosemicarbazide derivatives are likely to exhibit favorable antioxidant properties.

## 3. Materials and Methods

### 3.1. General Information

Commercial chemicals and solvents were used without purification. NMR spectra were measured on a Bruker 400 MHz FT-NMR spectrometer (Bruker, Falladen, Switzerland) using DMSO-*d*_6_ as solvent. HRESI mass spectra were recorded at the Department of Chemistry at the University of Singapore on a MAT 95 x1-T spectrometer at 70 eV (Agilent Technologies, Santa Clara, CA, USA). Melting points were determined using a Mel-Temp II melting point apparatus (Laboratory Devices Inc., Holliston, MA, USA) and are uncorrected. IR spectra were recorded on a RX1 FT-IR spectrometer (Perkin-Elmer, Waltham, MA, USA). The absorbance of the reaction mixtures in the DPPH and FRAP assays were determined by UV spectroscopy using a Power Wave X340, BioTek Inc, Instrument (Winooski, VT, USA). The detailed spectrums and analyze are available in Supplementary Materials.

### 3.2. Synthesis

#### 3.2.1. General Procedure for the Synthesis of *N*-(4-aryl)-2-(4-(3,4,5-trimethoxybenzyloxy)Benzoyl)hydrazine Carbothioamides (**3a**–**f**)

To a stirred solution of compound **2** (0.40 g, 1.2 mmol), in absolute ethanol (15 mL), aryl isothiocyanate (1.2 mmol) was added. The reaction mixture was heated to 50 °C for 1 h, then stirred for 24 h at room temperature. The precipitate was collected by filtration, washed with cold absolute ethanol, dried under vacuum and purified by recrystallization from an appropriate solvent.

*N-(4-Chlorophenyl)-2-(4-(3,4,5-trimethoxybenzyloxy)benzoyl)hydrazinecarbothioamide* (**3a**). White solid (Ethanol, 0.53 g, 89%), m.p. 184–186 °C. IR: 3320 (NH), 3215 (NH), 3136 (NH), 1661 (C=O), 1591 (C=C), 1230 (C=S), 1122 (O-CH_3_). ^1^H-NMR δ, ppm: O-CH_3_ [3.66 (s, 3H), 3.78 (s, 6H)], 5.11 (s, 2H, O-CH_2_), 6.80 (s, 2H, ArH), 7.12 (d, *J* = 8.7 Hz, 2H, ArH), 7.37 (d, *J* = 8.7 Hz, 2H, ArH), 7.50 (d, 2H, ArH), 7.93 (d, *J* = 8.6 Hz, 2H, ArH), 9.75 (bs, 1H, NH), 9.81 (bs, 1H, NH), 10.40 (bs, 1H, NH). ^13^C-NMR δ, ppm: O-***C***H_3_ (56.4, 60.5), 70.1(O-***C***H_2_), Ar.C [105.8, 114.8, 125.3, 128.0, 128.3, 129.5, 130.3, 132.6, 137.6, 138.8, 153.4, 161.6 (C-O)], 166.0 (***C***=O), 181.6 (***C***=S). HREIMS, C_24_H_24_ClN_3_O_5_S, *m*/*z* = 524.1019, [M + Na]^+^. Calcd. 524.1012.

*N-(4-Methoxyphenyl)-2-(4-(3,4,5-trimethoxybenzyloxy)benzoyl)hydrazinecarbothioamide* (**3b**). White solid (Ethanol, 0.54 g, 91%), m.p. 160–162 °C. IR: 3321 (NH), 3222 (NH), 3182 (NH), 1665 (C=O), 1597 (C=C), 1234 (C=S), 1124 (O-CH_3_). ^1^H-NMR (400 MHz, DMSO-*d*_6_) δ, ppm: O-CH_3_ [3.65 (s, 3H), 3.74 (s, 3H), 3.77 (s, 6H)], 5.10 (s, 2H, O-CH_2_), Ar.H [6.79 (s, 2H), 6.89 (d, *J* = 8.7 Hz, 2H), 7.12 (d, *J* = 8.7 Hz, 2H), 7.28 (d, *J* = 8.7 Hz, 2H), 7.93 (d, *J* = 8.7 Hz, 2H)], 9.55 (bs, 1H, NH), 9.67 (bs, 1H, NH), 10.35 (s, 1H, NH). ^13^C-NMR (100 MHz, DMSO-*d*_6_) δ, ppm: O-***C***H_3_ (55.7, 56.4, 60.5), 70.1(O-***C***H_2_), Ar.C [105.8, 113.6, 114.7, 125.4, 128.1, 130.3, 132.5, 132.6, 137.5, 153.4, 157.2, 161.5], 166.0 (*C*=O). HREIMS, C_25_H_27_N_3_O_6_S, *m*/*z* = 520.1514, [M + Na]^+^. Calcd. 520.1507.

*N-(2-Methoxyphenyl)-2-(4-(3,4,5-trimethoxybenzyloxy)benzoyl)hydrazinecarbothioamide* (**3c**). White solid (Methanol: CHCl_3_, 0.55 g, 93%), m.p. 123–125 °C. IR: 3319 (NH), 3260 (NH), 3139 (NH), 1662 (C=O), 1595 (C=C), 1227 (C=S), 1125 (O-CH_3_). ^1^H-NMR (400 MHz, DMSO-*d*_6_) δ, ppm: O-CH_3_ [3.67 (s, 3H), 3.73 (bs, 3H), 3.78 (s, 6H)], 5.11 (s, 2H, O-CH_2_), Ar.H [6.80 (s, 2H), 6.93 (td, *J* = 8 Hz, 1H), 7.03 (d, *J* = 8 Hz, 1H), 7.14 (d, *J* = 8 Hz, 2H), 7.92 (d, *J* = 8 Hz, 2H)], 9.20 (bs, 1H, NH), 9.77 (bs, 1H, NH), 10.51 (bs, 1H, NH). ^13^C-NMR (100 MHz, DMSO-*d*_6_) δ, ppm: O-***C***H_3_ (56.2, 56.4, 60.5), 70.1 (O-***C***H_2_), Ar.C [105.8, 111.9, 115.0, 120.3, 125.1, 125.8, 126.5, 128.3, 130.2, 132.6, 137.6, 153.4, 161.7], 166.7 (***C***=O), 181.0 (C=S). HREIMS, C_25_H_27_N_3_O_6_S, *m*/*z* = 520.1522, [M + Na]^+^. Calcd. 520.1507.

*N-(p-Tolyl)-2-(4-(3,4,5-trimethoxybenzyloxy)benzoyl) Hydrazine Carbothioamide* (**3d**). White solid (Ethanol, 0.49 g, 86%), m.p. 165–167 °C. IR: 3321 (NH), 3220 (NH), 3163 (NH), 1662 (C=O), 1593 (C=C), 1234 (C=S), 1124 (O-CH_3_). ^1^H-NMR (400 MHz, DMSO-*d*_6_) δ, ppm: 2.28 (s, 3H, CH_3_), O-CH_3_ [3.66 (s, 3H), 3.78 (s, 6H)], 5.11 (s, 2H, O-CH_2_), Ar.H [6.79 (s, 2H), 7.08–7.11 (m, 4H), 7.27 (d, *J* = 5.6 Hz, 2H), 7.91 (d, *J* = 8.8 Hz, 2H)], 9.59 (s, 1H, NH), 9.71 (bs, 1H, NH), 10.36 (s, 1H, NH). ^13^C-NMR (100 MHz, DMSO-*d*_6_) δ, ppm: 21.0 (CH_3_), O-***C***H_3_ (56.4, 60.5), 70.1 (O-***C***H_2_), Ar.C [105.8, 114.8, 125.4, 126.4, 128.9, 130.3, 132.6, 134.6, 137.2, 137.6, 153.4, 161.6], 166.0 (***C***=O). HREIMS, C_25_H_27_N_3_O_5_S, *m*/*z*= 504.1570, [M + Na]^+^. Calcd. 504.1558.

*N-(m-Tolyl)-2-(4-(3,4,5-trimethoxybenzyloxy)benzoyl) Hydrazine Carbothioamide* (**3e**). White solid (Ethanol, 0.52 g, 90%), m.p. 89–101 °C. IR: 3321 (NH), 3220 (NH), 3170 (NH), 1663 (C=O), 1597 (C=C), 1238 (C=S), 1127 (O-CH_3_). ^1^H-NMR (400 MHz, DMSO-*d*_6_) δ, ppm: 2.29 (s, 3H, CH_3_), O-CH_3_ [3.66 (s, 3H), 3.78 (s, 6H)], 5.11 (s, 2H, O-CH_2_), Ar.H [6.80 (s, 2H), 6.98 (d, *J* = 7 Hz, 1H), 7.13 (d, *J* = 8 Hz, 2H), 7.20–7.22 (m, 2H), 7.37 (d, *J* = 6 Hz, 1H), 7.93 (d, *J* = 8 Hz, 2H), 9.61 (bs, 1H, NH), 9.72 (bs, 1H, NH), 10.37 (s, 1H, NH). ^13^C-NMR (100 MHz, DMSO-*d*_6_) δ, ppm: 21.4 (***C***H_3_), O-***C***H_3_ (56.4, 60.5), 70.1 (O-***C***H_2_), Ar.C [105.8, 114.8, 123.5, 125.4, 126.1, 126.9, 128.2, 130.3, 132.6, 137.6, 139.6, 153.4, 161.6], 166.0 (***C***=O), 181.6 (***C***=S). HREIMS, C_25_H_27_N_3_O_5_S, *m*/*z* = 504.1571, [M + Na]^+^. Calcd. 504.1558.

*N-(4-Nitrophenyl)-2-(4-(3,4,5-trimethoxybenzyloxy)benzoyl) Hydrazine Carbothioamide* (**3f**). Light yellow solid (Methanol, 0.57 g, 92%), m.p. 196–199 °C. IR: 3319 (NH), 3190 (NH), 3153 (NH), 1659 (C=O), 1593 (C=C), 1232 (C=S), 1123 (O-CH_3_), 1505, 1330 (NO_2_). ^1^H-NMR (400 MHz, DMSO-*d*_6_) δ, ppm: O-CH_3_ [3.66 (s, 3H), 3.78 (s, 6H)], 5.12 (s, 2H, O-CH_2_), Ar.H [6.80 (s, 2H), 7.14 (d, *J* = 8.5 Hz, 2H), 7.90–7.95 (m, 4H), 8.21 (d, *J* = 8.7 Hz, 2H)], 10.11 (bs, 2H, NH), 10.50 (bs, 1H, NH). ^13^C-NMR (100 MHz, DMSO-*d*_6_) δ, ppm: O-***C***H_3_ (56.4, 60.5), 70.1 (O-***C***H_2_), Ar.C [105.8, 114.9, 124.0, 125.1, 125.3, 129.3, 130.3, 132.6, 137.6, 146.2, 153.4, 161.7], 166.1 (***C***=O), 181.3 (***C***=S). HREIMS, C_24_H_24_N_4_O_7_S, *m*/*z* = 535.1257, [M + Na]^+^. Calcd. 535.1252.

#### 3.2.2. General Procedure for the Synthesis of 4-(4-Aryl)-3-(4-(3,4,5-trimethoxy benzyloxy) Phenyl)-1*H*-1,2,4-Triazole-5-(4*H*)-thiones **4a**–**f**

A mixture of arylthiosemicarbazide **3a**–**f** (0.80 mmol) and sodium hydroxide solution (4 M, 20 mL) was refluxed for 5–6 h. After cooling, 100 mL ice water was added. The solution pH was adjusted to 5–6 using diluted hydrochloric acid. The precipitate was filtered, washed with cold water, dried and recrystallized from a suitable solvent.

4*-(4-Chlorophenyl)-3-(4-(3,4,5-trimethoxybenzyloxy)phenyl)-1H-1,2,4-triazole-5-(4H)-thione (***4a**). White solid (ethanol, 0.31 g, 81%), m.p. 226–229 °C. IR: 3105 (NH), 1611 (C=N), 1330 (C-N), 1225 (C=S), 1125 (O-CH_3_), 702 (C-Cl). ^1^H-NMR δ, ppm: O-CH_3_ [3.65 (s, 3H), 3.76 (s, 6H)], 5.00 (s, 2H, O-CH_2_), 6.75 (s, 2H, ArH), 7.01 (d, *J* = 8.8 Hz, 2H, ArH), 7.26 (d, *J* = 8.8 Hz, 2H, ArH), 7.40 (d, *J* = 8.6 Hz, 2H, ArH), 7.58 (d, *J* = 8.6 Hz, 2H, ArH), 14.08 (s, 1H, NH). ^13^C-NMR δ, ppm: O-***C***H_3_ (56.3, 60.5), 70.1 (O-***C***H_2_), Ar.C [105.9, 115.3, 118.4, 129.9, 130.4, 131.2, 132.5, 134.0, 134.4, 137.6, 150.9 (***C***=N), 153.4, 160.3], 168.8 (***C***=S). HREIMS, C_24_H_22_ClN_3_O_4_S, *m*/*z* = 506.0915, 508.0901 [M + Na]^+^. Calcd. 506.0907, 508.0878.

*4-(4-Methoxyphenyl)-3-(4-(3,4,5-trimethoxybenzyloxy)phenyl)-1H-1,2,4-triazole-5-(4H)-thione* (**4b**). White solid (Ethanol: CHCl_3_, 0.30 g, 77%), m.p. 220–221 °C. IR: 3109 (NH), 1608 (C=N), 1329 (C-N), 1228 (C=S), 1122 (O-CH_3_). ^1^H-NMR (400 MHz, DMSO-*d*_6_) δ, ppm: O-CH_3_ [3.65 (s, 3H), 3.75 (s, 6H), 3.80 (s, 3H)], 4.99 (s, 2H, O-CH_2_), Ar.H [6.74 (s, 2H), 6.99 (d, *J* = 8 Hz, 2H), 7.03 (d, *J* = 8 Hz, 2H), 7.24–7.27 (m, 4H), 13.99 (s, 1H, NH). ^13^C-NMR (100 MHz, DMSO-*d*_6_) δ, ppm: O-***C***H_3_ (55.9, 56.3, 60.5), 70.1 (O-***C***H_2_), Ar.C [105.9, 114.9, 115.3, 118.7, 127.7, 130.2, 130.4, 132.5, 137.6, 151.1 (*C*=N), 153.4, 160.0, 160.2], 169.1 (***C***=S). HREIMS, C_25_H_25_N_3_O_5_S, *m*/*z* = 502.1408, [M + Na]^+^. Calcd. 502.1402.

*4-(2-Methoxyphenyl)-3-(4-(3,4,5-trimethoxybenzyloxy)phenyl)-1H-1,2,4-triazole-5-(4H)-thione* (**4c**). White solid (Ethyl acetate, 0.29 g, 75%), m.p. 239–242 °C. IR: 3271 (NH), 1614 (C=N), 1329 (C-N), 1242 (C=S), 1122 (O-CH_3_). ^1^H-NMR (400 MHz, DMSO-*d*_6_) δ, ppm:O-CH_3_ [3.57 (s, 3H), 3.64 (s, 3H), 3.74 (s, 6H)], 4.98 (s, 2H, O-CH_2_), Ar.H [6.73 (s, 2H), 6.97 (d, *J* = 8 Hz, 2H), 7.10 (t, *J* = 8 Hz, 1H), 7.15 (d, *J* = 8 Hz, 1H), 7.25 (d, *J* = 8 Hz, 2H), 7.39 (d, *J* = 8 Hz, 1H), 7.48 (d, *J* = 7.8 Hz, 1H)], 13.98 (s, 1H, NH). ^13^C-NMR (100 MHz, DMSO-*d*_6_) δ, ppm: O-***C***H_3_ (56.2, 56.3, 60.5), 70.1 (O-***C***H_2_), Ar.C [105.9, 113.4, 115.3, 119.0, 121.4, 123.6, 129.4, 131.0, 131.8, 132.5, 137.6, 151.4 (***C***=N), 153.4, 155.0, 160.2], 169.1 (***C***=S). HREIMS, C_25_H_25_N_3_O_5_S, *m*/*z* = 502.1416, [M + Na]^+^. Calcd. 502.1402.

*4-(p-Tolyl)-3-(4-(3,4,5-trimethoxybenzyloxy)phenyl)-1H-1,2,4-triazole-5-4H)-thione* (**4d**). White solid (Ethanol, 0.30 g, 81%), m.p. 103–107 °C. IR: 3174 (NH), 1609 (C=N), 1332 (C-N), 1232 (C=S), 1123 (O-CH_3_). ^1^H-NMR (400 MHz, DMSO-*d*_6_) δ, ppm: 2.37 (s, 3H, CH_3_), O-CH_3_ [3.67 (s, 3H), 3.76 (s, 6H)], 4.97 (s, 2H, O-CH_2_), Ar.H [6.71 (s, 2H), 6.95 (d, *J* = 8 Hz, 2H), 7.18 (d, *J* = 8 Hz, 2H), 7.24 (d, *J* = 8 Hz, 2H), 7.28 (d, *J* = 8 Hz, 2H), 13.97 (bs, 1H). ^13^C-NMR (100 MHz, DMSO-*d*_6_) δ, ppm: 21.2 (***C***H_3_), O-***C***H_3_ (56.3, 60.5), 69.9 (O-***C***H_2_), Ar.C [105.8, 114.9, 122.2, 128.9, 129.0, 129.5, 132.8, 135.6, 137.1, 137.5, 150.4 (***C***=N), 153.3, 158.6], 168.9 (***C***=S). HREIMS, C_25_H_25_N_3_O_4_S, *m*/*z* = 486.1467, [M + Na]^+^. Calcd. 486.1453.

*4-(m-Tolyl)-3-(4-(3,4,5-trimethoxybenzyloxy)phenyl)-1H-1,2,4-triazole-5-(4H)-thione* (**4e**). White solid (Methanol, 0.27 g, 74%), m.p. 187–190 °C. IR: 3110 (NH), 1609 (C=N), 1328 (C-N), 1227 (C=S), 1124 (O-CH_3_). ^1^H-NMR (400 MHz, DMSO-*d*_6_) δ, ppm: 2.30 (s, 3H, CH_3_), O-CH_3_ [3.64 (s, 3H), 3.74 (s, 6H)], 4.97 (s, 2H, O-CH_2_), Ar.H [6.73 (s, 2H), 6.97 (d, *J* = 8 Hz, 2H), 7.10 (d, *J* = 7 Hz, 1H), 7.16 (s, 1H), 7.25 (d, *J* = 8 Hz, 2H), 7.30 (d, *J* = 7 Hz, 1H), 7.37 (dd, *J* = 7 Hz, 1H), 14.04 (s, 1H, NH). ^13^C-NMR (100 MHz, DMSO-*d*_6_) δ, ppm: 21.2 (***C***H_3_), O-***C***H_3_ (56.3, 60.5), 70.1 (O-***C***H_2_), Ar.C [105.8, 115.3, 118.6, 126.2, 129.5, 129.6, 130.2, 130.6, 132.5, 135.1, 137.6, 139.4, 150.9 (***C***=N), 153.3, 160.2], 168.9 (***C***=S). HREIMS, C_25_H_25_N_3_O_4_S, *m*/*z* = 486.1469, [M + Na]^+^. Calcd. 486.1453.

*4-(3,4,5-trimethoxybenzyloxy)benzoic Acid* (**4f**). White solid (Methanol, 0.18 g, 69%), m.p. 146–148 °C. IR: 2938 (OH, m), 1680 (C=O), 1241 (C=C), 1126 (O-CH_3_). ^1^H-NMR (400 MHz, DMSO-*d*_6_) δ, ppm: O-CH_3_ [3.66 (s, 3H), 3.78 (s, 6H)], 5.09 (s, 2H, O-CH_2_), Ar.H [6.79 (s, 2H), 7.10 (d, *J* = 8.8 Hz, 2H), 7.90 (d, *J* = 8.8 Hz, 2H)], 12.62 (bs, 1H, OH). ^13^C-NMR (100 MHz, DMSO-*d*_6_) δ, ppm: O-***C***H_3_ (56.4, 60.5), 70.2 (O-***C***H_2_), Ar.C [105.9, 115.1, 123.6, 131.8, 132.5, 137.7, 153.4, 162.4], 167.4 (***C***=O). HREIMS, C_17_H_18_O_6_, *m*/*z* = 341.1001, [M + Na]^+^. Calcd. 341.0995.

#### 3.2.3. *5-(4-(3,4,5-Trimethoxybenzyloxy)phenyl)-1,3,4-oxadiazole-2-(3H)-thione* (**5**)

A mixture of compound **2** (1.20 g, 3.6 mmol), anhydrous potassium hydroxide (0.6 g, 10.7 mmol), carbon disulfide (0.91 mL, 15.1 mmol) and absolute ethanol (15 mL) was refluxed for 24 h. The solvent was removed in vacuum and the residue was poured on 100 mL ice water and acidified with 5% HCl. The precipitate was collected, washed with water, dried and crystallized from ethanol to give a white solid (1.16 g, 86%), m.p. 198–200 °C. IR: 3173 (NH), 2967 (CH_Ar_), 2841 (CH_aliph_.), 1599 (C=N), 1070 (O-CH_3_), 1130 (C-N). ^1^H-NMR δ, ppm: O-CH_3_ [3.67 (s, 3H), 3.79 (s, 6H)], 5.12 (s, 2H, O-CH_2_), 6.80 (s, 2H, ArH), 7.21 (d, *J* = 8.5 Hz, 2H, ArH), 7.83 (d, *J* = 8.5 Hz, 2H, Ar.H), 14.60 (bs, 1H, NH).^13^C-NMR δ, ppm: O-***C***H_3_ (56.4, 60.5), 70.3 (O-***C***H_2_), Ar.C [105.9, 115.4, 116.1, 128.4, 132.3, 137.7, 153.4, 161.0(C-O)], 161.8 (***C***=N), 177.6 (***C***=S). HREIMS, C_18_H_18_N_2_O_5_S, *m*/*z* = 375.1018, [M + H]^+^. Calcd. 375.1007.

#### 3.2.4. General Procedure for the Synthesis of 5-Aryl-2-(chloromethyl)-1,3,4-oxadiazoles (**R’c**–**f**)

A mixture of aryl acid hydrazide (10 mmol), chloroacetic acid (1.0 g, 10.5 mmol) and POCl_3_ (7 mL, 73 mmol) was refluxed for 6 h. The reaction mixture was poured onto crushed ice. The resulting precipitate was filtered, washed with saturated aqueous sodium bicarbonate and water, and then dried and recrystallized from ethanol [25,57].

*2-(4-Bromophenyl)-5-(chloromethyl)-1,3,4-oxadiazole* (**R′c**). Pale red solid (2.10 g, 77%), m.p. 180–184 °C (lit. 214–215 °C) [57]. ^1^H-NMR (400 MHz, DMSO-*d*_6_) δ, ppm: 5.13 (s, 2H, CH_2_), Ar-H [7.81 (d, *J* = 8.2 Hz, 2H), 7.94 (d, *J* = 8.2 Hz, 2H)]. 1^3^C-NMR (100 MHz, DMSO-*d*_6_) δ, ppm: 33.7 (CH_2_), Ar-C [122.5, 126.5, 129.1, 133.1], (C=N) [163.5, 164.8].

*2-(Chloromethyl)-5-(4-chlorophenyl)-1,3,4-oxadiazole* (**R′d**). Pale brown solid (1.83 g, 80%), m.p. 71–76 °C (lit. 82–83 °C) [57]. ^1^H-NMR (400 MHz, DMSO-*d*_6_) δ, ppm: 5.15 (s, 2H, CH_2_), Ar-H [7.68 (d, *J* = 8.3 Hz, 2H), 8.02 (d, *J* = 8.3 Hz, 2H)]. ^13^C-NMR (100 MHz, DMSO-*d*_6_) δ, ppm: 33.7 (CH_2_), Ar-C [122.2, 128.9, 130.2, 137.6], (C=N) [163.5, 164.7].

*2-(Chloromethyl)-5-p-tolyl-1,3,4-oxadiazole* (**R′e**). Pale pink solid (1.79 g, 86%), m.p. 104–106 °C (lit. 116–118 °C) [57]. ^1^H-NMR (400 MHz, DMSO-*d*_6_) δ, ppm: 2.38 (s, 3H, CH_3_), 5.12 (s, 2H, CH_2_), Ar-H [7.39 (d, *J* = 8.1 Hz, 2H), 7.88 (d, *J* = 8.1 Hz, 2H)]. ^13^C-NMR (100 MHz, DMSO-*d*_6_) δ, ppm: 21.5 (CH_3_), 33.7 (CH_2_), Ar-C [120.5, 127.1, 130.5, 143.1], (C=N) [163.1, 165.5].

*2-(Chloromethyl)-5-(4-methoxypheyl)-1,3,4-oxadiazole* (**R′f**). Pale pink solid (2.0 g, 93%), m.p. 91–94 °C (lit. 92–93 °C) [57]. ^1^H-NMR (400 MHz, DMSO-*d*_6_) δ, ppm: 3.85 (s, 3H, OCH_3_), 5.11 (s, 2H, CH_2_), Ar-H [7.13 (d, *J* = 8.9 Hz, 2H), 7.93 (d, *J* = 8.9 Hz, 2H)]. ^13^C-NMR (100 MHz, DMSO-*d*_6_) δ, ppm: 33.8 (CH_2_), 56.0 (O***C***H_3_), Ar-C [115.4, 115.6, 129.0, 162.7] (C-O), (C=N) [162.8, 165.4].

#### 3.2.5. General Procedure for the Synthesis of 2-(4-Arylthio)-5-(4-(3,4,5-trimethoxybenzyloxy) Phenyl)-1,3,4-oxadiazoles (**6a**–**f**)

Compound **5** (0.50 g, 1.3 mmol) was dissolved in acetone (25 mL) and anhydrous potassium carbonate (0.18 g, 1.3 mmol) was added, followed by alkyl halide (1.34 mmol). The mixture was refluxed for 24 h and the solvent was removed in vacuum. Water was added and mixture extracted with ethyl acetate. The organic layer was washed with water, dried over sodium sulfate, filtered, and concentrated in vacuum. The precipitate was recrystallized from methanol.

*2-(4-Bromobenzylthio)-5-(4-(3,4,5-trimethoxybenzyloxy)phenyl)-1,3,4-oxadiazole* (**6a**). Pale white solid (0.53 g, 75%), m.p. 104–106 °C. IR: 2937 (CH_Ar_), 2832 (CH_aliph_.), 1591 (C=N), 1069 (O-CH_3_), 528 (C-Br). ^1^H-NMR δ, ppm: O-CH_3_ [3.67 (s, 3H), 3.79 (s, 6H)], 4.54 (s, 2H, S-CH_2_), 5.11 (s, 2H, O-CH_2_), Ar.H [6.81 (s, 2H, ArH), 7.21 (d, *J* = 8.6 Hz, 2H, ArH), 7.43 (d, *J* = 8.2 Hz, 2H, ArH), 7.53 (d, *J* = 8.2 Hz, 2H, ArH), 7.89 (d, *J* = 8.6 Hz, 2H, ArH].^13^C-NMR δ, ppm: 35.6 (S-***C***H_2_), O-***C***H_3_ (56.4, 60.5), 70.3 (O-***C***H_2_), (Ar C) [105.9, 116.0, 116.1, 121.4, 128.7, 131.7, 131.9, 132.4, 136.8, 137.7, 153.4, 161.7], (***C***=N) [162.8, 165.7]. HREIMS, C_25_H_23_BrN_2_O_5_S, *m*/*z* = 565.0407, 567.0390 [M + Na]^+^. Calcd. 565.0399, 567.0378.

*5-(4-(3,4,5-trimethoxybenzyloxy)phenyl)-1-(4-Bromophenyl)-2-thioethyl-one-1,3,4-oxadiazole* (**6b**). Pale white solid (0.57 g, 77%), m.p. 120–122 °C. IR: 2944 (CH_Ar_), 2838 (CH_aliph_.), 1594 (C=N), 1607 (C=O), 1070 (O-CH_3_). ^1^H-NMR (400 MHz, DMSO-*d*_6_) δ, ppm: O-CH_3_ [3.67 (s, 3H), 3.78 (s, 6H)], 5.11 (s, 2H, O-CH_2_), 5.14 (s, 2H, S-CH_2_), Ar.H [6.80 (s, 2H), 7.21 (d, *J* = 8.8 Hz, 2H), 7.80 (d, *J* = 8.5 Hz, 2H), 7.88 (d, *J* = 8.8 Hz, 2H), 8.00 (d, *J* = 8.5 Hz, 2H)]. ^13^C-NMR (100 MHz, DMSO-*d*_6_) δ, ppm: 49.1 (S-***C***H_2_), O-***C***H_3_ (56.4, 60.5), 70.3 (O-***C***H_2_), Ar.C [105.9, 115.9, 116.1, 128.6, 128.7, 130.9, 132.4, 132.5, 134.5, 137.7, 153.4, 161.6, 162.8, 165.5], 192.6 (***C***=O). HREIMS, C_26_H_23_BrN_2_O_6_S, *m*/*z* = 593.0362, 595.0339 [M + Na]^+^. Calcd. 593.0348, 595.0327.

*2-(4-Bromophenyl)-5-((5-(4-(3,4,5-trimethoxybenzyloxy)phenyl)-1,3,4-oxadiazol-2-ylthio)methyl)-1,3,4-oxadiazole* (**6c**). Pale reddish brown solid (0.54 g, 68%), m.p. 130–132 °C. IR: 2925 (CH_Ar_), 2818 (CH_aliph_.), 1591 (C=N), 1124 (O-CH_3_), 525 (Br-C). ^1^H-NMR (400 MHz, DMSO-*d*_6_) δ, ppm: O-CH_3_ [3.67 (s, 3H), 3.79 (s, 6H)], 4.95 (s, 2H, S-CH_2_), 5.11 (s, 2H, O-CH_2_), Ar.H [6.81 (s, 2H), 7.21 (d, *J* = 8.2 Hz, 2H), 7.66 (d, *J* = 8.4 Hz, 2H), 7.89–7.95 (m, 4H)].^13^C-NMR (100 MHz, DMSO-*d*_6_) δ, ppm: 26.4 (S-***C***H_2_), O-***C***H_3_ (56.4, 60.5), 70.3 (O-***C***H_2_), Ar.C [105.9, 115.8, 116.1, 122.7, 126.3, 128.8, 132.3, 133.1, 137.7, 153.4, 161.5], (***C***=N) [161.8, 164.0, 164.4, 166.1]. HREIMS, C_27_H_23_BrN_4_O_6_S, *m*/*z* = 633.0425, 635.0402 [M + Na]^+^. Calcd. 633.0408, 635.0387.

*2-(4-Chlorophenyl)-5-((5-(4-(3,4,5-trimethoxybenzyloxy)phenyl)-1,3,4-oxadiazol-2-ylthio)methyl)-1,3,4-oxadiazole* (**6d**). Pale brown solid (0.51 g, 69%), m.p. 140–142 °C. IR: 2926 (CH_Ar_), 2833 (CH_aliph_.), 1591 (C=N), 1125 (O-CH_3_), 698 (Cl-C). ^1^H-NMR (400 MHz, DMSO-*d*_6_) δ, ppm: O-CH_3_ [3.67 (s, 3H), 3.79 (s, 6H)], 4.95 (s, 2H, S-CH_2_), 5.11 (s, 2H, O-CH_2_), Ar.H [6.81 (s, 2H), 7.21 (d, *J* = 8.8 Hz, 2H), 7.66 (d, *J* = 8.5 Hz, 2H), 7.89–7.95 (m, 4H). ^13^C-NMR (100 MHz, DMSO-*d*_6_) δ, ppm: 26.4 (S-***C***H_2_), O-***C***H_3_ (56.4, 60.5), 70.3 (O-***C***H_2_), Ar.C [105.9, 115.8, 116.1, 122.4, 128.7, 128.82, 130.1, 132.3, 137.4, 137.7, 153.4, 161.5], (***C***=N) [161.8, 163.9, 164.3, 166.1]. HREIMS, C_27_H_23_ClN_4_O_6_S, *m*/*z* = 589.0927, 591.0905 [M + Na]^+^. Calcd. 589.0913, 591.0884.

*2-p-Tolyl-5-((5-(4-(3,4,5-trimethoxybenzyloxy)phenyl)-1,3,4-oxadiazol-2-ylthio)methyl)-1,3,4-oxadiazole* (**6e**). Pale white solid (0.50 g, 71%), m.p. 128–130 °C. IR: 2939 (CH_Ar_), 2827 (CH_aliph_.), 1598 (C=N), 1121 (O-CH_3_). ^1^H-NMR (400 MHz, DMSO-*d*_6_) δ, ppm: 2.38 (s, 3H, CH_3_), O-CH_3_ [3.66 (s, 3H), 3.78 (s, 6H)], 4.92 (s, 2H, S-CH_2_), 5.11 (s, 2H, O-CH_2_), Ar.H [6.80 (s, 2H), 7.21 (d, *J* = 8.9 Hz, 2H), 7.37 (d, *J* = 8.2 Hz, 2H), 7.81 (d, *J* = 8.2 Hz, 2H), 7.91 (d, *J* = 8.9 Hz, 2H)]. ^13^C-NMR (100 MHz, DMSO-*d*_6_) δ, ppm: 21.6 (***C***H_3_), 26.4 (S-***C***H_2_), O-***C***H_3_ (56.4, 60.5), 70.3 (O-***C***H_2_), Ar.C [105.9, 115.8, 116.1, 120.7, 126.9, 128.8, 130.5, 132.3, 137.7, 142.9, 153.4, 161.5], (***C***=N) [161.8, 163.4, 165.1, 166.2]. HREIMS, C_28_H_26_N_4_O_6_S, *m*/*z* = 569.1471, [M + Na]^+^. Calcd. 569.1459.

*2-(4-Methoxyphenyl)-5-((5-(4-(3,4,5-trimethoxybenzyloxy)phenyl)-1,3,4-oxadiazol-2-ylthio)methyl)-1,3,4-oxadiazole* (**6f**). White solid (0.51 g, 70%), m.p. 118–120 °C. IR: 2950 (CH_Ar_), 2833 (CH_aliph_.), 1596 (C=N), 1125 (O-CH_3_). ^1^H-NMR (400 MHz, DMSO-*d*_6_) δ, ppm: O-CH_3_ [3.66 (s, 3H), 3.78 (s, 6H), 3.84 (s, 3H)], 4.91 (s, 2H, S-CH_2_), 5.11 (s, 2H, O-CH_2_), Ar.H [6.80 (s, 2H), 7.11 (d, *J* = 8.7 Hz, 2H), 7.21 (d, *J* = 8.7 Hz, 2H), 7.86 (d, *J* = 8.6 Hz, 2H), 7.91 (d, *J* = 8.6 Hz, 2H)]. ^13^C-NMR (100 MHz, DMSO-*d*_6_) δ, ppm: 26.4 (S-***C***H_2_), O-***C***H_3_ (56.0, 56.4, 60.5), 70.3 (O-***C***H_2_), Ar.C [105.9, 115.4, 115.8, 115.82, 116.1, 128.8, 128.86, 132.3, 137.7, 153.4, 161.5, 161.8], (***C***=N) [162.6, 163.1, 165.0, 166.2]. HREIMS, C_28_H_26_N_4_O_7_S, *m*/*z* = 585.1420, [M + Na]^+^. Calcd. 585.1408.

#### 3.2.6. *In Vitro* Antioxidant Activity Assays

##### DPPH Radical Scavenging Activities

The antioxidant activity was performed using a modified DPPH radical scavenging protocol reported by Brand-Williams [34]. The test compounds were initially dissolved in dimethyl sulfoxide (DMSO). The reaction mixture was prepared by mixing 195 µL of a 100 μM methanolic solution of DPPH with 50 μL of the test compounds at different concentrations (0–1000 μg/mL). BHT and ascorbic acid (AA) were used as positive controls and were run in parallel. After 30 min of incubation in the dark at room temperature, the absorbance of the reaction mixture was read at 515 nm. As result of the decreased absorbance, the colour of the reaction mixture changed from purple to yellow. The radical scavenging activity was calculated according to the following equation:

Scavenging activity, % = [(A_0_ – A_1_)/A_0_] × 100 (1)

where A_0_ is the absorbance of the DPPH radical without a sample or standard; and A_1_ is the absorbance of the DPPH radical with a sample or standard. IC_50_ values which represent the efficient concentration of the standards and samples that inhibit 50% of the DPPH radicals were calculated and expressed in µg/mL [58,59,60]. Results were also expressed as percentage inhibition of the DPPH radicals at 125 µg/mL concentration.

##### Ferric Reducing Antioxidant Power Activity (FRAP)

The FRAP assay was determined with slight modifications, according to the method described by Benzie and Strain [56]. Three reagents were initially prepared: 300 mM acetate buffer (pH = 3.6), 10 mM 2,4,6- tripyridyl-s-triazine (TPTZ) in 40 mM HCl and 20 mM FeCl_3_. The FRAP working solution was freshly prepared by mixing the acetate buffer with the TPTZ solution in 20 mM FeCl_3_ in a ratio of 10:1:1 (*v*/*v*/*v*), respectively. Five microliters of the sample or standard was mixed with 300 μL of the FRAP reagent, followed by a 30 min incubation period at 37 °C. Subsequently, the increase in the absorbance of the coloured product was measured at 595 nm. The results were calculated, based on a calibration curve plotted using iron sulfate (FeSO_4_) (0–1 mM).

##### Computational Details

All calculations were performed using the Gaussian 09 program [61,62]. The geometry of each molecule and radical in the DMSO solvent-phase was optimized using the DFT method with a UB3LYP function without any constraints. The calculations were performed using B3LYP/6-311++G** (d,p) level of theory to perform the most reliable optimization of the geometrical parameters and to calculate physical descriptors characterizing their antioxidant ability in particular, the homolytic bond dissociation enthalpy (BDE), HOMO orbital distribution and spin density.

## 4. Conclusions

In conclusion, this study reports the preparation of new heterocyclic compounds and their analogs bearing the 3,4,5-trimethoxybenzyl moiety and evaluates the differences between their antioxidant activities. Thiosemicarbazide compounds **3a**–**f** showed the highest activity, more than their cyclized molecules, the triazoles **4a**–**e**. Electron withdrawing substituents enhanced the antioxidant activities in thiosemicarbazides and *para*-substituted derivatives were more active than *ortho* ones. This is believed to be due to the intramolecular hydrogen bond which also disfavours the DPPH scavenging activity of the *ortho*-hydroxy substituent. The S-alkylation of compound **5**, 2-thioalkyl-1,3,4-oxadiazoles practically eliminated antioxidant activity, which indicated that an unalkylated thioamide group (with an ionizable proton) is a good free radical scavenger.

## Figures and Tables

**Table 1:** DPPH radical scavenging activity and FRAP values of the synthesized compounds.

Compounds	DPPH IC_50_ (µg/mL) ^a^	Radical Scavenging Maximum Inhibition % ^b^	FRAP µM ^c^
**3a**	42 ± 1	74 ± 1.7	1140 ± 33
**3b**	38 ± 1	76 ± 3	2476 ± 64
**3c**	43 ± 4	87 ± 2.1	1566 ± 26
**3d**	32 ± 1	85 ± 2.6	1905 ± 42
**3e**	52 ± 2	82 ± 2.1	2519 ± 103
**3f**	29 ± 2	89 ± 1.9	838 ± 38
**4a**	154 ± 11	46 ± 1.6	629 ± 13
**4b**	162 ± 4	45 ± 1	189 ± 18
**4c**	274 ± 3	34 ± 2	634 ± 22
**4d**	157 ± 3	31 ± 1.8	213 ± 29
**4e**	389 ± 2	46 ± 1.3	365 ± 25
**4f**	201 ± 14	34 ± 2.1	370 ± 10
**5**	45 ± 2	73 ± 1.6	1688 ± 12
**6a**	NA	17 ± 0.9	158 ± 32
**6b**	NA	26 ± 1.4	238 ± 27
**6c**	NA	24 ± 3.1	256 ± 15
**6d**	NA	28 ± 1.2	193 ± 14
**6e**	NA	32 ± 2.3	268 ± 13
**6f**	NA	38 ± 2.1	157 ± 17
AA*	42 ± 2	90 ± 1.4	2231 ± 87
**BHT**	119 ± 2	51 ± 1	1274 ± 44

Results are expressed as a mean ± standard deviation (*n* = 3). **^a^** DPPH radical scavenging activities are expressed as IC_50_ concentrations of the compounds (µg/mL) required to inhibit 50% of the radicals; **^b^** The maximum inhibition of radical scavenging is the inhibition at 125 µg/mL concentration; **^c^** FRAP, ferric reducing antioxidant power; NA: did not reach 50% inhibition of the DPPH radicals at the concentrations used in this study; AA*: Ascorbic acid.

**Table 2:** Calculated properties for compound **3f**.

Compound	BDE (kcal/mol)	Spin Density
**3f-r1**	38.4	0.395
**3f-r2**	39.8	0.445
**3f-r3**	42.2	0.789

**Table 3:** Calculated properties for compounds **6a** and **6b**.

Compound		BDE (kcal/mol)	Spin Density
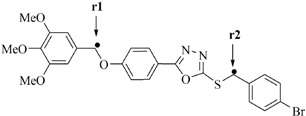	**6a-r1**	290.0	0.985
	**6a-r2**	278.2	0.983
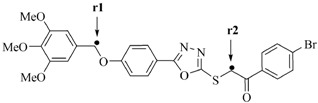	**6b-r1**	274.5	0.921
	**6b-r2**	275.9	0.979

## Supplementary Materials

Supplementary materials can be accessed at: http://www.mdpi.com/1420-3049/21/2/224/s1.
