# Supplementary material for: Conjugated Oligo-Aromatic Compounds Bearing a 3,4,5-Trimethoxy Moiety: Investigation of Their Antioxidant Activity Correlated with a DFT Study
Source: Molecules. 2016 Feb 17;21(2):224. doi: 10.3390/molecules21020224 (PMC6274057; doi:10.3390/molecules21020224)
Supplement: Supplementary file 1 [file molecules-21-00224-s001.pdf]

Molecules **2016**, *21*, 224; doi:10.3390/molecules21020224

Supplementary Material: Conjugated Oligo-Aromatic Compound Bearing a 3,4,5-Trimethoxy Moiety: Investigation of Their Antioxidant Activity Correlated with a DFT Study

Huda. S. Kareem [1,2], Nurdiana Nordin [1], Thorsten Heidelberg [1], Azlina Abdul-Aziz [3] and Azhar Ariffin [1,*]

^{1}H- and ^{13}C-NMR

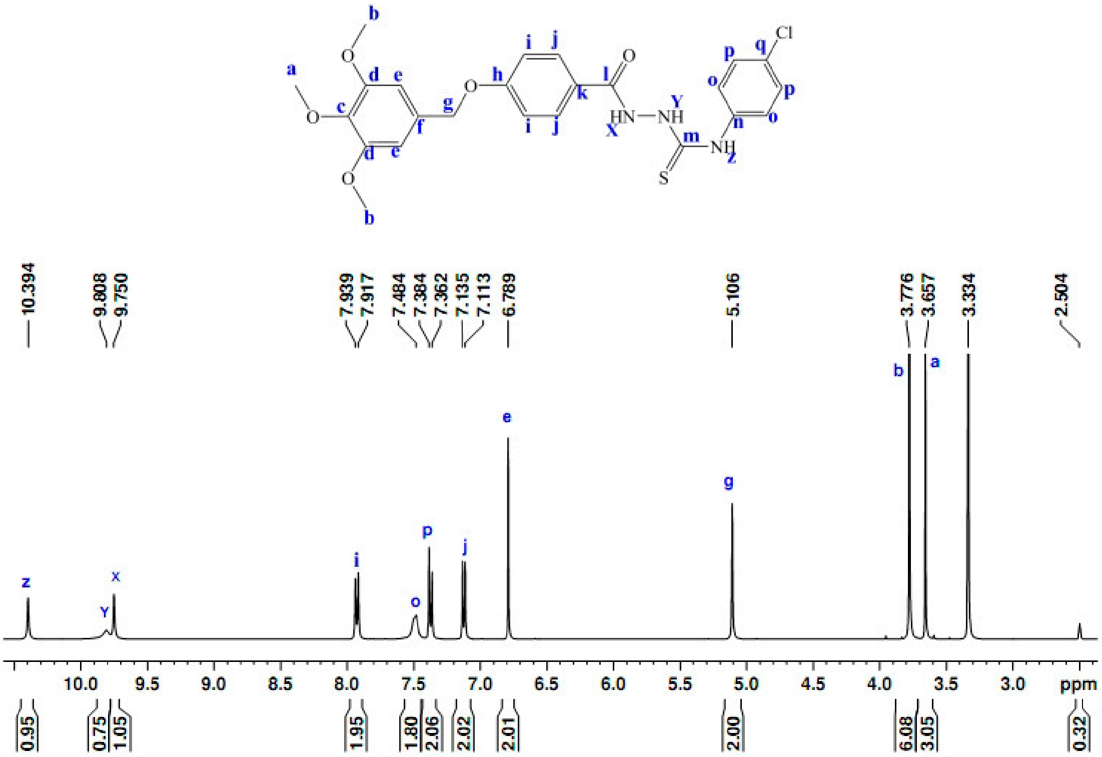

Figure S1. ^{1}H spectrum (DMSO-d_6, 400 MHz) of **3a**.

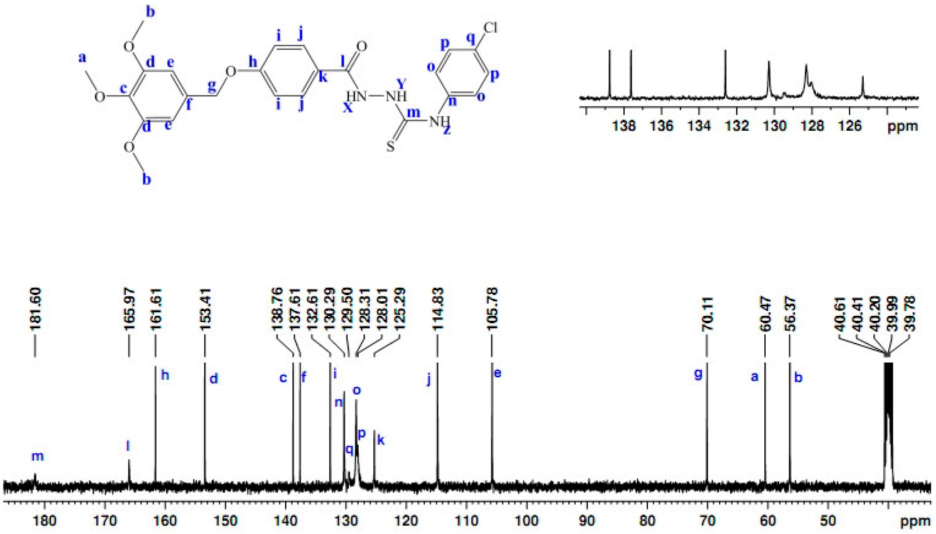

Figure S2. ^{13}C spectrum (DMSO-d_6, 100 MHz) of **3a**.

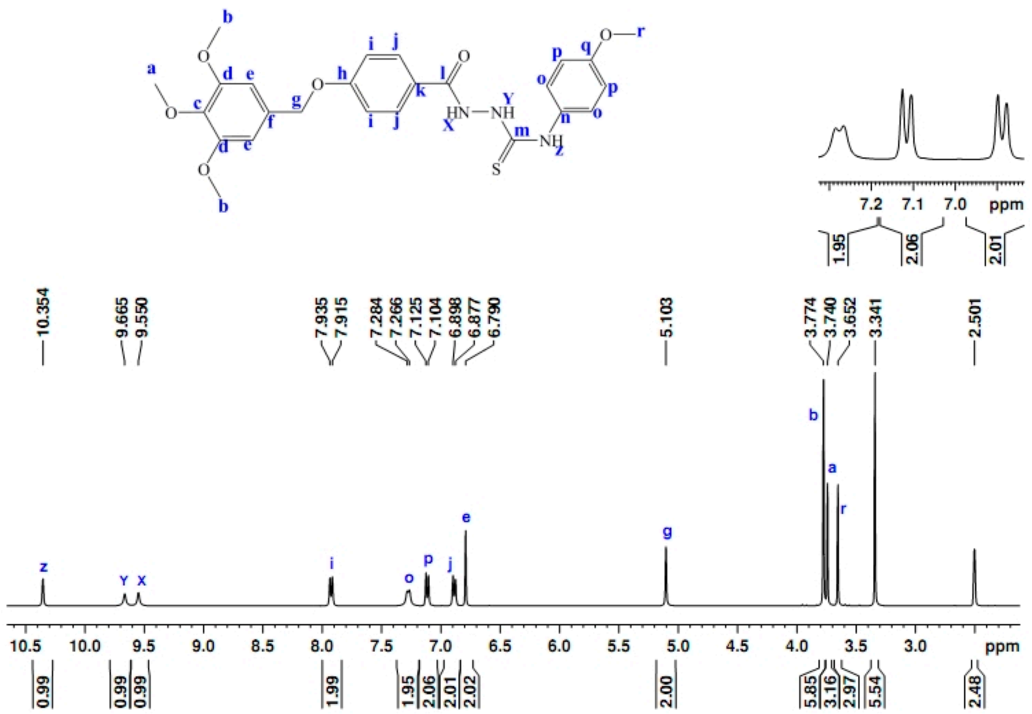

Figure S3. ^{1}H spectrum (DMSO-d_6, 400 MHz) of **3b.**

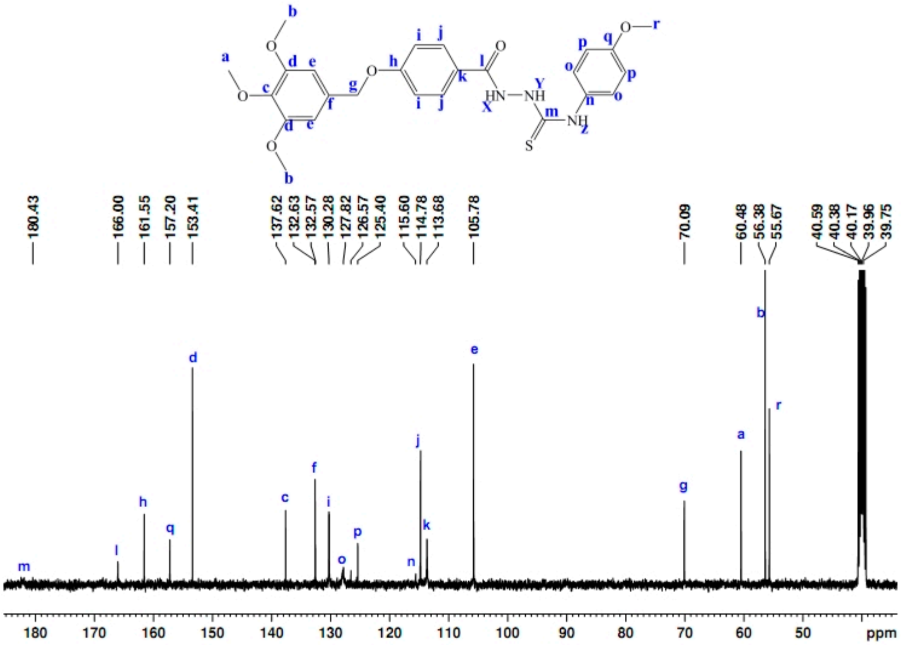

Figure S4. ^{13}C spectrum (DMSO-d_6, 100 MHz) of **3b**.

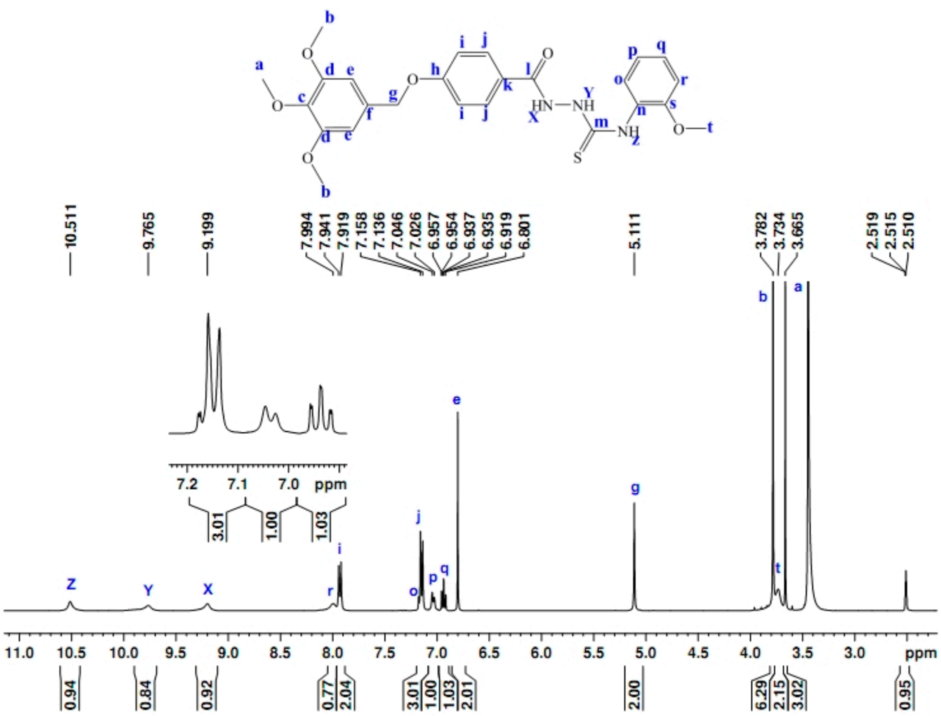

Figure S5. ^{1}H spectrum (DMSO-d_6, 400 MHz) of **3c.**

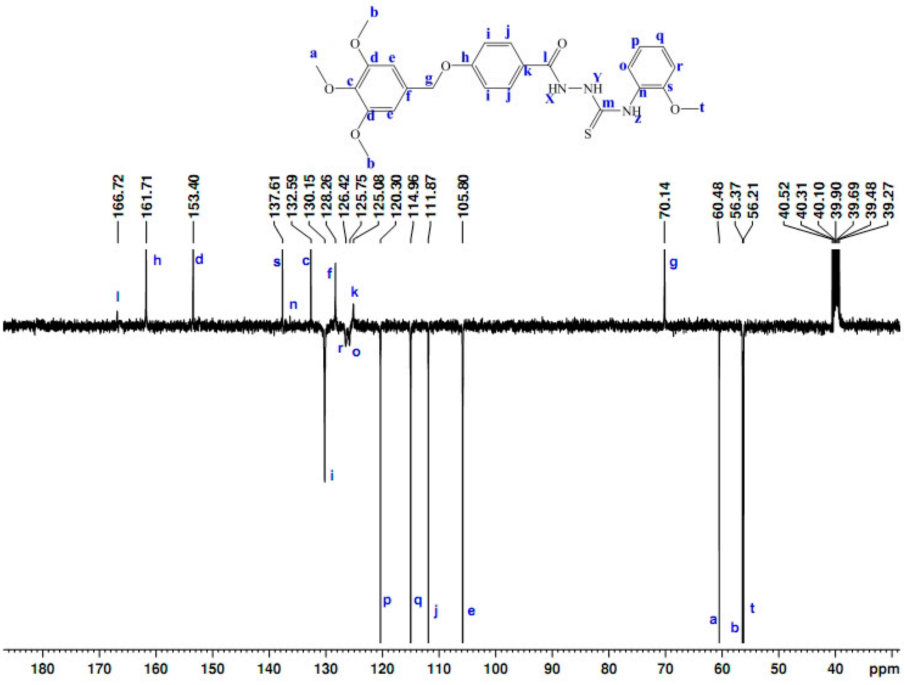

Figure S6. ^{13}C spectrum (DMSO-d_6, 100 MHz) of **3c.**

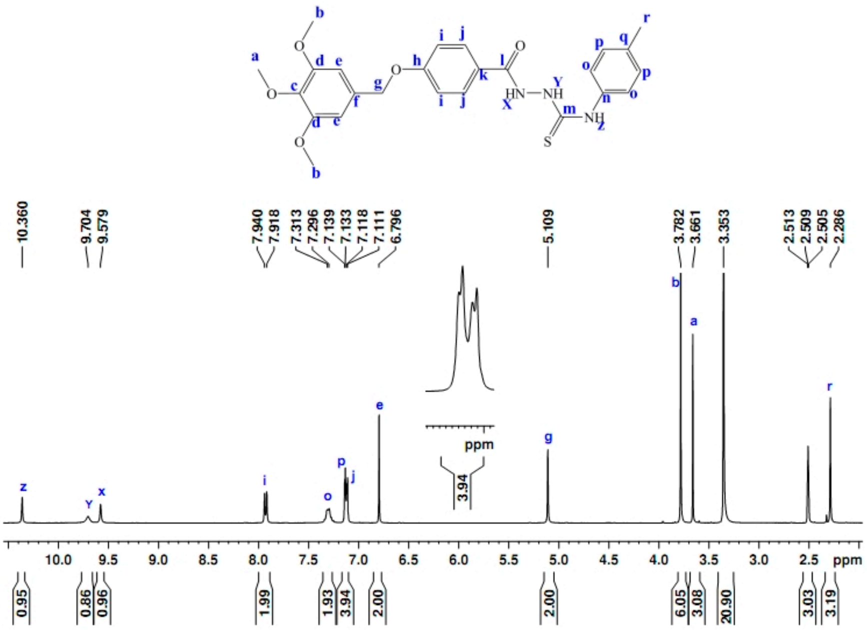

Figure S7. ^{1}H spectrum (DMSO-d_6, 400 MHz) of **3d.**

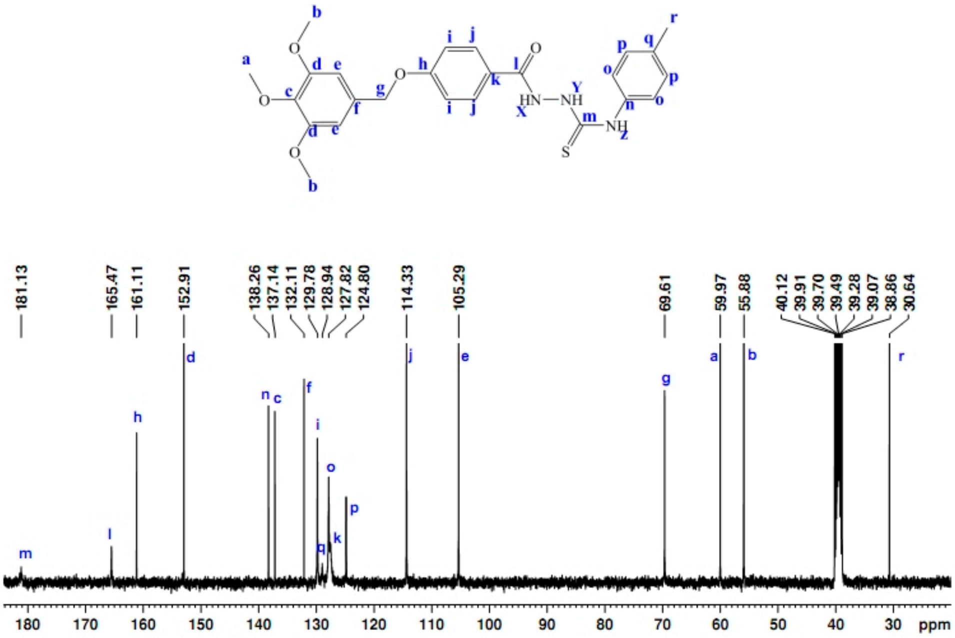

Figure S8. ^{13}C spectrum (DMSO-d_6, 100 MHz) of **3d.**

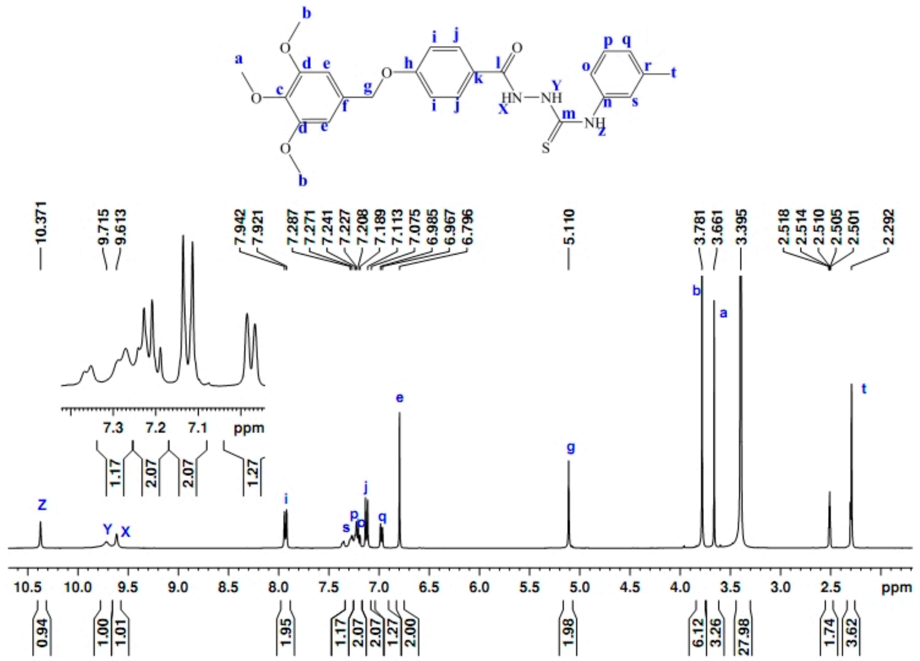

Figure S9. ^{1}H spectrum (DMSO-d_6, 400 MHz) of **3e.**

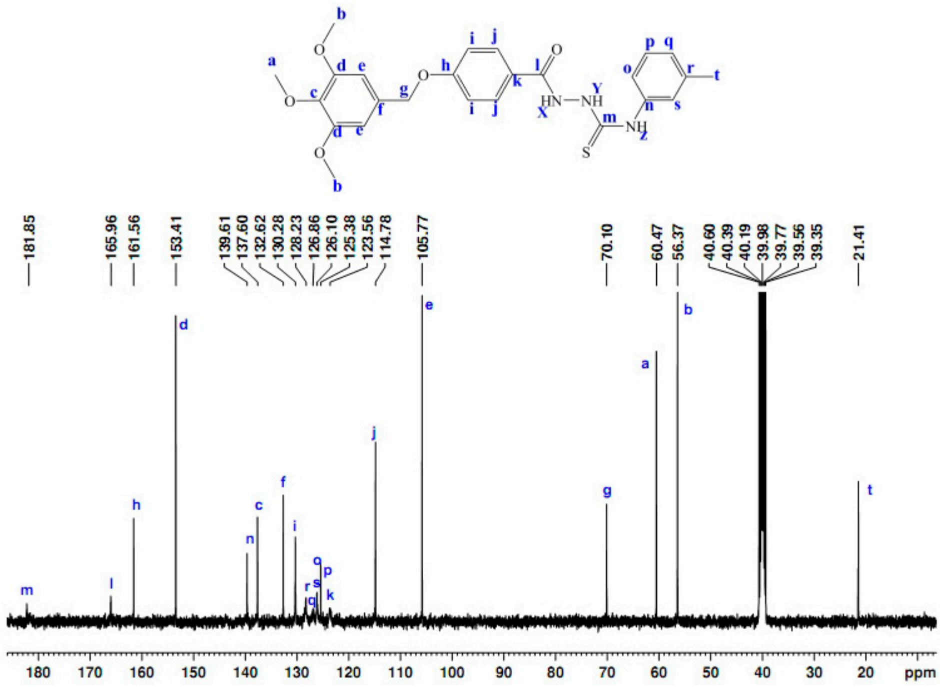

Figure S10. ^{13}C spectrum (DMSO-d_6, 100 MHz) of **3e.**

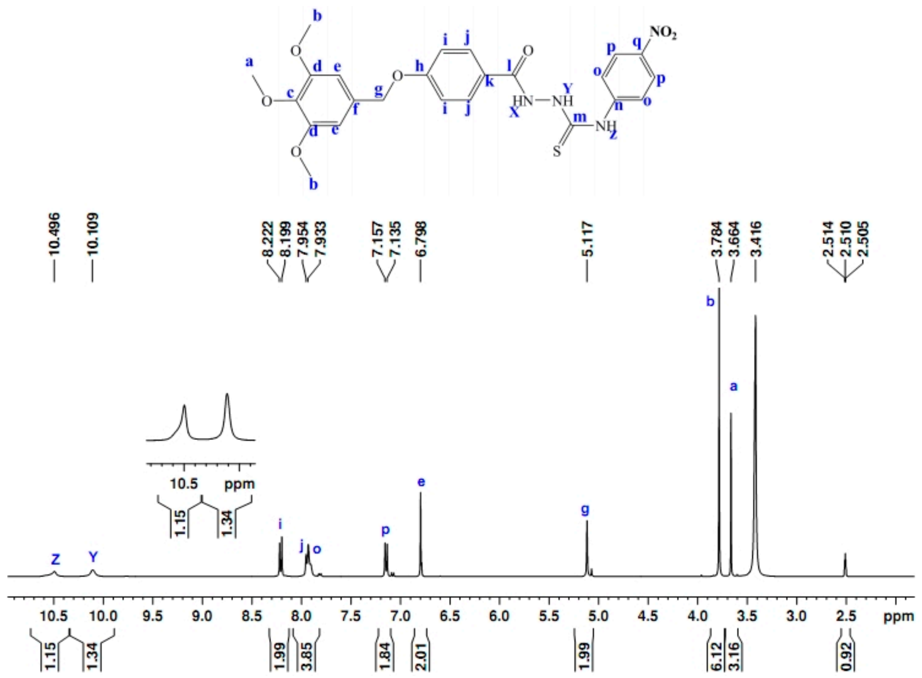

Figure S11. ^{1}H spectrum (DMSO-d_6, 400 MHz) of **3f.**

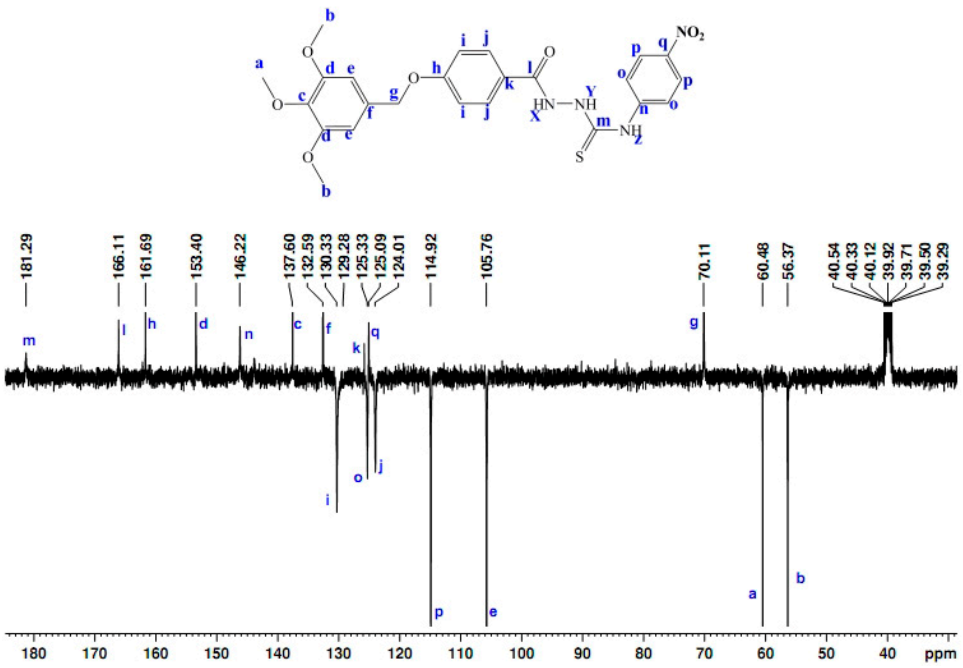

Figure S12. ^{13}C spectrum (DMSO-d_6, 100 MHz) of **3f.**

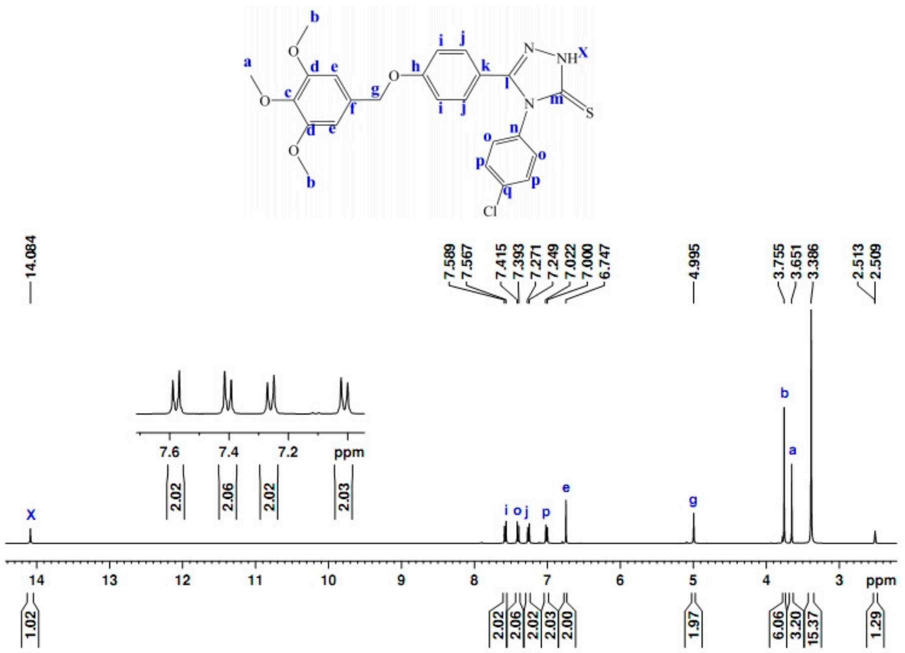

Figure S13. ^{1}H spectrum (DMSO-*d*6, 400 MHz) of **4a.**

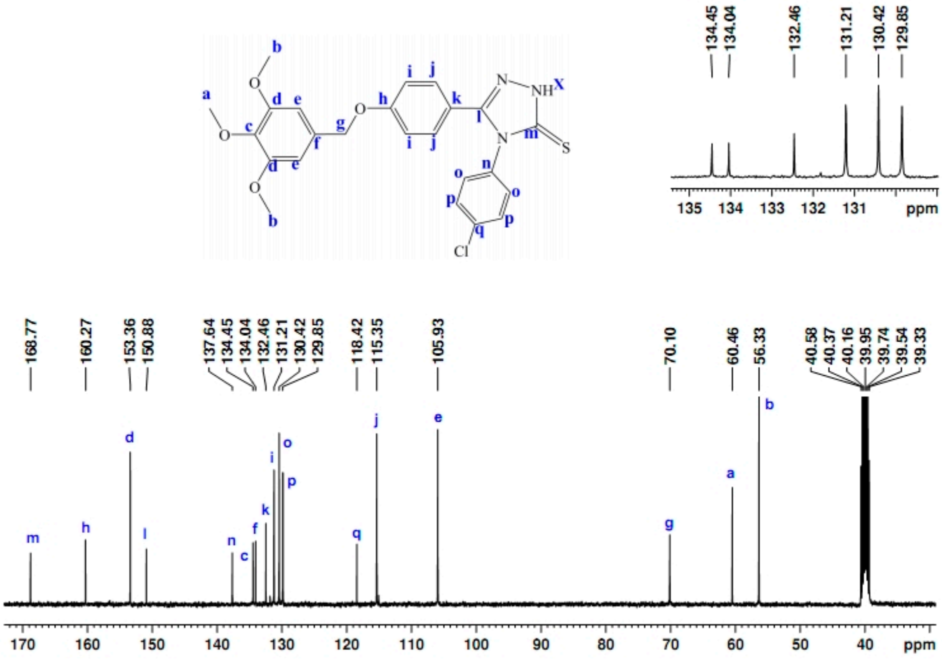

Figure S14. ^{13}C spectrum (DMSO-*d*6, 100 MHz) of **4a.**

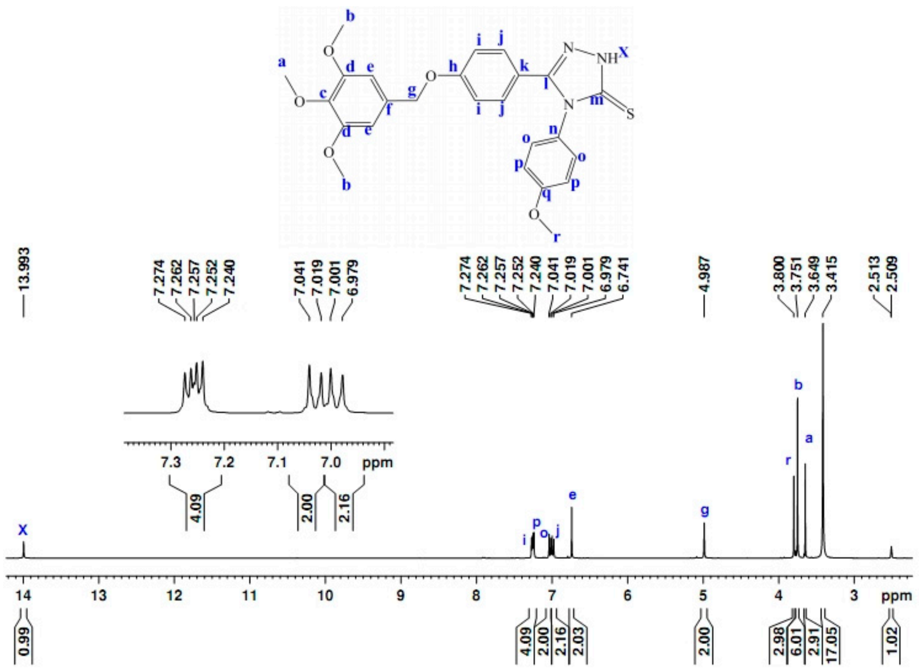

Figure S15. ^{1}H spectrum (DMSO-d_6, 400 MHz) of **4b.**

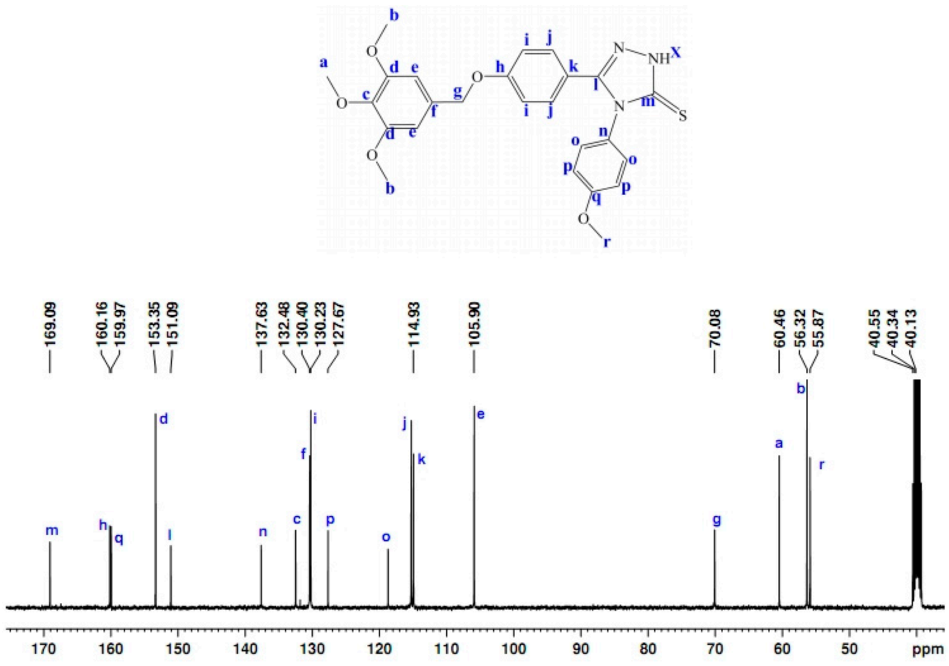

Figure S16. ^{13}C spectrum (DMSO-d_6, 100 MHz) of **4b.**

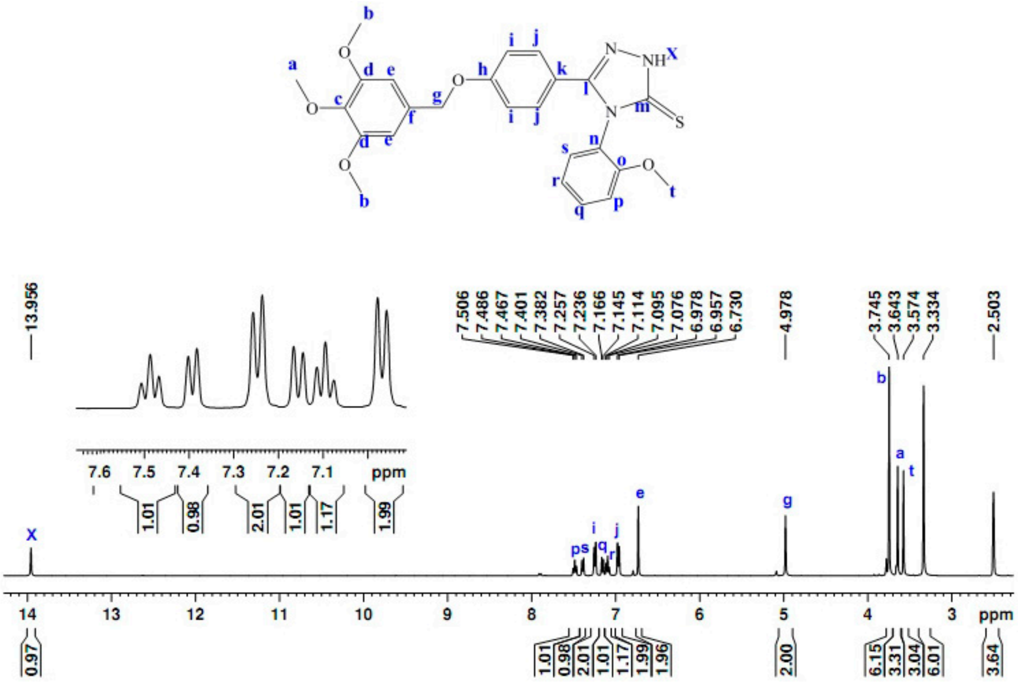

Figure S17. ^{1}H spectrum (DMSO-*d*$_6$, 400 MHz) of **4c.**

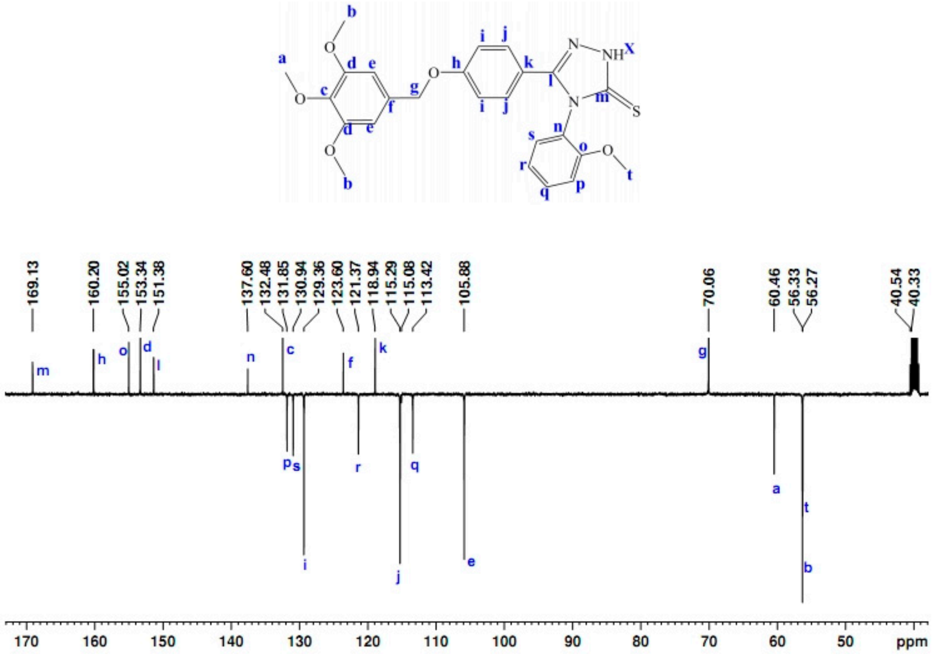

Figure S18. ^{13}C spectrum (DMSO-*d*$_6$, 100 MHz) of **4c.**

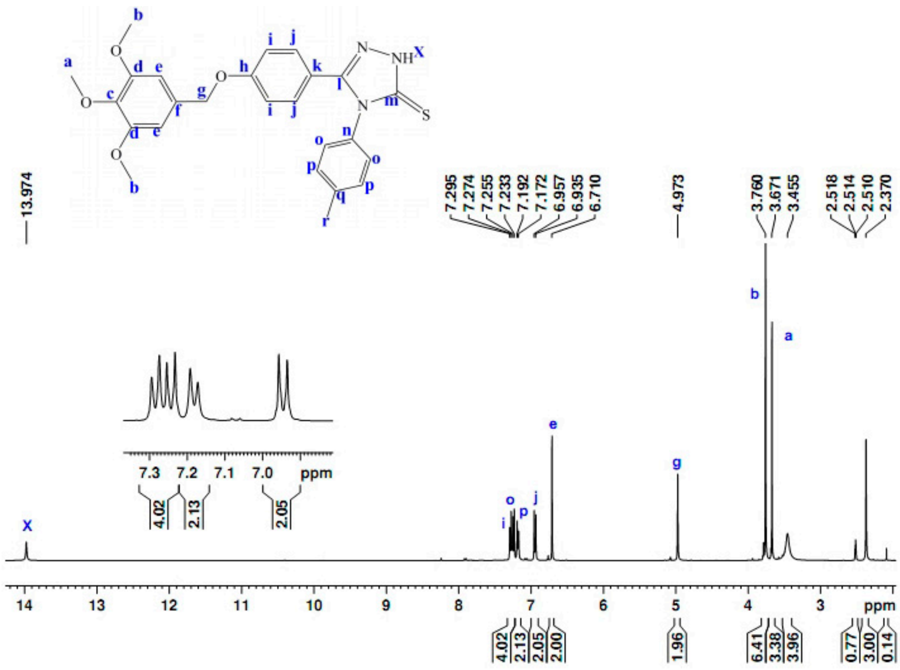

Figure S19. ^{1}H spectrum (DMSO-d_6, 400 MHz) of **4d.**

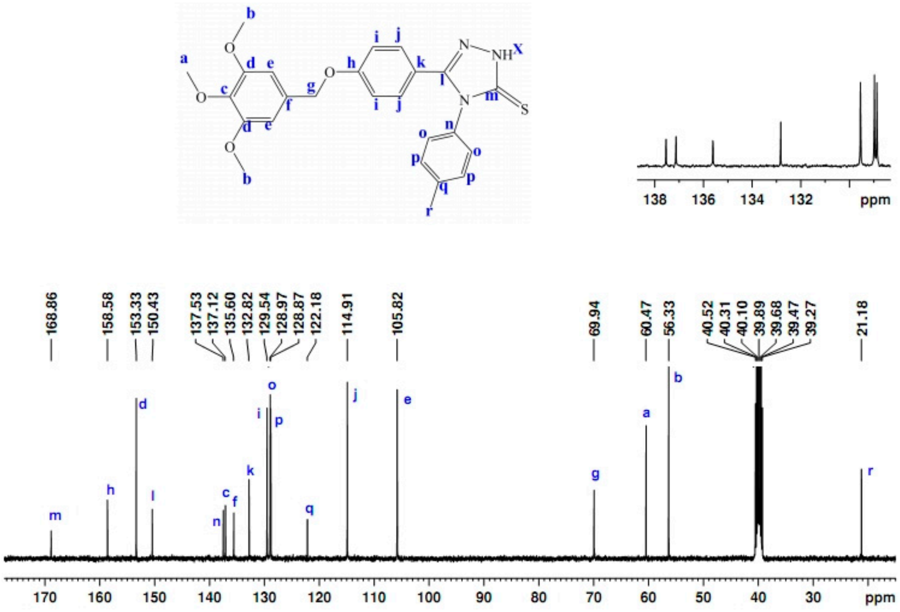

Figure S20. ^{13}C spectrum (DMSO-d_6, 100 MHz) of **4d.**

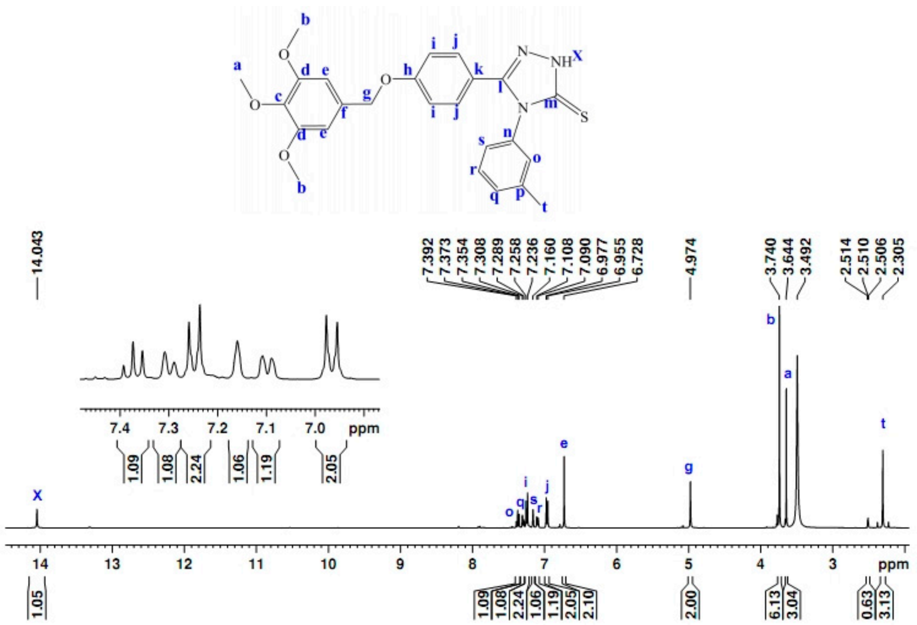

Figure S21. ^{1}H spectrum (DMSO-*d*6, 400 MHz) of **4e.**

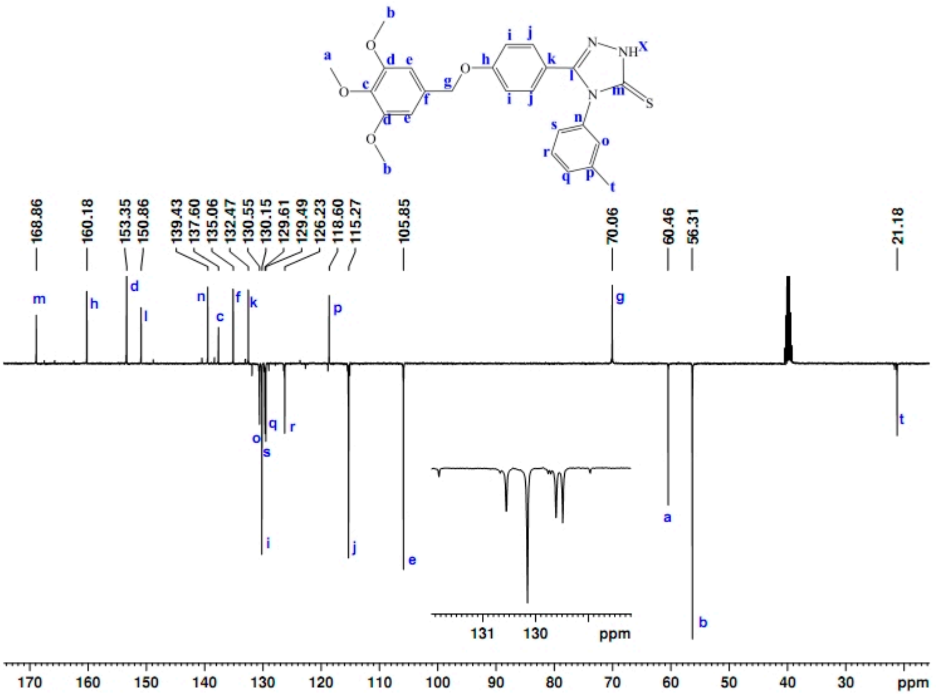

Figure S22. ^{13}C spectrum (DMSO-*d*6, 100 MHz) of **4e.**

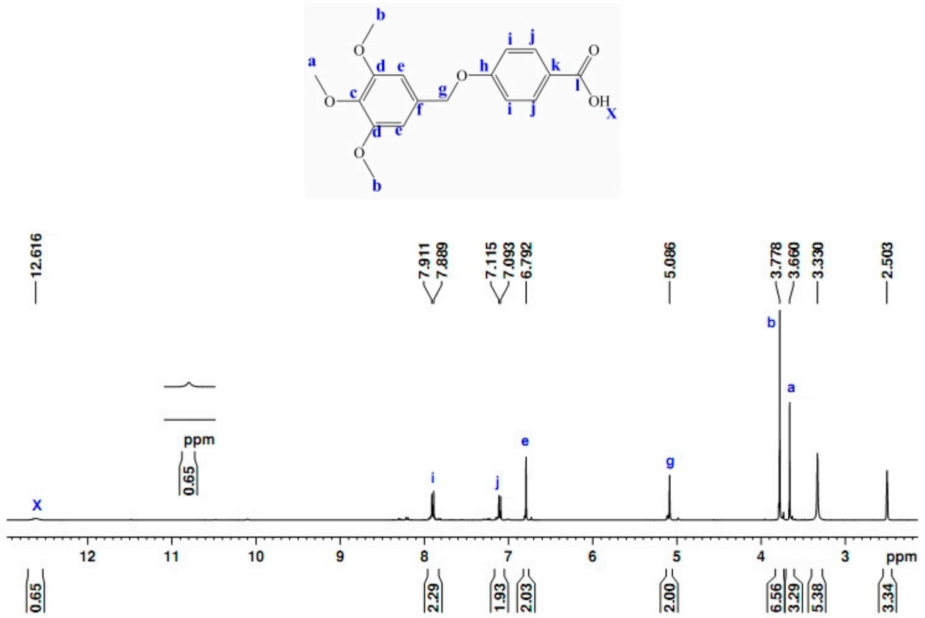

Figure S23. ^{1}H spectrum (DMSO-d_6, 400 MHz) of **4f.**

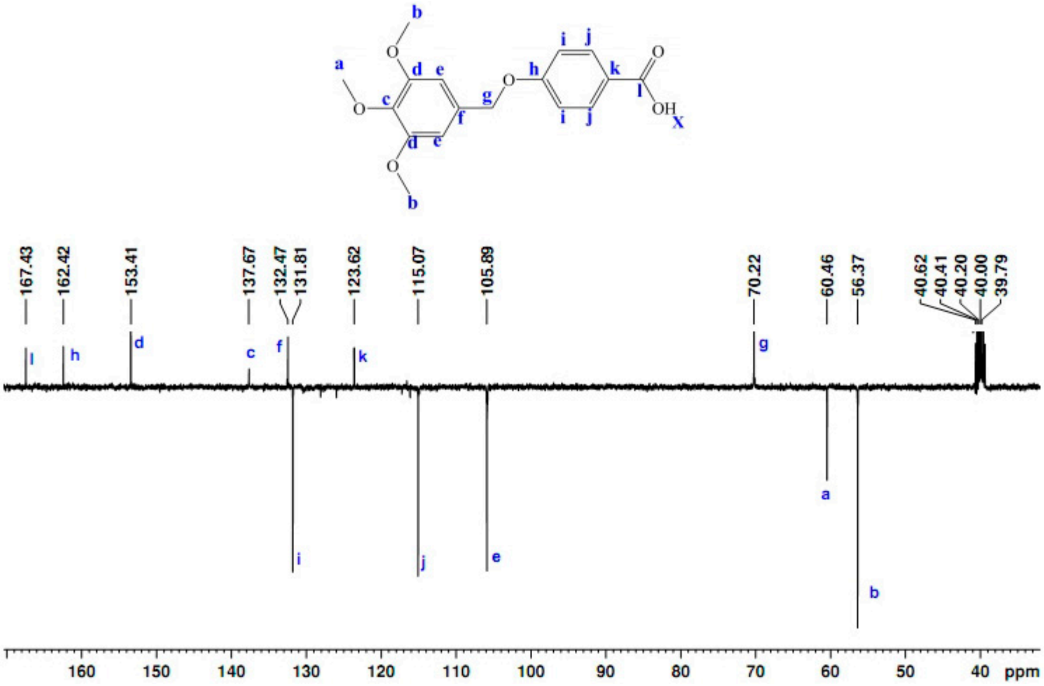

Figure S24. ^{13}C spectrum (DMSO-d_6, 100 MHz) of **4f.**

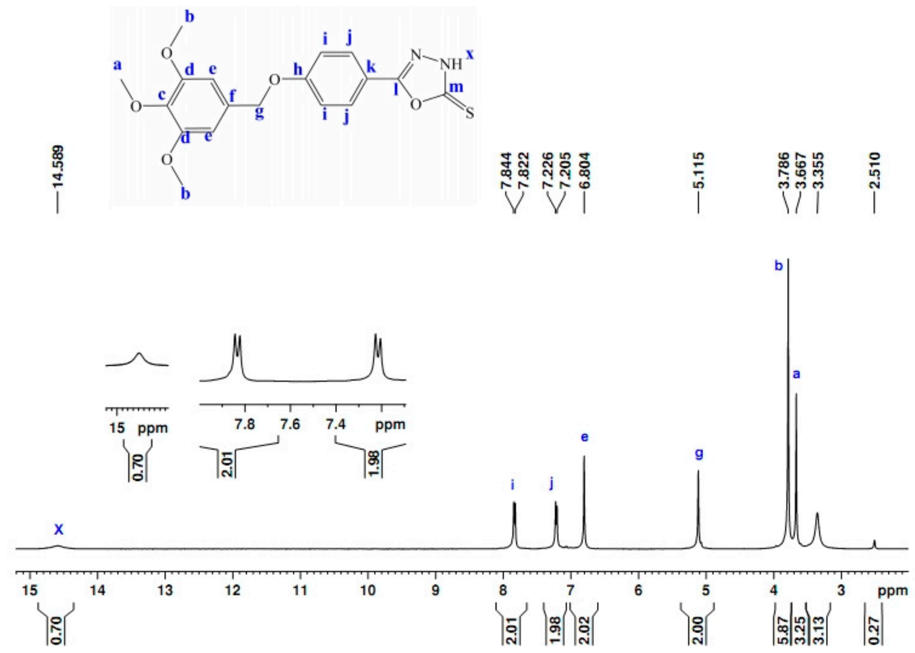

Figure S25. ^{1}H spectrum (DMSO-d_6, 400 MHz) of **5.**

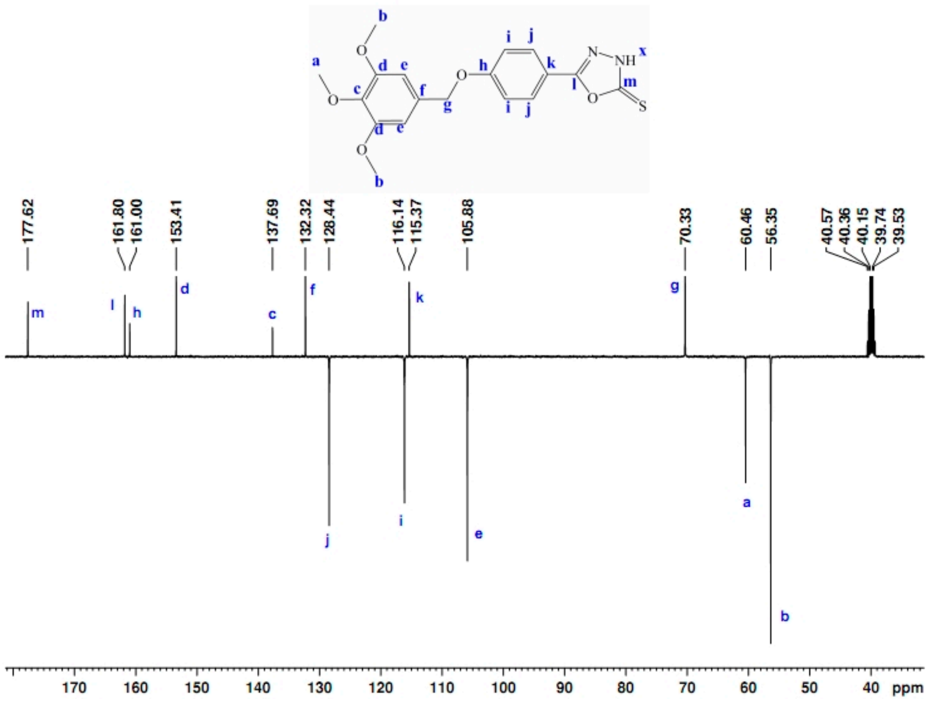

Figure S26. ^{13}C spectrum (DMSO-d_6, 100 MHz) of **5.**

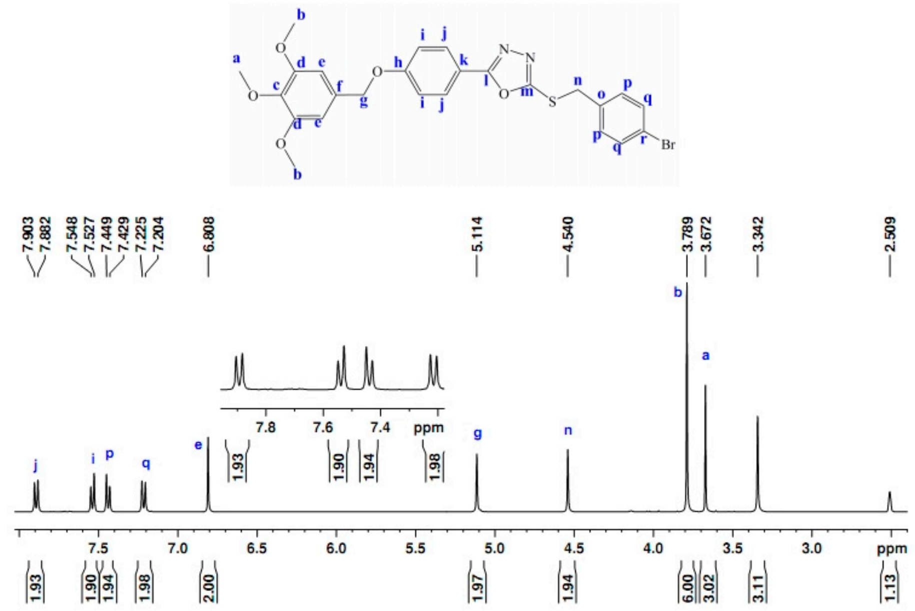

Figure S27. ^{1}H spectrum (DMSO-*d*6, 400 MHz) of **6a.**

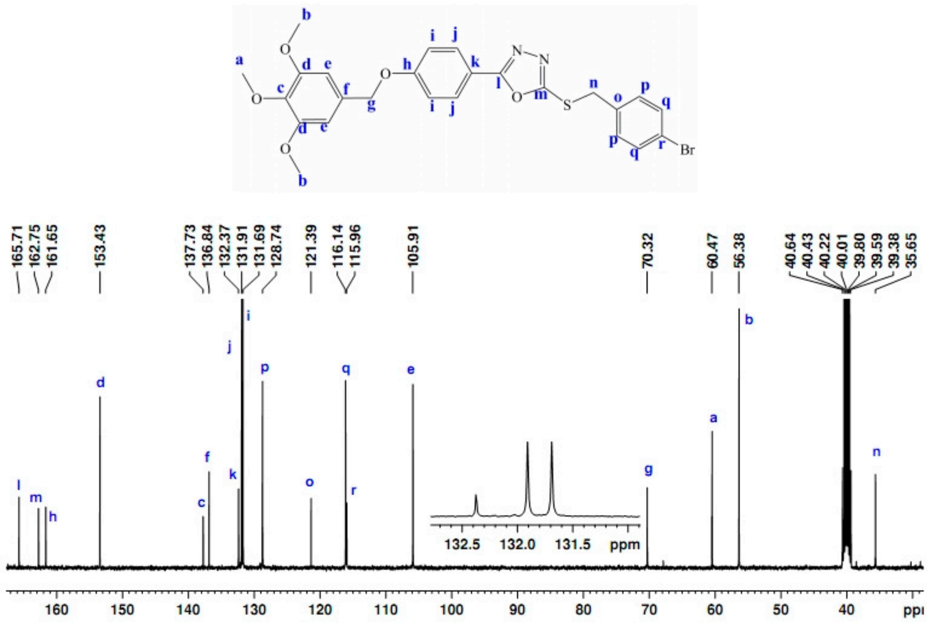

Figure S28. ^{13}C spectrum (DMSO-*d*6, 100 MHz) of **6a.**

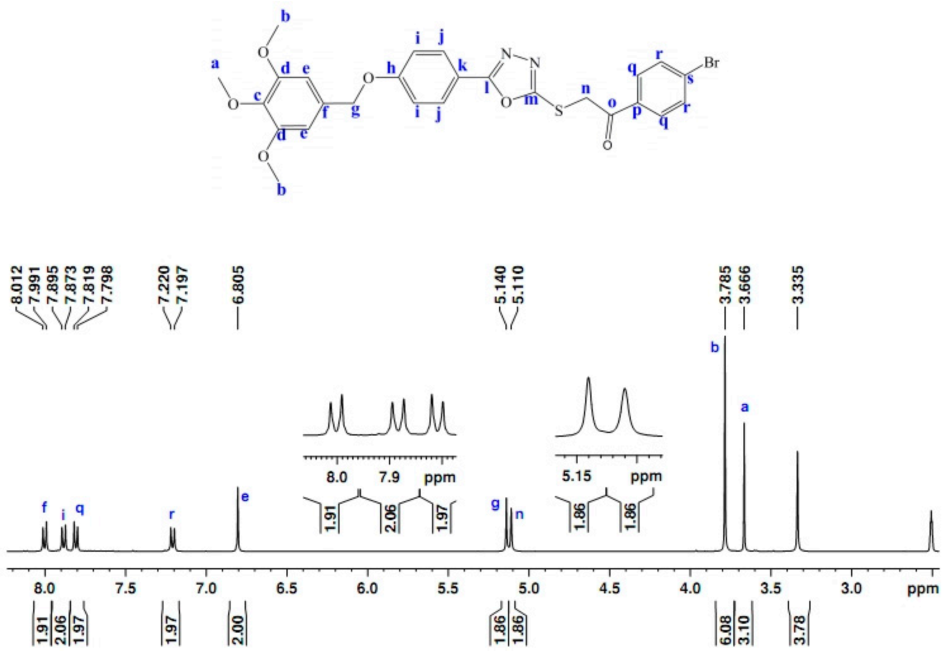

Figure S29. ${}^{1}H$ spectrum (DMSO-d_6, 400 MHz) of **6b.**

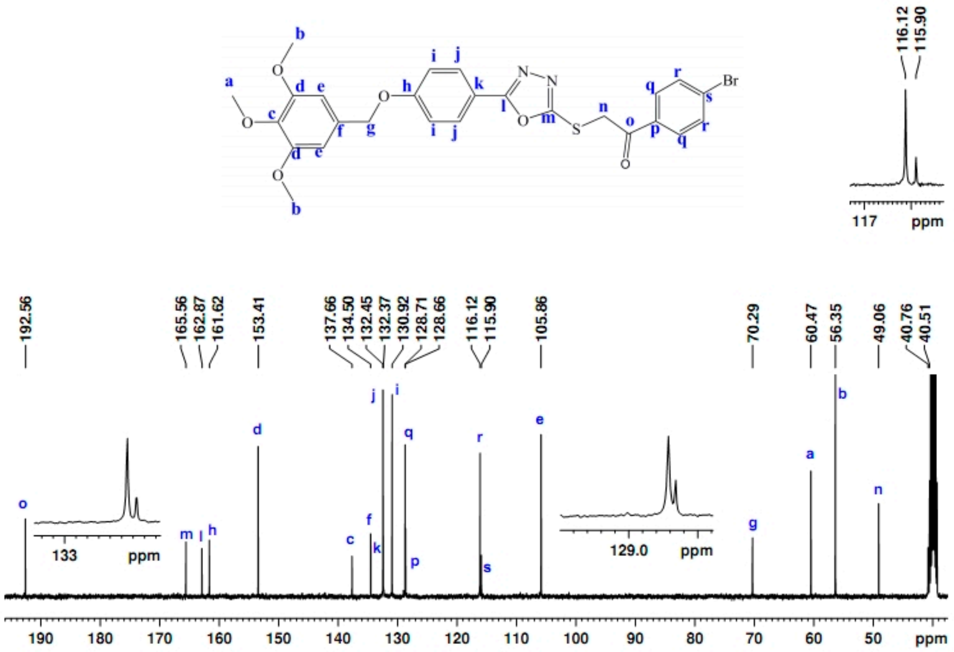

Figure S30. ${}^{13}C$ spectrum (DMSO-d_6, 100 MHz) of **6b.**

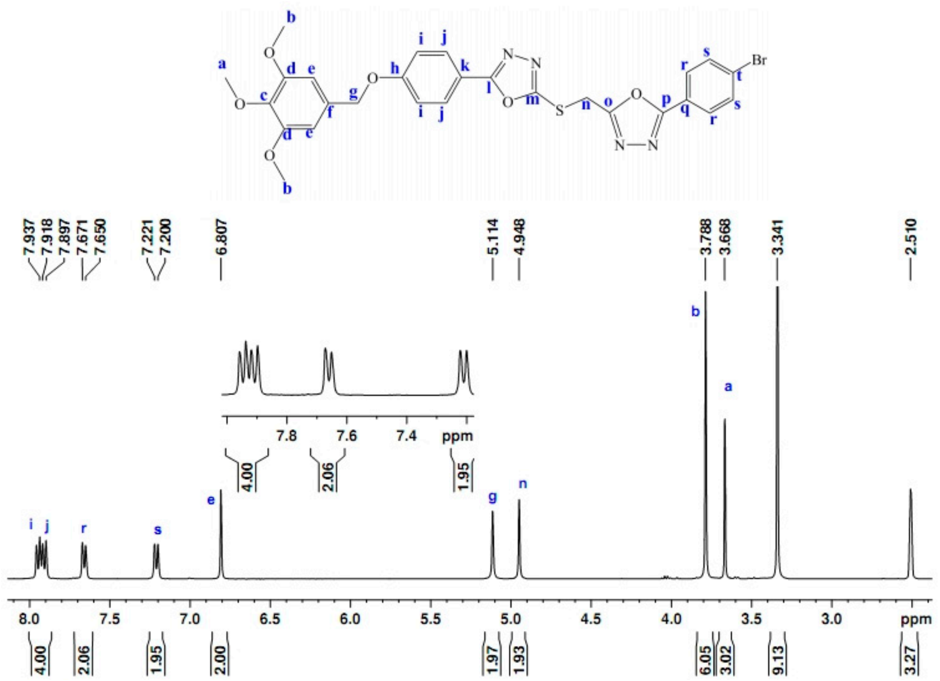

Figure S31. ^{1}H spectrum (DMSO-d_6, 400 MHz) of **6c.**

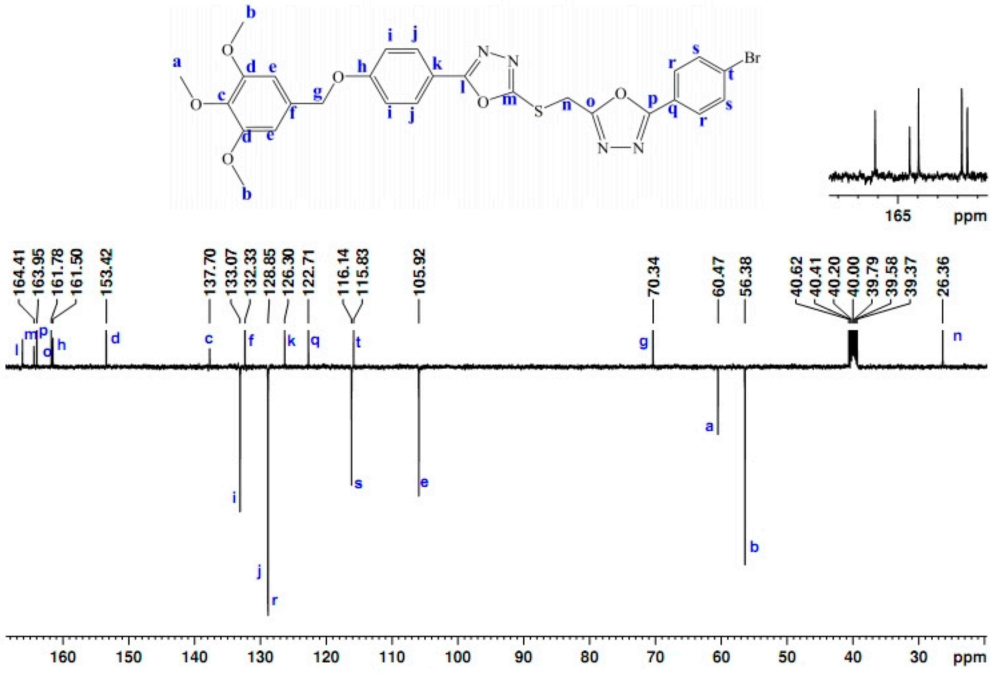

Figure S32. ^{13}C spectrum (DMSO-d_6, 100 MHz) of **6c.**

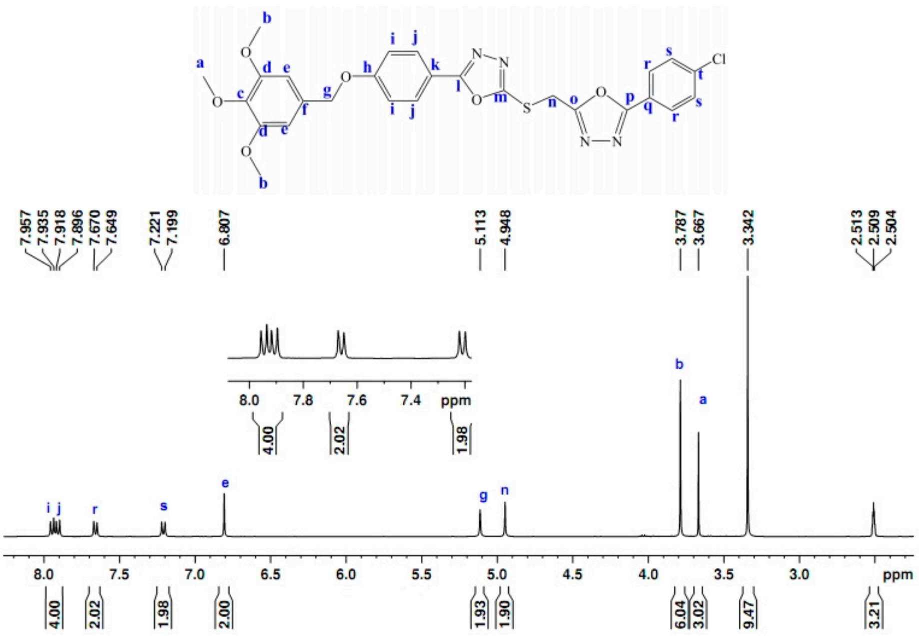

Figure S33. ^{1}H spectrum (DMSO-d_6, 400 MHz) of **6d.**

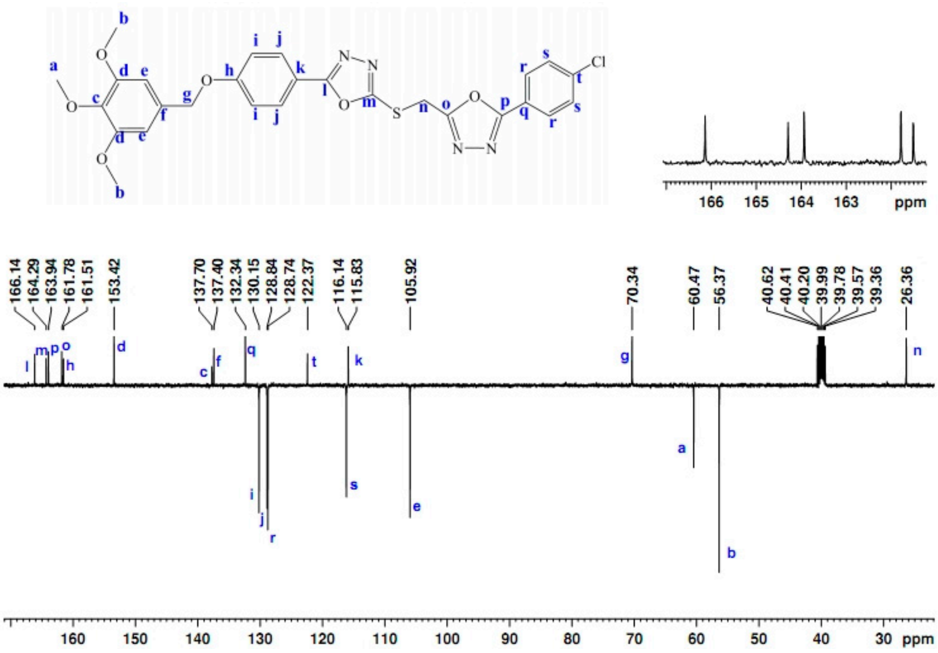

Figure S34. ^{13}C spectrum (DMSO-d_6, 100 MHz) of **6d.**

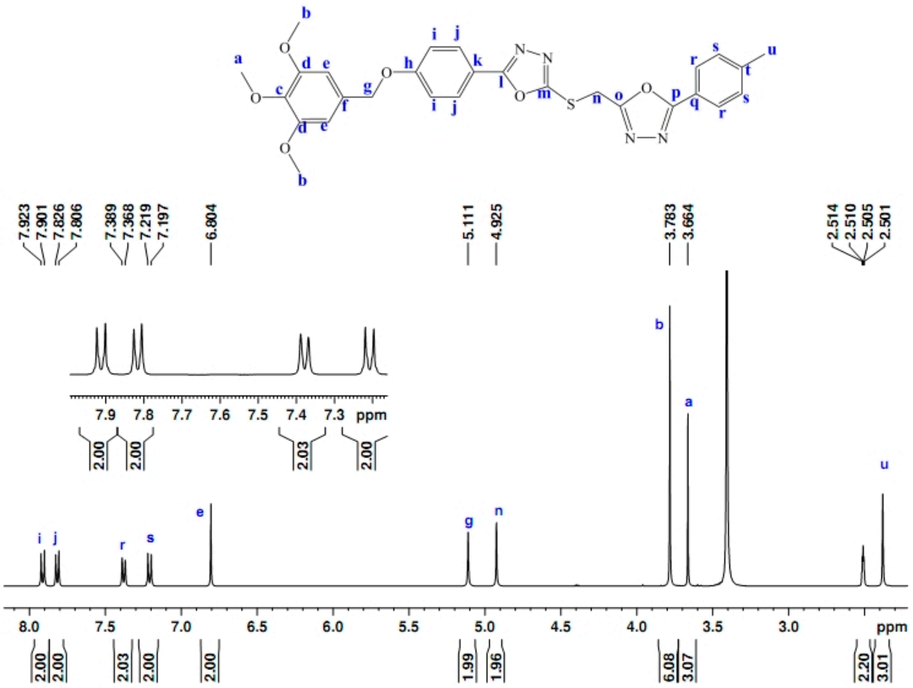

Figure S35. ^{1}H spectrum (DMSO-d_6, 400 MHz) of **6e.**

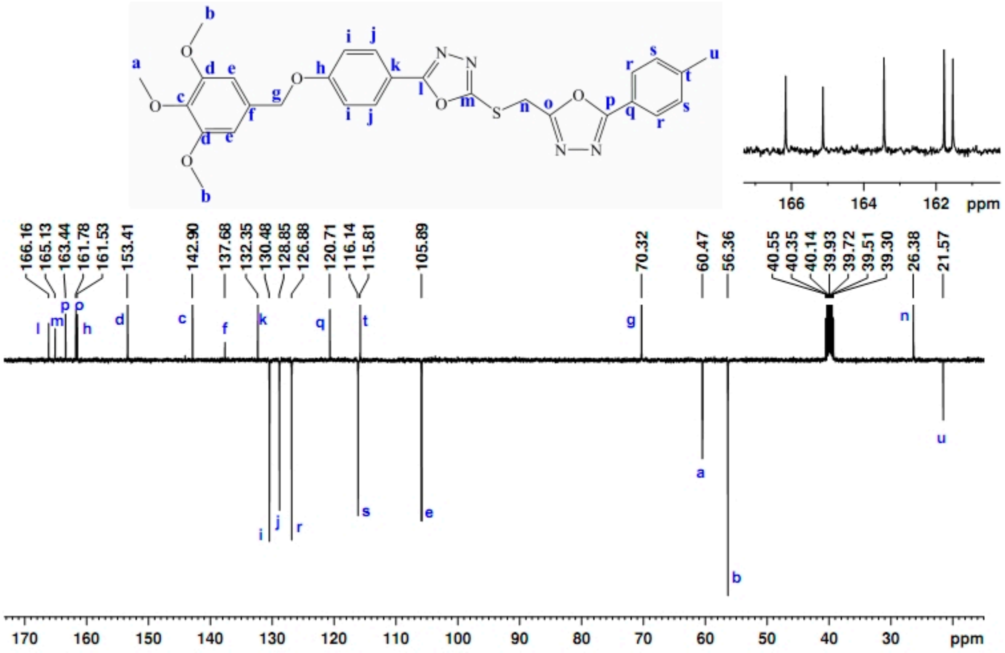

Figure S36. ^{13}C spectrum (DMSO-d_6, 100 MHz) of **6e.**

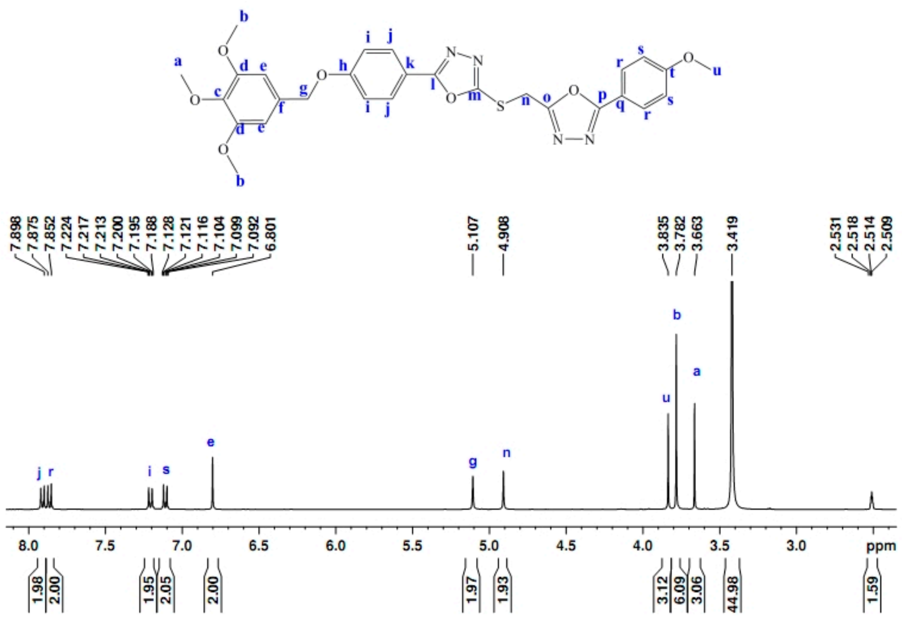

Figure S37. ^{1}H spectrum (DMSO-d_6, 400 MHz) of **6f.**

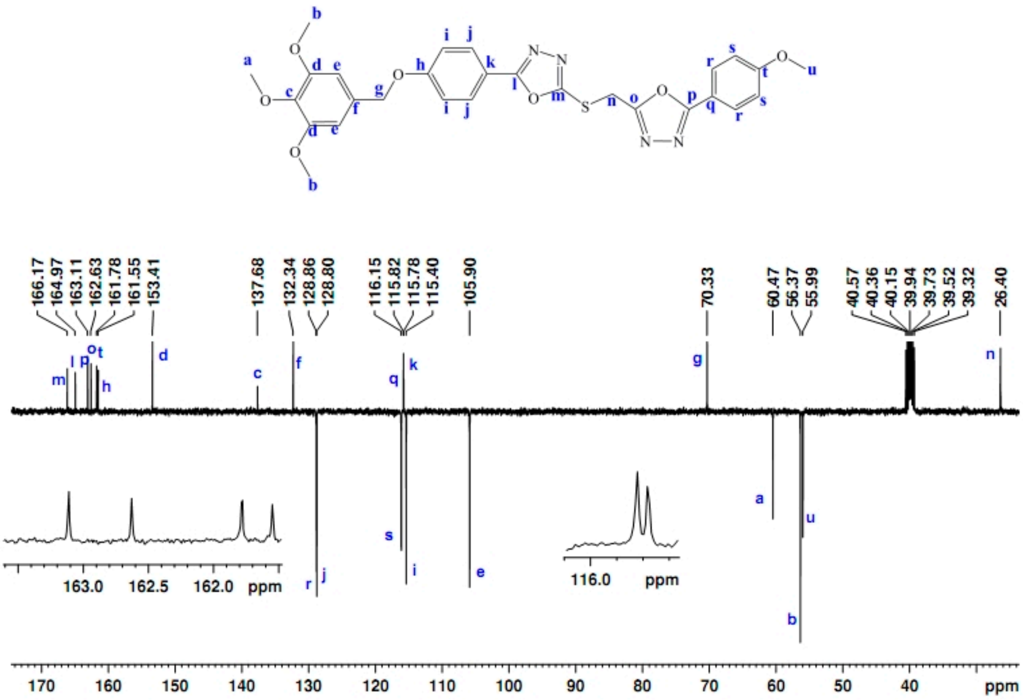

Figure S38. ^{13}C spectrum (DMSO-d_6, 100 MHz) of **6f.**

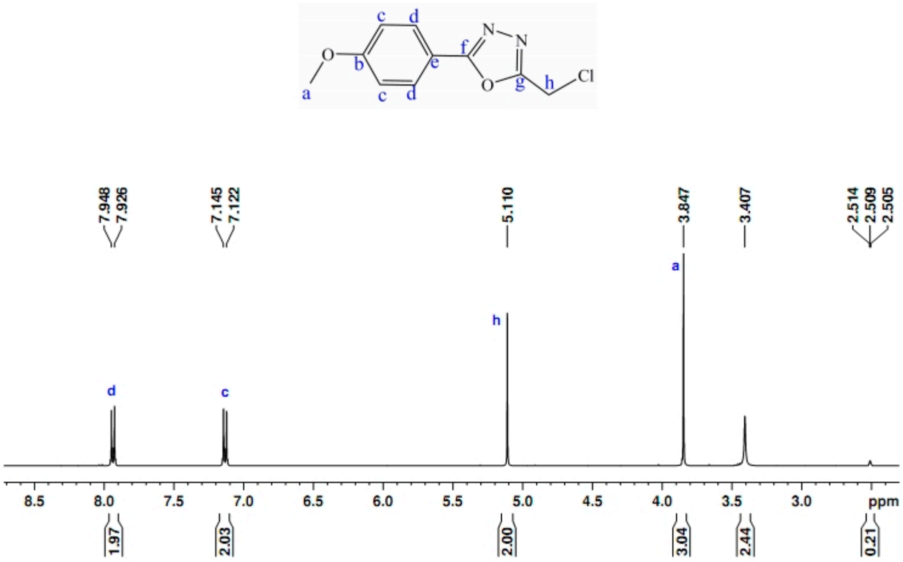

Figure S39. ^{1}H spectrum (DMSO-d_6, 400 MHz) of (**R′f**).

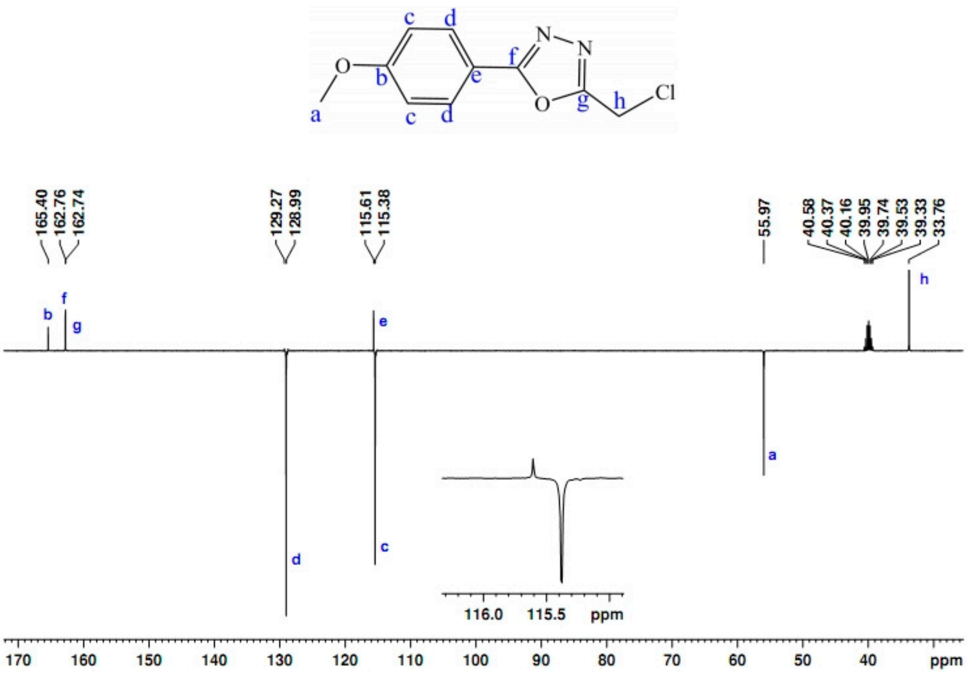

Figure S40. ^{13}C spectrum (DMSO-d_6, 100 MHz) of (**R′f**).

Computational Studies at Level of Theory B3LYP/6-311G*(d,p)

Figure S41. Compound 6a

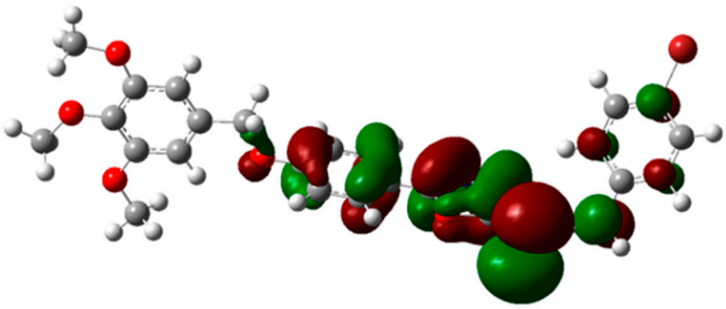

6a

Zero-point correction=	0.451221 (Hartree/Particle)
Thermal correction to Energy=	0.568231
Thermal correction to Enthalpy=	0.569124
Thermal correction to Gibbs Free Energy=	0.562234
Sum of electronic and zero-point Energies=	−4135.975609
Sum of electronic and thermal Energies=	−4135.978015
Sum of electronic and thermal Enthalpies=	−4135.954114
Sum of electronic and thermal Free Energies=	−4135.976213

	E (Thermal) KCal/Mol	CV Cal/Mol-Kelvin	S Cal/Mol-Kelvin
Total	298.413	118.120	225.451

Figure S42. Compound 6a-r1

6a-r1

Zero-point correction=	0.435813 (Hartree/Particle)
Thermal correction to Energy=	0.468444
Thermal correction to Enthalpy=	0.469388
Thermal correction to Gibbs Free Energy=	0.362148
Sum of electronic and zero-point Energies=	−4135.421677
Sum of electronic and thermal Energies=	−4135.389047
Sum of electronic and thermal Enthalpies=	−4135.388103
Sum of electronic and thermal Free Energies=	−4135.495343

	E (Thermal)	CV	S
	KCal/Mol	Cal/Mol-Kelvin	Cal/Mol-Kelvin
Total	293.953	119.790	225.706

Figure S43. Compound 6a-r2

6a-r2

Zero-point correction=	0.435309 (Hartree/Particle)
Thermal correction to Energy=	0.467942
Thermal correction to Enthalpy=	0.468887
Thermal correction to Gibbs Free Energy=	0.363327
Sum of electronic and zero-point Energies=	−4135.420798
Sum of electronic and thermal Energies=	−4135.388164
Sum of electronic and thermal Enthalpies=	−4135.387220
Sum of electronic and thermal Free Energies=	−4135.492779

	E (Thermal)	CV	S
	KCal/Mol	Cal/Mol-Kelvin	Cal/Mol-Kelvin
Total	293.638	120.466	222.169

Figure S44. Compound 6b

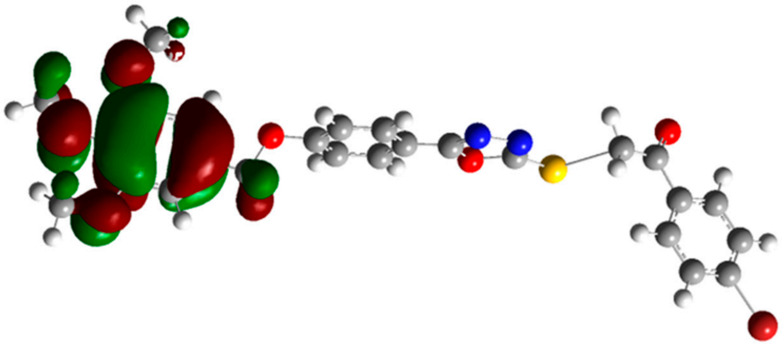

6b

Zero-point correction=	0.482112 (Hartree/Particle)
Thermal correction to Energy=	0.501234
Thermal correction to Enthalpy=	0.503026
Thermal correction to Gibbs Free Energy=	0.403978
Sum of electronic and zero-point Energies=	−4420.897388

Sum of electronic and thermal Energies= −4420.876376
Sum of electronic and thermal Enthalpies= −4420.827331
Sum of electronic and thermal Free Energies= −4420.873271

	E (Thermal)	CV	S
	KCal/Mol	Cal/Mol-Kelvin	Cal/Mol-Kelvin
Total	289.137	119.411	224.432

Figure S45. Compound 6b-r1

6b-r1

Zero-point correction= 0.426512 (Hartree/Particle)
Thermal correction to Energy= 0.459224
Thermal correction to Enthalpy= 0.460168
Thermal correction to Gibbs Free Energy= 0.353528
Sum of electronic and zero-point Energies= −4420.282688
Sum of electronic and thermal Energies= −4420.249976
Sum of electronic and thermal Enthalpies= −4420.249031
Sum of electronic and thermal Free Energies= −4420.355671

	E (Thermal)	CV	S
	KCal/Mol	Cal/Mol-Kelvin	Cal/Mol-Kelvin
Total	288.167	119.456	224.443

Figure S46. Compound 6b-r2

6b-r2

Zero-point correction= 0.426439 (Hartree/Particle)
Thermal correction to Energy= 0.459121
Thermal correction to Enthalpy= 0.460065
Thermal correction to Gibbs Free Energy= 0.353179
Sum of electronic and zero-point Energies= −4420.291536
Sum of electronic and thermal Energies= −4420.258855
Sum of electronic and thermal Enthalpies= −4420.257910
Sum of electronic and thermal Free Energies= −4420.364797

	E (Thermal)	CV	S
	KCal/Mol	Cal/Mol-Kelvin	Cal/Mol-Kelvin
Total	288.103	119.679	224.961

Figure S47. Compound 6e

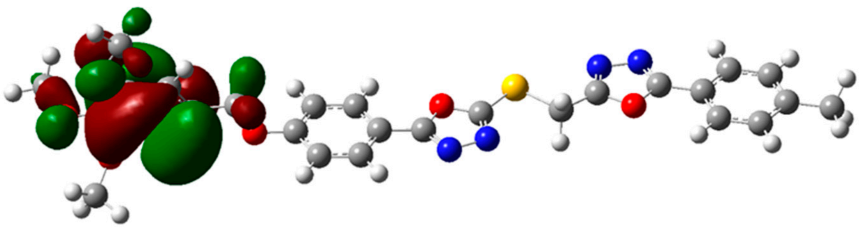

6e

Zero-point correction= 0.502455 (Hartree/Particle)

Thermal correction to Energy= 0.539401
Thermal correction to Enthalpy= 0.540345
Thermal correction to Gibbs Free Energy= 0.422612
Sum of electronic and zero-point Energies= −2150.614196
Sum of electronic and thermal Energies= −2150.577251
Sum of electronic and thermal Enthalpies= −2150.576306
Sum of electronic and thermal Free Energies= −2150.694039

	E (Thermal)	CV	S
	KCal/Mol	Cal/Mol-Kelvin	Cal/Mol-Kelvin
Total	338.479	134.573	247.789

Figure S48. Compound 6e-r1

6e-r1

Zero-point correction= 0.488769 (Hartree/Particle)
Thermal correction to Energy= 0.525582
Thermal correction to Enthalpy= 0.526526
Thermal correction to Gibbs Free Energy= 0.409200
Sum of electronic and zero-point Energies= −2149.986588
Sum of electronic and thermal Energies= −2149.949775
Sum of electronic and thermal Enthalpies= −2149.948831
Sum of electronic and thermal Free Energies= −2150.066157

	E (Thermal)	CV	S
	KCal/Mol	Cal/Mol-Kelvin	Cal/Mol-Kelvin
Total	329.807	134.908	246.934

Figure S49. Compound 6f

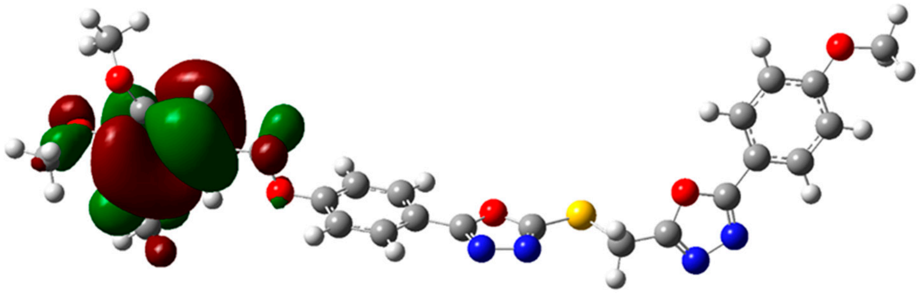

6f

Zero-point correction= 0.508304 (Hartree/Particle)
Thermal correction to Energy= 0.545626
Thermal correction to Enthalpy= 0.546571
Thermal correction to Gibbs Free Energy= 0.429944
Sum of electronic and zero-point Energies= −2225.816610
Sum of electronic and thermal Energies= −2225.779288
Sum of electronic and thermal Enthalpies= −2225.778343
Sum of electronic and thermal Free Energies= −2225.894971

	E (Thermal)	CV	S
	KCal/Mol	Cal/Mol-Kelvin	Cal/Mol-Kelvin
Total	342.386	137.567	245.462

Figure S50. Compound 6f-r1

6f-r1

Zero-point correction=	0.454447 (Hartree/Particle)
Thermal correction to Energy=	0.531747
Thermal correction to Enthalpy=	0.532692
Thermal correction to Gibbs Free Energy=	0.415957
Sum of electronic and zero−point Energies=	−2225.188388
Sum of electronic and thermal Energies=	−2225.151088
Sum of electronic and thermal Enthalpies=	−2225.150144
Sum of electronic and thermal Free Energies=	−2225.266879

	E (Thermal)	CV	S
	KCal/Mol	Cal/Mol-Kelvin	Cal/Mol-Kelvin
Total	333.677	137.929	245.689

Figure S51. Single point energy calculation for compounds 3f, 6a and 6b

3f.out

Title Card Required

Charge = 0 Multiplicity = 1
Symbolic Z-Matrix:

C	7.7444	−0.50102	1.11674
C	6.54698	0.20809	1.05124
C	6.04703	0.63169	−0.18615
C	6.75079	0.35427	−1.35067
C	7.95394	−0.35573	−1.29519
C	8.45885	−0.78832	−0.06618
H	5.98834	0.42887	1.95086
H	6.38127	0.66	−2.32219
O	8.58347	−0.66552	−2.47215
O	9.6565	−1.45215	−0.01306
O	8.31071	−0.96594	2.26399
C	9.55309	−2.87257	0.1592
H	10.5744	−3.2522	0.1616
H	9.07213	−3.11498	1.10994
H	8.9973	−3.32178	−0.66962
C	7.6626	−0.68805	3.49878
H	6.66505	−1.13877	3.54003
H	8.29126	−1.13176	4.26866
H	7.58339	0.39028	3.67348
C	9.86521	−0.05567	−2.68225
H	10.5767	−0.3567	−1.9119
H	10.20453	−0.40251	−3.65779
H	9.77193	1.03593	−2.69871
C	4.74443	1.37956	−0.24263
H	4.70832	2.1596	0.52627
H	4.61071	1.84742	−1.22277
O	3.67343	0.44209	−0.00732
C	2.39892	0.89205	0.02221

C	1.41661	−0.08134	0.26469
C	2.01458	2.22783	−0.16312
C	0.08099	0.27054	0.31301
H	1.73461	−1.10497	0.41711
C	0.67081	2.56957	−0.10328
H	2.74843	2.99878	−0.35199
C	−0.31781	1.60615	0.12327
H	−0.64208	−0.50739	0.52994
H	0.36282	3.59937	−0.23453
C	−1.72248	2.08134	0.17843
O	−2.05115	3.23971	0.28627
N	−2.69647	1.05479	0.0675
H	−2.44956	0.08586	−0.16674
N	−3.97303	1.324	0.16775
C	−4.81852	0.25074	−0.02432
N	−6.10214	0.71655	0.12098
H	−6.10337	1.70865	0.33161
S	−4.31922	−1.31867	−0.36867
C	−7.36069	0.12806	0.04103
C	−8.45809	0.98014	0.28704
C	−7.59654	−1.22328	−0.26447
C	−9.75273	0.50315	0.23224
H	−8.28205	2.02417	0.52308
C	−8.89888	−1.69928	−0.31896
H	−6.76798	−1.88518	−0.45625
C	−9.96284	−0.84036	−0.07167
H	−10.60075	1.14646	0.41936
H	−9.09961	−2.73547	−0.55293
N	−11.33983	−1.35586	−0.13109
O	−12.25056	−0.56658	0.0906
O	−11.49264	−2.54119	−0.39804
H	−4.31197	2.24295	0.36938

Stoichiometry C24H24N4O7S
Framework group C1[X(C24H24N4O7S)]
Deg. of freedom 174
Full point group C1 NOp 1
Largest Abelian subgroup C1 NOp 1
Largest concise Abelian subgroup C1 NOp 1

Standard orientation:

Center Number	Atomic Number	Atomic Type	Coordinates (Angstroms) X	Y	Z
1	6	0	7.760733	−0.499470	1.118178
2	6	0	6.562794	0.208513	1.049858
3	6	0	6.062686	0.627064	−0.189183
4	6	0	6.766786	0.345753	−1.352557
5	6	0	7.970448	−0.363147	−1.294259
6	6	0	8.475529	−0.790718	−0.063558
7	1	0	6.003885	0.432276	1.948572
8	1	0	6.397154	0.647537	−2.325267
9	8	0	8.600348	−0.676922	−2.469960

10	8	0	9.673664	−1.453455	−0.007795
11	8	0	8.327239	−0.959633	2.267252
12	6	0	9.571273	−2.873288	0.169829
13	1	0	10.592864	−3.252161	0.173782
14	1	0	9.090383	−3.112457	1.121419
15	1	0	9.015907	−3.326041	−0.657349
16	6	0	7.678782	−0.677547	3.500897
17	1	0	6.681562	−1.128840	3.543735
18	1	0	8.307680	−1.117881	4.272524
19	1	0	7.598763	0.401378	3.671516
20	6	0	9.881662	−0.066928	−2.682218
21	1	0	10.593284	−0.364522	−1.910654
22	1	0	10.221351	−0.417202	−3.656398
23	1	0	9.787581	1.024533	−2.702814
24	6	0	4.759541	1.373758	−0.248642
25	1	0	4.722768	2.156671	0.517299
26	1	0	4.625592	1.837811	−1.230552
27	8	0	3.689201	0.436397	−0.009914
28	6	0	2.414358	0.885523	0.017772
29	6	0	1.432734	−0.087660	0.263810
30	6	0	2.029055	2.220315	−0.172652
31	6	0	0.096854	0.263413	0.310651
32	1	0	1.751471	−1.110474	0.420137
33	6	0	0.685034	2.561287	−0.114259
34	1	0	2.762369	2.991080	−0.364355
35	6	0	−0.302912	1.598003	0.115809
36	1	0	−0.625674	−0.514218	0.530431
37	1	0	0.376299	3.590355	−0.249441
38	6	0	−1.707933	2.072369	0.169015
39	8	0	−2.037470	3.230901	0.272437
40	7	0	−2.681153	1.044692	0.061852
41	1	0	−2.433503	0.075071	−0.168688
42	7	0	−3.957923	1.313347	0.160936
43	6	0	−4.802602	0.238747	−0.027172
44	7	0	−6.086579	0.704157	0.116218
45	1	0	−6.088569	1.697044	0.323092
46	16	0	−4.302114	−1.331591	−0.365526
47	6	0	−7.344691	0.114440	0.038341
48	6	0	−8.442747	0.966645	0.281002
49	6	0	−7.579514	−1.238210	−0.262068
50	6	0	−9.737033	0.488494	0.227860
51	1	0	−8.267495	2.011687	0.513111
52	6	0	−8.881496	−1.715367	−0.314911
53	1	0	−6.750439	−1.900224	−0.451247
54	6	0	−9.946115	−0.856308	−0.070991
55	1	0	−10.585548	1.131887	0.412448
56	1	0	−9.081441	−2.752582	−0.544982
57	7	0	−11.322717	−1.373040	−0.128622
58	8	0	−12.234055	−0.583592	0.089970
59	8	0	−11.474626	−2.559477	−0.391110
60	1	0	−4.297568	2.232798	0.359052

Rotational constants (GHZ): 0.3166523 0.0189429 0.0186441
Standard basis: 6-311++G(d,p) (5D, 7F)
There are 968 symmetry adapted basis functions of A symmetry.
Integral buffers will be 131072 words long.
Raffenetti 2 integral format.
Two-electron integral symmetry is turned on.
968 basis functions, 1526 primitive gaussians, 1004 cartesian basis functions
134 alpha electrons 134 beta electrons
nuclear repulsion energy 3474.9084117772 Hartrees.
NAtoms= 60 NActive= 60 NUniq= 60 SFac= 1.00D+00 NAtFMM= 60 NAOKFM=T Big=T

Polarizable Continuum Model (PCM)
=====================================
Model : PCM.
Atomic radii : UFF (Universal Force Field).
Polarization charges : Total charges.
Charge compensation : None.
Solution method : Matrix inversion.
Cavity type : Scaled VdW (van der Waals Surface) (Alpha=1.100).
Cavity algorithm : GePol (No added spheres)
Default sphere list used, NSphG= 60.
Lebedev-Laikov grids with approx. 5.0 points / Ang**2.
Smoothing algorithm: Karplus/York (Gamma=1.0000).
Polarization charges: spherical gaussians, with
point-specific exponents (IZeta= 3).
Self-potential: point-specific (ISelfS= 7).
Self-field : sphere-specific E.n sum rule (ISelfD= 2).
Solvent : DiMethylSulfoxide, Eps= 46.826000 Eps(inf)= 2.007889

3f-r1.out

Title Card Required

Charge = 0 Multiplicity = 2
Symbolic Z-Matrix:

C	−3.32402	0.08398	0.81162
C	−1.92546	0.04121	0.73479
C	−1.2326	1.02669	0.01881
C	−1.93824	2.05547	−0.61969
C	−3.33687	2.09842	−0.54264
C	−4.0298	1.11235	0.17268
H	−1.38664	−0.74396	1.22271
H	−1.40911	2.80815	−1.16602
O	−4.05677	3.14846	−1.19392
O	−5.45699	1.1553	0.25081
O	−4.03107	−0.92174	1.54208
C	−5.88421	0.57436	1.4857
H	−6.9521	0.60646	1.54439
H	−5.55472	−0.44251	1.53432
H	−5.46543	1.12439	2.30242
C	−3.29636	−2.1478	1.49881

H	−2.33134	−2.00103	1.93713
H	−3.82565	−2.90017	2.04532
H	−3.18236	−2.45962	0.48161
C	−4.38068	2.74826	−2.52804
H	−4.98588	1.86636	−2.49891
H	−4.91886	3.53421	−3.01546
H	−3.47962	2.54481	−3.06803
C	0.30423	0.97898	−0.06631
H	0.69651	0.52443	0.81936
H	0.59642	0.40543	−0.92103
O	0.81676	2.30846	−0.18687
C	2.24374	2.263	−0.26692
C	3.01362	2.32531	0.90238
C	2.87228	2.15599	−1.51487
C	4.41213	2.28055	0.82373
H	2.53369	2.40704	1.85516
C	4.27071	2.11088	−1.59351
H	2.28438	2.10866	−2.40768
C	5.04065	2.17327	−0.42424
H	4.99993	2.32822	1.71645
H	4.75057	2.02887	−2.54631
C	6.57742	2.12398	−0.51106
O	7.1412	2.0255	−1.63176
N	7.38602	2.19206	0.71471
H	7.5956	3.14743	0.92291
N	8.57838	1.48288	0.52682
C	9.38963	1.55437	1.75067
N	9.23557	0.53295	2.7966
S	10.41909	2.71969	1.94019
C	8.25993	0.72952	3.87846
C	6.94554	0.26669	3.72993
C	8.64458	1.3799	5.05871
C	6.01588	0.45425	4.76178
H	6.65181	−0.22989	2.82881
C	7.71493	1.56757	6.09036
H	9.64816	1.73323	5.17211
C	6.40061	1.10468	5.94196
H	5.01231	0.10079	4.64855
H	8.0086	2.06434	6.9914
N	5.42552	1.30151	7.02429
O	4.26475	0.89271	6.89341
O	5.76531	1.87604	8.06648
H	8.36869	0.527	0.3211

Stoichiometry C24H23N4O7S(2)
Framework group C1[X(C24H23N4O7S)]
Deg. of freedom 171
Full point group C1 NOp 1
Largest Abelian subgroup C1 NOp 1
Largest concise Abelian subgroup C1 NOp 1

Standard orientation:

Center Atomic Atomic Coordinates (Angstroms)

Number	Number	Type	X	Y	Z
1	6	0	−6.170152	1.153904	0.733948
2	6	0	−4.847448	0.747465	0.955179
3	6	0	−4.323420	−0.345650	0.252082
4	6	0	−5.121850	−1.031880	−0.672907
5	6	0	−6.444568	−0.625268	−0.894370
6	6	0	−6.968859	0.467407	−0.190555
7	1	0	−4.237656	1.271710	1.661018
8	1	0	−4.721537	−1.866272	−1.210031
9	8	0	−7.259077	−1.325365	−1.838529
10	8	0	−8.318762	0.882078	−0.415586
11	8	0	−6.704965	2.269246	1.451537
12	6	0	−8.427703	2.290603	−0.193855
13	1	0	−9.437705	2.601108	−0.362245
14	1	0	−8.148783	2.515556	0.814382
15	1	0	−7.778103	2.809087	−0.867748
16	6	0	−6.071361	2.361882	2.730154
17	1	0	−5.018019	2.495299	2.597465
18	1	0	−6.471765	3.196413	3.266926
19	1	0	−6.250208	1.463275	3.282816
20	6	0	−7.948660	−2.389205	−1.176965
21	1	0	−8.567393	−1.986111	−0.402647
22	1	0	−8.557671	−2.913328	−1.883596
23	1	0	−7.236880	−3.064228	−0.749648
24	6	0	−2.870153	−0.792729	0.496039
25	1	0	−2.278039	0.054534	0.772537
26	1	0	−2.847155	−1.516396	1.283858
27	8	0	−2.342614	−1.373210	−0.699576
28	6	0	−0.993562	−1.788989	−0.471700
29	6	0	0.063879	−0.902660	−0.716852
30	6	0	−0.729008	−3.082917	−0.003042
31	6	0	1.385951	−1.310271	−0.493277
32	1	0	−0.138150	0.085233	−1.074702
33	6	0	0.592974	−3.490408	0.220862
34	1	0	−1.536456	−3.759720	0.183895
35	6	0	1.650471	−2.604146	−0.024355
36	1	0	2.193304	−0.633623	−0.680540
37	1	0	0.794922	−4.478239	0.578976
38	6	0	3.103142	−3.052404	0.221516
39	8	0	3.339954	−4.213614	0.644601
40	7	0	4.213270	−2.124360	−0.037964
41	1	0	4.518751	−2.226773	−0.984644
42	7	0	5.274330	−2.412590	0.828647
43	6	0	6.386663	−1.487872	0.566635
44	7	0	6.467690	−0.210386	1.289450
45	16	0	7.486440	−1.864240	−0.483370
46	6	0	5.829157	0.991161	0.733101
47	6	0	4.501567	1.295966	1.062458
48	6	0	6.548481	1.832004	−0.126831
49	6	0	3.893405	2.441720	0.531857
50	1	0	3.952319	0.653991	1.719026

51	6	0	5.940280	2.977549	−0.657499
52	1	0	7.562137	1.599286	−0.378259
53	6	0	4.612784	3.282473	−0.328108
54	1	0	2.879792	2.674632	0.783365
55	1	0	6.489468	3.619408	−1.314239
56	7	0	3.974925	4.484248	−0.884753
57	8	0	2.802565	4.753711	−0.593899
58	8	0	4.610251	5.226643	−1.644303
59	1	0	4.969387	−2.307701	1.775227

Rotational constants (GHZ): 0.1428738 0.0335642 0.0285526
Standard basis: 6-311++G(d,p) (5D, 7F)
There are 961 symmetry adapted basis functions of A symmetry.
Integral buffers will be 131072 words long.
Raffenetti 2 integral format.
Two-electron integral symmetry is turned on.
961 basis functions, 1517 primitive gaussians, 997 cartesian basis functions
134 alpha electrons 133 beta electrons
nuclear repulsion energy 3616.4865002445 Hartrees.
NAtoms= 59 NActive= 59 NUniq= 59 SFac= 1.00D+00 NAtFMM= 60 NAOKFM=F Big=F

Polarizable Continuum Model (PCM)
==================================
Model : PCM.
Atomic radii : UFF (Universal Force Field).
Polarization charges : Total charges.
Charge compensation : None.
Solution method : Matrix inversion.
Cavity type : Scaled VdW (van der Waals Surface) (Alpha=1.100).
Cavity algorithm : GePol (No added spheres)
Default sphere list used, NSphG= 59.
Lebedev-Laikov grids with approx. 5.0 points / Ang**2.
Smoothing algorithm: Karplus/York (Gamma=1.0000).
Polarization charges: spherical gaussians, with
point-specific exponents (IZeta= 3).
Self-potential: point-specific (ISelfS= 7).
Self-field : sphere-specific E.n sum rule (ISelfD= 2).
Solvent : DiMethylSulfoxide, Eps= 46.826000 Eps(inf)= 2.007889

3f-r2.out

Title Card Required

Charge = 0 Multiplicity = 2
Symbolic Z-Matrix:

C	−7.7444	0.50102	1.11674
C	−6.54698	−0.20809	1.05124
C	−6.04703	−0.63169	−0.18615
C	−6.75079	−0.35427	−1.35067
C	−7.95394	0.35573	−1.29519
C	−8.45885	0.78832	−0.06618

H	−5.98834	−0.42887	1.95086
H	−6.38127	−0.66	−2.32219
O	−8.58347	0.66552	−2.47215
O	−9.6565	1.45215	−0.01306
O	−8.31071	0.96594	2.26399
C	−9.55309	2.87257	0.1592
H	−10.5744	3.2522	0.1616
H	−9.07213	3.11498	1.10994
H	−8.9973	3.32178	−0.66962
C	−7.6626	0.68805	3.49878
H	−6.66505	1.13877	3.54003
H	−8.29126	1.13176	4.26866
H	−7.58339	−0.39028	3.67348
C	−9.86521	0.05567	−2.68225
H	−10.5767	0.3567	−1.9119
H	−10.20453	0.40251	−3.65779
H	−9.77193	−1.03593	−2.69871
C	−4.74443	−1.37956	−0.24263
H	−4.70832	−2.1596	0.52627
H	−4.61071	−1.84742	−1.22277
O	−3.67343	−0.44209	−0.00732
C	−2.39892	−0.89205	0.02221
C	−1.41661	0.08134	0.26469
C	−2.01458	−2.22783	−0.16312
C	−0.08099	−0.27054	0.31301
H	−1.73461	1.10497	0.41711
C	−0.67081	−2.56957	−0.10328
H	−2.74843	−2.99878	−0.35199
C	0.31781	−1.60615	0.12327
H	0.64208	0.50739	0.52994
H	−0.36282	−3.59937	−0.23453
C	1.72248	−2.08134	0.17843
O	2.05115	−3.23971	0.28627
N	2.69647	−1.05479	0.0675
H	2.44956	−0.08586	−0.16674
N	3.97303	−1.324	0.16775
C	4.81852	−0.25074	−0.02432
N	6.10214	−0.71655	0.12098
H	6.10337	−1.70865	0.33161
S	4.31922	1.31867	−0.36867
C	7.36069	−0.12806	0.04103
C	8.45809	−0.98014	0.28704
C	7.59654	1.22328	−0.26447
C	9.75273	−0.50315	0.23224
H	8.28205	−2.02417	0.52308
C	8.89888	1.69928	−0.31896
H	6.76798	1.88518	−0.45625
C	9.96284	0.84036	−0.07167
H	10.60075	−1.14646	0.41936
H	9.09961	2.73547	−0.55293
N	11.33983	1.35586	−0.13109
O	12.25056	0.56658	0.0906

O 11.49264 2.54119 −0.39804

Stoichiometry C24H23N4O7S(2)
Framework group C1[X(C24H23N4O7S)]
Deg. of freedom 171
Full point group C1 NOp 1
Largest Abelian subgroup C1 NOp 1
Largest concise Abelian subgroup C1 NOp 1

Standard orientation:

Center Number	Atomic Number	Atomic Type	Coordinates (Angstroms) X	Y	Z
1	6	0	7.744404	−0.501022	1.116737
2	6	0	6.546978	0.208093	1.051237
3	6	0	6.047033	0.631694	−0.186153
4	6	0	6.750791	0.354267	−1.350667
5	6	0	7.953939	−0.355732	−1.295193
6	6	0	8.458849	−0.788324	−0.066177
7	1	0	5.988338	0.428867	1.950857
8	1	0	6.381268	0.659998	−2.322185
9	8	0	8.583472	−0.665521	−2.472147
10	8	0	9.656503	−1.452146	−0.013063
11	8	0	8.310706	−0.965942	2.263995
12	6	0	9.553090	−2.872565	0.159203
13	1	0	10.574403	−3.252200	0.161602
14	1	0	9.072135	−3.114978	1.109939
15	1	0	8.997296	−3.321780	−0.669615
16	6	0	7.662600	−0.688047	3.498775
17	1	0	6.665053	−1.138767	3.540025
18	1	0	8.291264	−1.131757	4.268657
19	1	0	7.583393	0.390283	3.673482
20	6	0	9.865209	−0.055670	−2.682249
21	1	0	10.576702	−0.356701	−1.911900
22	1	0	10.204527	−0.402507	−3.657787
23	1	0	9.771927	1.035930	−2.698707
24	6	0	4.744430	1.379564	−0.242634
25	1	0	4.708321	2.159601	0.526267
26	1	0	4.610708	1.847424	−1.222766
27	8	0	3.673430	0.442092	−0.007324
28	6	0	2.398920	0.892046	0.022211
29	6	0	1.416610	−0.081339	0.264685
30	6	0	2.014576	2.227831	−0.163118
31	6	0	0.080994	0.270535	0.313012
32	1	0	1.734614	−1.104971	0.417105
33	6	0	0.670812	2.569566	−0.103277
34	1	0	2.748433	2.998777	−0.351993
35	6	0	−0.317814	1.606145	0.123265
36	1	0	−0.642079	−0.507391	0.529936
37	1	0	0.362818	3.599365	−0.234530
38	6	0	−1.722480	2.081337	0.178431
39	8	0	−2.051154	3.239712	0.286272
40	7	0	−2.696467	1.054788	0.067499

41	1	0	−2.449556	0.085864	−0.166735
42	7	0	−3.973028	1.324003	0.167750
43	6	0	−4.818518	0.250742	−0.024319
44	7	0	−6.102136	0.716549	0.120982
45	1	0	−6.103373	1.708648	0.331609
46	16	0	−4.319223	−1.318672	−0.368668
47	6	0	−7.360690	0.128055	0.041025
48	6	0	−8.458091	0.980142	0.287038
49	6	0	−7.596541	−1.223277	−0.264468
50	6	0	−9.752734	0.503145	0.232242
51	1	0	−8.282045	2.024170	0.523076
52	6	0	−8.898879	−1.699275	−0.318960
53	1	0	−6.767975	−1.885179	−0.456248
54	6	0	−9.962839	−0.840363	−0.071667
55	1	0	−10.600755	1.146458	0.419363
56	1	0	−9.099613	−2.735465	−0.552928
57	7	0	−11.339827	−1.355863	−0.131088
58	8	0	−12.250559	−0.566578	0.090596
59	8	0	−11.492638	−2.541187	−0.398042

Rotational constants (GHZ): 0.3176963 0.0189554 0.0186597
Standard basis: 6-311++G(d,p) (5D, 7F)
There are 961 symmetry adapted basis functions of A symmetry.
Integral buffers will be 131072 words long.
Raffenetti 2 integral format.
Two-electron integral symmetry is turned on.
961 basis functions, 1517 primitive gaussians, 997 cartesian basis functions
134 alpha electrons 133 beta electrons
nuclear repulsion energy 3446.3251842152 Hartrees.
NAtoms= 59 NActive= 59 NUniq= 59 SFac= 1.00D+00 NAtFMM= 60 NAOKFM=F Big=F

Polarizable Continuum Model (PCM)
===================================

Model : PCM.
Atomic radii : UFF (Universal Force Field).
Polarization charges : Total charges.
Charge compensation : None.
Solution method : Matrix inversion.
Cavity type : Scaled VdW (van der Waals Surface) (Alpha=1.100).
Cavity algorithm : GePol (No added spheres)
Default sphere list used, NSphG= 59.
Lebedev-Laikov grids with approx. 5.0 points / Ang**2.
Smoothing algorithm: Karplus/York (Gamma=1.0000).
Polarization charges: spherical gaussians, with
point-specific exponents (IZeta= 3).
Self-potential: point-specific (ISelfS= 7).
Self-field : sphere-specific E.n sum rule (ISelfD= 2).
Solvent : DiMethylSulfoxide, Eps= 46.826000 Eps(inf)= 2.007889

3f-r3.out

Title Card Required

Charge = 0 Multiplicity = 2
Symbolic Z-Matrix:

C	−7.25233	−0.34735	−1.06211
C	−5.89638	−0.07126	−0.88072
C	−5.48945	0.80058	0.1389
C	−6.43724	1.40351	0.96039
C	−7.80007	1.13735	0.77862
C	−8.21822	0.25814	−0.22704
H	−5.14577	−0.53983	−1.5041
H	−6.14893	2.07433	1.76332
O	−8.69317	1.70167	1.65476
O	−9.5573	0.01663	−0.41765
O	−7.75179	−1.19358	−2.00813
C	−10.01601	−1.24121	0.09155
H	−11.08868	−1.2781	−0.10845
H	−9.52205	−2.07458	−0.41778
H	−9.84401	−1.31113	1.17201
C	−6.83439	−1.83877	−2.87969
H	−6.13421	−2.47895	−2.32866
H	−7.43872	−2.45508	−3.54645
H	−6.26574	−1.11347	−3.47466
C	−9.63355	2.62127	1.08718
H	−10.26926	2.13338	0.34444
H	−10.24263	2.98007	1.91921
H	−9.11483	3.47236	0.62758
C	−4.0288	1.13436	0.30606
H	−3.6881	1.8082	−0.49339
H	−3.85886	1.6365	1.26638
O	−3.26556	−0.07943	0.24459
C	−1.91412	−0.01327	0.32346
C	−1.24095	−1.24458	0.22409
C	−1.18134	1.17377	0.49716
C	0.13969	−1.29182	0.29318
H	−1.83103	−2.14541	0.09505
C	0.20527	1.11582	0.56507
H	−1.68119	2.13055	0.58223
C	0.88502	−0.107	0.46407
H	0.65912	−2.23983	0.21753
H	0.78643	2.02182	0.69996
C	2.35266	−0.10933	0.54584
O	3.04424	0.90551	0.69885
N	2.93533	−1.3931	0.4261
N	4.2443	−1.28851	0.54213
C	5.1015	−2.36967	0.5128
N	6.43999	−2.02493	0.51086
S	4.62061	−3.95887	0.56012
C	7.10355	−0.83891	0.15585
C	6.57442	0.10538	−0.74392
C	8.38751	−0.63235	0.69684
C	7.30336	1.24375	−1.06653

H	5.61329	−0.05624	−1.21602
C	9.12189	0.49471	0.36358
H	8.79722	−1.35931	1.39155
C	8.56667	1.42942	−0.5106
H	6.91294	1.98023	−1.75726
H	10.10808	0.66994	0.77427
N	9.33518	2.62763	−0.85953
O	8.81646	3.43499	−1.63097
O	10.45415	2.75319	−0.3602
H	4.58588	−0.33069	0.70998
H	7.02917	−2.81759	0.73

Stoichiometry C24H23N4O7S(2)
Framework group C1[X(C24H23N4O7S)]
Deg. of freedom 171
Full point group C1 NOp 1
Largest Abelian subgroup C1 NOp 1
Largest concise Abelian subgroup C1 NOp 1

Standard orientation:

Center Number	Atomic Number	Atomic Type	Coordinates (Angstroms) X	Y	Z
1	6	0	−7.252332	0.347345	1.062105
2	6	0	−5.896380	0.071256	0.880719
3	6	0	−5.489449	−0.800583	−0.138900
4	6	0	−6.437236	−1.403505	−0.960385
5	6	0	−7.800065	−1.137350	−0.778616
6	6	0	−8.218218	−0.258137	0.227040
7	1	0	−5.145773	0.539825	1.504097
8	1	0	−6.148926	−2.074330	−1.763323
9	8	0	−8.693173	−1.701673	−1.654759
10	8	0	−9.557303	−0.016628	0.417646
11	8	0	−7.751787	1.193582	2.008130
12	6	0	−10.016015	1.241210	−0.091549
13	1	0	−11.088678	1.278101	0.108453
14	1	0	−9.522055	2.074582	0.417775
15	1	0	−9.844007	1.311130	−1.172006
16	6	0	−6.834394	1.838765	2.879690
17	1	0	−6.134206	2.478948	2.328662
18	1	0	−7.438720	2.455080	3.546446
19	1	0	−6.265743	1.113468	3.474663
20	6	0	−9.633549	−2.621274	−1.087181
21	1	0	−10.269262	−2.133380	−0.344441
22	1	0	−10.242634	−2.980068	−1.919214
23	1	0	−9.114826	−3.472361	−0.627581
24	6	0	−4.028796	−1.134356	−0.306062
25	1	0	−3.688103	−1.808202	0.493390
26	1	0	−3.858856	−1.636503	−1.266383
27	8	0	−3.265557	0.079427	−0.244593
28	6	0	−1.914119	0.013265	−0.323456
29	6	0	−1.240951	1.244582	−0.224088
30	6	0	−1.181341	−1.173773	−0.497159

31	6	0	0.139692	1.291816	−0.293184
32	1	0	−1.831031	2.145409	−0.095048
33	6	0	0.205272	−1.115818	−0.565066
34	1	0	−1.681193	−2.130548	−0.582231
35	6	0	0.885020	0.107004	−0.464074
36	1	0	0.659120	2.239832	−0.217529
37	1	0	0.786432	−2.021816	−0.699959
38	6	0	2.352664	0.109325	−0.545844
39	8	0	3.044236	−0.905511	−0.698847
40	7	0	2.935332	1.393101	−0.426104
41	7	0	4.244298	1.288513	−0.542130
42	6	0	5.101503	2.369671	−0.512799
43	7	0	6.439994	2.024925	−0.510864
44	16	0	4.620612	3.958874	−0.560122
45	6	0	7.103554	0.838913	−0.155852
46	6	0	6.574420	−0.105379	0.743917
47	6	0	8.387506	0.632349	−0.696842
48	6	0	7.303360	−1.243746	1.066531
49	1	0	5.613291	0.056244	1.216021
50	6	0	9.121888	−0.494709	−0.363579
51	1	0	8.797215	1.359310	−1.391549
52	6	0	8.566671	−1.429418	0.510595
53	1	0	6.912940	−1.980232	1.757263
54	1	0	10.108077	−0.669942	−0.774271
55	7	0	9.335182	−2.627629	0.859530
56	8	0	8.816457	−3.434994	1.630965
57	8	0	10.454153	−2.753192	0.360195
58	1	0	4.585875	0.330688	−0.709984
59	1	0	7.029174	2.817594	−0.729998

Rotational constants (GHZ): 0.2469945 0.0220726 0.0210274

Standard basis: 6-311++G(d,p) (5D, 7F)

There are 961 symmetry adapted basis functions of A symmetry.

Integral buffers will be 131072 words long.

Raffenetti 2 integral format.

Two-electron integral symmetry is turned on.

961 basis functions, 1517 primitive gaussians, 997 cartesian basis functions

134 alpha electrons 133 beta electrons

nuclear repulsion energy 3504.7123107818 Hartrees.

NAtoms= 59 NActive= 59 NUniq= 59 SFac= 1.00D+00 NAtFMM= 60 NAOKFM=F Big=F

Polarizable Continuum Model (PCM)

=================================

Model : PCM.

Atomic radii : UFF (Universal Force Field).

Polarization charges : Total charges.

Charge compensation : None.

Solution method : Matrix inversion.

Cavity type : Scaled VdW (van der Waals Surface) (Alpha=1.100).

Cavity algorithm : GePol (No added spheres)

Default sphere list used, NSphG= 59.

Lebedev-Laikov grids with approx. 5.0 points / Ang**2.

Smoothing algorithm: Karplus/York (Gamma=1.0000).
Polarization charges: spherical gaussians, with
point-specific exponents (IZeta= 3).
Self-potential: point-specific (ISelfS= 7).
Self-field : sphere-specific E.n sum rule (ISelfD= 2).
Solvent : DiMethylSulfoxide, Eps= 46.826000 Eps(inf)= 2.007889

6a.out

Title Card Required

Charge = 0 Multiplicity = 1
Symbolic Z-Matrix:

C	−8.56406	0.95725	0.04481
C	−7.18897	0.69089	0.08968
C	−6.73393	−0.6323	0.16753
C	−7.65371	−1.68901	0.20055
C	−9.02868	−1.42243	0.15642
C	−9.48376	−0.09942	0.07857
H	−6.48653	1.49764	0.06462
H	−7.30612	−2.69932	0.25952
O	−9.96769	−2.50025	0.19097
O	−10.887	0.17231	0.03385
O	−9.02901	2.30701	−0.03543
C	−11.11688	1.37378	−0.7069
H	−12.16682	1.5774	−0.73904
H	−10.61009	2.18799	−0.23234
H	−10.7459	1.25508	−1.7035
C	−8.09412	3.17261	0.61356
H	−7.14187	3.0943	0.13191
H	−8.4422	4.18258	0.55362
H	−7.99863	2.88854	1.64073
C	−10.31331	−2.78756	1.5485
H	−10.74966	−1.91941	1.99679
H	−11.01614	−3.59393	1.57458
H	−9.4328	−3.06415	2.08993
C	−5.22309	−0.92546	0.21671
H	−5.09077	−1.80405	0.81293
O	−4.49322	−1.12451	−0.99684
C	−3.14092	−0.69741	−0.81427
C	−2.10845	−1.3133	−1.53426
C	−2.84818	0.33685	0.08477
C	−0.78287	−0.89595	−1.35407
H	−2.33225	−2.10233	−2.22148
C	−1.52262	0.75403	0.2652
H	−3.63672	0.80801	0.6335
C	−0.4899	0.13698	−0.45344
H	0.00556	−1.36608	−1.90405
H	−1.299	1.54359	0.95188
C	0.96705	0.59348	−0.25334
C	3.13428	0.55373	−0.12454

O	2.08496	0.01874	−0.93843
N	1.32381	1.53555	0.55719
N	2.71879	1.51006	0.64004
S	4.81333	−0.03629	−0.15631
C	5.9216	1.29621	0.24883
H	5.69339	1.66558	1.22678
C	7.37294	0.78204	0.2197
C	8.11197	0.8377	−0.96975
C	7.95466	0.25818	1.38216
C	9.43236	0.36894	−0.99692
H	7.66803	1.23804	−1.85719
C	9.27522	−0.21006	1.35499
H	7.39067	0.21569	2.29049
C	10.01402	−0.15465	0.16556
H	9.99633	0.41078	−1.90526
H	9.71947	−0.60995	2.24241
Br	11.8138	−0.79286	0.12913
H	5.80792	2.08468	−0.46554
H	−4.75983	−0.11164	0.73438

Stoichiometry C25H23BrN2O5S
Framework group C1[X(C25H23BrN2O5S)]
Deg. of freedom 165
Full point group C1 NOp 1
Largest Abelian subgroup C1 NOp 1
Largest concise Abelian subgroup C1 NOp 1

Standard orientation:

Center Number	Atomic Number	Atomic Type	Coordinates (Angstroms) X	Y	Z
1	6	0	−8.545308	0.963203	0.043570
2	6	0	−7.170309	0.696358	0.088269
3	6	0	−6.715691	−0.627074	0.164447
4	6	0	−7.635803	−1.683541	0.195960
5	6	0	−9.010681	−1.416481	0.152001
6	6	0	−9.465344	−0.093227	0.075826
7	1	0	−6.467620	1.502923	0.064358
8	1	0	−7.288532	−2.694036	0.253651
9	8	0	−9.950030	−2.494053	0.185014
10	8	0	−10.868489	0.178996	0.031283
11	8	0	−9.009829	2.313214	−0.034956
12	6	0	−11.097906	1.381504	−0.707918
13	1	0	−12.147775	1.585494	−0.739929
14	1	0	−10.590921	2.194942	−0.232222
15	1	0	−10.726830	1.264005	−1.704625
16	6	0	−8.074757	3.177668	0.615294
17	1	0	−7.122466	3.099691	0.133661
18	1	0	−8.422517	4.187821	0.556630
19	1	0	−7.979485	2.892224	1.642094
20	6	0	−10.295914	−2.783033	1.542124
21	1	0	−10.732054	−1.915334	1.991496
22	1	0	−10.998995	−3.589220	1.567056

23	1	0	−9.415561	−3.060605	2.083301
24	6	0	−5.204942	−0.920767	0.213429
25	1	0	−5.072976	−1.800175	0.808515
26	8	0	−4.474980	−1.118447	−1.000280
27	6	0	−3.122568	−0.692005	−0.816980
28	6	0	−2.090201	−1.307267	−1.537647
29	6	0	−2.829624	0.340982	0.083453
30	6	0	−0.764511	−0.890573	−1.356731
31	1	0	−2.314153	−2.095326	−2.225922
32	6	0	−1.503965	0.757516	0.264601
33	1	0	−3.618097	0.811660	0.632701
34	6	0	−0.471342	0.141088	−0.454708
35	1	0	0.023838	−1.360220	−1.907227
36	1	0	−1.280184	1.546099	0.952348
37	6	0	0.985729	0.596868	−0.253826
38	6	0	3.152926	0.556281	−0.124800
39	8	0	2.103544	0.022683	−0.939530
40	7	0	1.342673	1.537769	0.557987
41	7	0	2.737633	1.511739	0.640982
42	16	0	4.831802	−0.034217	−0.157127
43	6	0	5.940428	1.297411	0.249902
44	1	0	5.712203	1.665563	1.228304
45	6	0	7.391609	0.782825	0.220286
46	6	0	8.130817	0.839824	−0.968997
47	6	0	7.973024	0.257266	1.382128
48	6	0	9.451063	0.370686	−0.996607
49	1	0	7.687107	1.241461	−1.855970
50	6	0	9.293434	−0.211353	1.354520
51	1	0	7.408894	0.213759	2.290334
52	6	0	10.032409	−0.154604	0.165260
53	1	0	10.015161	0.413547	−1.904820
54	1	0	9.737447	−0.612540	2.241471
55	35	0	11.831994	−0.793325	0.128225
56	1	0	5.827079	2.086851	−0.463448
57	1	0	−4.741496	−0.107773	0.732232

Rotational constants (GHZ): 0.4573226 0.0149723 0.0147207
Standard basis: 6-311++G(d,p) (5D, 7F)
There are 943 symmetry adapted basis functions of A symmetry.
Integral buffers will be 131072 words long.
Raffenetti 2 integral format.
Two-electron integral symmetry is turned on.
943 basis functions, 1503 primitive gaussians, 979 cartesian basis functions
139 alpha electrons 139 beta electrons
nuclear repulsion energy 3499.1181818346 Hartrees.
NAtoms= 57 NActive= 57 NUniq= 57 SFac= 1.00D+00 NAtFMM= 60 NAOKFM=F Big=F

Polarizable Continuum Model (PCM)
=================================
Model : PCM.
Atomic radii : UFF (Universal Force Field).
Polarization charges : Total charges.

Charge compensation : None.
Solution method : Matrix inversion.
Cavity type : Scaled VdW (van der Waals Surface) (Alpha=1.100).
Cavity algorithm : GePol (No added spheres)
Default sphere list used, NSphG= 57.
Lebedev-Laikov grids with approx. 5.0 points / Ang**2.
Smoothing algorithm: Karplus/York (Gamma=1.0000).
Polarization charges: spherical gaussians, with
point-specific exponents (IZeta= 3).
Self-potential: point-specific (ISelfS= 7).
Self-field : sphere-specific E.n sum rule (ISelfD= 2).
Solvent : DiMethylSulfoxide, Eps= 46.826000 Eps(inf)= 2.007889

R16a.out

Title Card Required

Charge = 0 Multiplicity = 2
Symbolic Z-Matrix:

C	8.75717	−0.63304	−0.45078
C	7.37681	−0.70637	−0.21526
C	6.65089	0.45961	0.06503
C	7.30552	1.69771	0.11133
C	8.68415	1.77096	−0.12462
C	9.41051	0.60668	−0.40684
H	6.87713	−1.65206	−0.24939
H	6.75216	2.58774	0.32701
O	9.35091	3.03431	−0.07735
O	10.81843	0.68603	−0.64926
O	9.49797	−1.82244	−0.73527
C	11.20852	−0.3498	−1.55447
H	12.26134	−0.28725	−1.73564
H	10.97599	−1.3027	−1.12738
H	10.68031	−0.23408	−2.47781
C	8.88185	−2.93335	−0.07875
H	7.87837	−3.04426	−0.43271
H	9.43513	−3.82459	−0.28969
H	8.87079	−2.76224	0.97752
C	9.81053	3.27899	1.25409
H	10.48855	2.50395	1.5451
H	10.3116	4.22364	1.2903
H	8.9755	3.29217	1.92322
C	5.13389	0.38389	0.32313
H	4.95743	0.23907	1.36862
H	4.6732	1.29624	0.00601
O	4.57527	−0.71052	−0.40953
C	3.16674	−0.77137	−0.16625
C	2.28145	−0.06998	−0.99643
C	2.67141	−1.53109	0.90248
C	0.90168	−0.12324	−0.75417
H	2.65901	0.50643	−1.81491

C	1.29178	−1.58379	1.14479
H	3.34622	−2.0703	1.53384
C	0.40708	−0.87804	0.31806
H	0.22638	0.41287	−1.38752
H	0.91363	−2.1627	1.9615
C	−1.10872	−0.93176	0.58927
C	−3.2002	−0.3538	0.69885
O	−2.08825	−0.24323	−0.19715
N	−1.64449	−1.59865	1.55894
N	−2.98987	−1.22576	1.62991
S	−4.69407	0.60509	0.55323
C	−6.06484	−0.31431	1.22181
H	−5.90163	−1.01749	2.01174
C	−7.48565	−0.09744	0.66574
C	−7.95003	−0.88102	−0.39998
C	−8.31487	0.88429	1.22646
C	−9.2441	−0.68484	−0.9022
H	−7.31688	−1.62886	−0.82983
C	−9.60842	1.08066	0.72346
H	−7.96029	1.48402	2.03852
C	−10.07326	0.29601	−0.34038
H	−9.59894	−1.28402	−1.71451
H	−10.24158	1.8297	1.15175
Br	−11.8369	0.56393	−1.02362

Stoichiometry C25H22BrN2O5S(2)
Framework group C1[X(C25H22BrN2O5S)]
Deg. of freedom 162
Full point group C1 NOp 1
Largest Abelian subgroup C1 NOp 1
Largest concise Abelian subgroup C1 NOp 1

Standard orientation:

Center Number	Atomic Number	Atomic Type	Coordinates (Angstroms) X	Y	Z
1	6	0	8.757165	−0.633039	−0.450782
2	6	0	7.376806	−0.706365	−0.215260
3	6	0	6.650889	0.459608	0.065031
4	6	0	7.305519	1.697707	0.111332
5	6	0	8.684150	1.770964	−0.124621
6	6	0	9.410511	0.606675	−0.406842
7	1	0	6.877128	−1.652061	−0.249393
8	1	0	6.752161	2.587739	0.327007
9	8	0	9.350914	3.034311	−0.077351
10	8	0	10.818431	0.686028	−0.649263
11	8	0	9.497969	−1.822439	−0.735269
12	6	0	11.208515	−0.349797	−1.554474
13	1	0	12.261338	−0.287253	−1.735643
14	1	0	10.975993	−1.302703	−1.127382
15	1	0	10.680314	−0.234081	−2.477812
16	6	0	8.881852	−2.933345	−0.078750
17	1	0	7.878371	−3.044258	−0.432712

18	1	0	9.435133	−3.824585	−0.289690
19	1	0	8.870794	−2.762241	0.977517
20	6	0	9.810529	3.278990	1.254089
21	1	0	10.488547	2.503953	1.545095
22	1	0	10.311603	4.223636	1.290295
23	1	0	8.975502	3.292174	1.923222
24	6	0	5.133893	0.383891	0.323127
25	1	0	4.957434	0.239070	1.368621
26	1	0	4.673200	1.296241	0.006010
27	8	0	4.575268	−0.710519	−0.409534
28	6	0	3.166738	−0.771374	−0.166245
29	6	0	2.281449	−0.069979	−0.996432
30	6	0	2.671413	−1.531091	0.902475
31	6	0	0.901684	−0.123236	−0.754166
32	1	0	2.659013	0.506427	−1.814911
33	6	0	1.291781	−1.583790	1.144790
34	1	0	3.346223	−2.070296	1.533842
35	6	0	0.407075	−0.878040	0.318055
36	1	0	0.226379	0.412871	−1.387517
37	1	0	0.913628	−2.162704	1.961501
38	6	0	−1.108716	−0.931759	0.589268
39	6	0	−3.200204	−0.353803	0.698849
40	8	0	−2.088251	−0.243225	−0.197153
41	7	0	−1.644487	−1.598653	1.558941
42	7	0	−2.989873	−1.225755	1.629909
43	16	0	−4.694069	0.605093	0.553225
44	6	0	−6.064843	−0.314307	1.221807
45	1	0	−5.901631	−1.017492	2.011738
46	6	0	−7.485654	−0.097442	0.665739
47	6	0	−7.950032	−0.881022	−0.399984
48	6	0	−8.314871	0.884291	1.226461
49	6	0	−9.244103	−0.684835	−0.902203
50	1	0	−7.316877	−1.628858	−0.829828
51	6	0	−9.608418	1.080656	0.723460
52	1	0	−7.960291	1.484017	2.038517
53	6	0	−10.073264	0.296013	−0.340383
54	1	0	−9.598935	−1.284022	−1.714514
55	1	0	−10.241582	1.829698	1.151750
56	35	0	−11.836904	0.563926	−1.023621

Rotational constants (GHZ): 0.4001542 0.0148916 0.0146896
Standard basis: 6-311++G(d,p) (5D, 7F)
There are 936 symmetry adapted basis functions of A symmetry.
Integral buffers will be 131072 words long.
Raffenetti 2 integral format.
Two-electron integral symmetry is turned on.
936 basis functions, 1494 primitive gaussians, 972 cartesian basis functions
139 alpha electrons 138 beta electrons
nuclear repulsion energy 3460.6994906169 Hartrees.
NAtoms= 56 NActive= 56 NUniq= 56 SFac= 1.00D+00 NAtFMM= 60 NAOKFM=F Big=F

Polarizable Continuum Model (PCM)

==================================

Model : PCM.
Atomic radii : UFF (Universal Force Field).
Polarization charges : Total charges.
Charge compensation : None.
Solution method : Matrix inversion.
Cavity type : Scaled VdW (van der Waals Surface) (Alpha=1.100).
Cavity algorithm : GePol (No added spheres)
Default sphere list used, NSphG= 56.
Lebedev-Laikov grids with approx. 5.0 points / Ang**2.
Smoothing algorithm: Karplus/York (Gamma=1.0000).
Polarization charges: spherical gaussians, with
point-specific exponents (IZeta= 3).
Self-potential: point-specific (ISelfS= 7).
Self-field : sphere-specific E.n sum rule (ISelfD= 2).
Solvent : DiMethylSulfoxide, Eps= 46.826000 Eps(inf)= 2.007889

R26a.out

Title Card Required

Charge = 0 Multiplicity = 2
Symbolic Z-Matrix:

C	−8.56406	0.95725	0.04481
C	−7.18897	0.69089	0.08968
C	−6.73393	−0.6323	0.16753
C	−7.65371	−1.68901	0.20055
C	−9.02868	−1.42243	0.15642
C	−9.48376	−0.09942	0.07857
H	−6.48653	1.49764	0.06462
H	−7.30612	−2.69932	0.25952
O	−9.96769	−2.50025	0.19097
O	−10.887	0.17231	0.03385
O	−9.02901	2.30701	−0.03543
C	−11.11688	1.37378	−0.7069
H	−12.16682	1.5774	−0.73904
H	−10.61009	2.18799	−0.23234
H	−10.7459	1.25508	−1.7035
C	−8.09412	3.17261	0.61356
H	−7.14187	3.0943	0.13191
H	−8.4422	4.18258	0.55362
H	−7.99863	2.88854	1.64073
C	−10.31331	−2.78756	1.5485
H	−10.74966	−1.91941	1.99679
H	−11.01614	−3.59393	1.57458
H	−9.4328	−3.06415	2.08993
C	−5.22309	−0.92546	0.21671
H	−4.71937	−0.9803	1.15907
O	−4.49322	−1.12451	−0.99684
C	−3.14092	−0.69741	−0.81427
C	−2.10845	−1.3133	−1.53426

C	−2.84818	0.33685	0.08477
C	−0.78287	−0.89595	−1.35407
H	−2.33225	−2.10233	−2.22148
C	−1.52262	0.75403	0.2652
H	−3.63672	0.80801	0.6335
C	−0.4899	0.13698	−0.45344
H	0.00556	−1.36608	−1.90405
H	−1.299	1.54359	0.95188
C	0.96705	0.59348	−0.25334
C	3.13428	0.55373	−0.12454
O	2.08496	0.01874	−0.93843
N	1.32381	1.53555	0.55719
N	2.71879	1.51006	0.64004
S	4.81333	−0.03629	−0.15631
C	5.9216	1.29621	0.24883
H	5.69339	1.66558	1.22678
C	7.37294	0.78204	0.2197
C	8.11197	0.8377	−0.96975
C	7.95466	0.25818	1.38216
C	9.43236	0.36894	−0.99692
H	7.66803	1.23804	−1.85719
C	9.27522	−0.21006	1.35499
H	7.39067	0.21569	2.29049
C	10.01402	−0.15465	0.16556
H	9.99633	0.41078	−1.90526
H	9.71947	−0.60995	2.24241
Br	11.8138	−0.79286	0.12913
H	5.80792	2.08468	−0.46554

Stoichiometry C25H22BrN2O5S(2)
Framework group C1[X(C25H22BrN2O5S)]
Deg. of freedom 162
Full point group C1 NOp 1
Largest Abelian subgroup C1 NOp 1
Largest concise Abelian subgroup C1 NOp 1

Standard orientation:

Center Number	Atomic Number	Atomic Type	Coordinates (Angstroms) X	Y	Z
1	6	0	−8.564058	0.957250	0.044806
2	6	0	−7.188970	0.690890	0.089678
3	6	0	−6.733933	−0.632301	0.167533
4	6	0	−7.653714	−1.689010	0.200551
5	6	0	−9.028680	−1.422433	0.156420
6	6	0	−9.483762	−0.099421	0.078567
7	1	0	−6.486534	1.497640	0.064618
8	1	0	−7.306122	−2.699321	0.259523
9	8	0	−9.967691	−2.500252	0.190968
10	8	0	−10.886997	0.172309	0.033848
11	8	0	−9.029007	2.307013	−0.035431
12	6	0	−11.116882	1.373775	−0.706900
13	1	0	−12.166818	1.577398	−0.739043

14	1	0	−10.610088	2.187993	−0.232337
15	1	0	−10.745898	1.255083	−1.703500
16	6	0	−8.094119	3.172609	0.613564
17	1	0	−7.141866	3.094295	0.131910
18	1	0	−8.442199	4.182576	0.553619
19	1	0	−7.998626	2.888542	1.640725
20	6	0	−10.313311	−2.787558	1.548500
21	1	0	−10.749662	−1.919405	1.996790
22	1	0	−11.016139	−3.593929	1.574581
23	1	0	−9.432802	−3.064147	2.089927
24	6	0	−5.223087	−0.925462	0.216706
25	1	0	−4.719371	−0.980295	1.159067
26	8	0	−4.493220	−1.124508	−0.996837
27	6	0	−3.140916	−0.697407	−0.814272
28	6	0	−2.108452	−1.313295	−1.534264
29	6	0	−2.848176	0.336851	0.084768
30	6	0	−0.782867	−0.895954	−1.354066
31	1	0	−2.332248	−2.102326	−2.221475
32	6	0	−1.522623	0.754032	0.265198
33	1	0	−3.636724	0.808005	0.633499
34	6	0	−0.489902	0.136981	−0.453435
35	1	0	0.005556	−1.366079	−1.904047
36	1	0	−1.298998	1.543586	0.951880
37	6	0	0.967054	0.593475	−0.253340
38	6	0	3.134280	0.553728	−0.124540
39	8	0	2.084958	0.018737	−0.938434
40	7	0	1.323811	1.535551	0.557192
41	7	0	2.718790	1.510061	0.640041
42	16	0	4.813334	−0.036293	−0.156309
43	6	0	5.921601	1.296211	0.248829
44	1	0	5.693387	1.665576	1.226776
45	6	0	7.372937	0.782036	0.219701
46	6	0	8.111974	0.837703	−0.969752
47	6	0	7.954664	0.258182	1.382156
48	6	0	9.432362	0.368938	−0.996917
49	1	0	7.668026	1.238039	−1.857193
50	6	0	9.275216	−0.210064	1.354992
51	1	0	7.390665	0.215692	2.290491
52	6	0	10.014020	−0.154647	0.165564
53	1	0	9.996330	0.410782	−1.905258
54	1	0	9.719467	−0.609950	2.242412
55	35	0	11.813798	−0.792859	0.129134
56	1	0	5.807915	2.084680	−0.465542

Rotational constants (GHZ): 0.4582201 0.0149855 0.0147345

Standard basis: 6-311++G(d,p) (5D, 7F)

There are 936 symmetry adapted basis functions of A symmetry.

Integral buffers will be 131072 words long.

Raffenetti 2 integral format.

Two-electron integral symmetry is turned on.

936 basis functions, 1494 primitive gaussians, 972 cartesian basis functions

139 alpha electrons 138 beta electrons

```
        nuclear repulsion energy        3469.7887691376 Hartrees.
 NAtoms=    56 NActive=    56 NUniq=    56 SFac= 1.00D+00 NAtFMM=    60 NAOKFM=F Big=F
 ------------------------------------------------------------------------------
 Polarizable Continuum Model (PCM)
 =================================
 Model                : PCM.
 Atomic radii         : UFF (Universal Force Field).
 Polarization charges : Total charges.
 Charge compensation  : None.
 Solution method      : Matrix inversion.
 Cavity type          : Scaled VdW (van der Waals Surface) (Alpha=1.100).
 Cavity algorithm     : GePol (No added spheres)
                        Default sphere list used, NSphG = 56.
                        Lebedev-Laikov grids with approx.  5.0 points / Ang**2.
                        Smoothing algorithm: Karplus/York (Gamma=1.0000).
                        Polarization charges: spherical gaussians, with
                                              point-specific exponents (IZeta = 3).
                        Self-potential: point-specific (ISelfS = 7).
                        Self-field     : sphere-specific E.n sum rule (ISelfD = 2).
 Solvent              : DiMethylSulfoxide, Eps = 46.826000 Eps(inf) = 2.007889
 ------------------------------------------------------------------------------
```

6b.out

```
-------------------
 Title Card Required
 -------------------
 Charge =  0 Multiplicity = 1
 Symbolic Z-Matrix:
 C                    8.39915    1.39148   −0.47886
 C                    7.02092    1.13959   −0.4486
 C                    6.53039    0.00443    0.21072
 C                    7.41809   −0.87883    0.83981
 C                    8.79632   −0.62696    0.80955
 C                    9.28687    0.50819    0.15019
 H                    6.34313    1.81398   −0.92891
 H                    7.04354   −1.74553    1.34324
 O                    8.89966    2.54983   −1.15163
 O                   10.69325    0.76516    0.11925
 O                    9.70212   −1.52827    1.45149
 C                    9.05286   −2.14163    2.56825
 H                    8.1934    −2.68118    2.22896
 H                    9.73062   −2.81601    3.04861
 H                    8.74874   −1.38665    3.26281
 C                   11.40521   −0.47442    0.15744
 H                   11.15833   −0.99999    1.05618
 H                   11.13248   −1.06862   −0.68958
 H                   12.45754   −0.28218    0.1343
 C                    8.96165    3.64461   −0.23374
 H                    9.61313    3.39374    0.57715
 H                    9.33619    4.51134   −0.73714
 H                    7.98202    3.84793    0.14558
 C                    5.01586   −0.27241    0.24396
```

H	4.84714	−1.3281	0.28807
H	4.58692	0.19369	1.10632
O	4.40822	0.25849	−0.93665
C	3.00191	0.00118	−0.90574
C	2.13193	0.93199	−0.32211
C	2.49373	−1.18182	−1.45906
C	0.75377	0.67974	−0.29168
H	2.51995	1.83528	0.10028
C	1.11556	−1.43407	−1.42865
H	3.15798	−1.89248	−1.90476
C	0.24559	−0.50331	−0.84492
H	0.08951	1.39042	0.15396
H	0.72754	−2.33732	−1.8511
C	−1.26886	−0.78055	−0.81141
C	−3.34482	−0.7897	−0.173
N	−1.81817	−1.84606	−1.29559
N	−3.15393	−1.85195	−0.88481
O	−2.23155	0.1087	−0.23458
C	−4.61867	−0.52465	0.65077
H	−4.49563	−0.92331	1.63608
C	−5.82131	−1.20315	−0.03104
O	−5.65306	−2.25286	−0.70438
C	−7.22984	−0.59703	0.11115
C	−7.4172	0.57193	0.86105
C	−8.32424	−1.2144	−0.50937
C	−8.69896	1.12355	0.99039
H	−6.5816	1.0433	1.33486
C	−9.606	−0.66278	−0.38004
H	−8.18119	−2.10695	−1.08191
C	−9.79335	0.5062	0.36983
H	−8.84201	2.01608	1.56294
H	−10.4416	−1.13414	−0.85384
Br	−11.54028	1.25804	0.5461
H	−4.7912	0.5296	0.71155

Stoichiometry C26H23BrN2O6
Framework group C1[X(C26H23BrN2O6)]
Deg. of freedom 168
Full point group C1 NOp 1
Largest Abelian subgroup C1 NOp 1
Largest concise Abelian subgroup C1 NOp 1

Standard orientation:

Center Number	Atomic Number	Atomic Type	Coordinates (Angstroms) X	Y	Z
1	6	0	8.416391	1.390329	−0.481347
2	6	0	7.038189	1.138250	−0.451076
3	6	0	6.547707	0.003819	0.209526
4	6	0	7.435410	−0.878530	0.839878
5	6	0	8.813613	−0.626475	0.809601
6	6	0	9.304115	0.507945	0.148964
7	1	0	6.360394	1.811949	−0.932345

8	1	0	7.060898	−1.744682	1.344284
9	8	0	8.916853	2.547943	−1.155417
10	8	0	10.710459	0.765102	0.118014
11	8	0	9.719419	−1.526855	1.452827
12	6	0	9.070019	−2.138965	2.570199
13	1	0	8.210721	−2.679065	2.231377
14	1	0	9.747792	−2.812653	3.051519
15	1	0	8.765629	−1.383188	3.263776
16	6	0	11.422614	−0.474313	0.157860
17	1	0	11.175631	−0.998824	1.057185
18	1	0	11.150158	−1.069584	−0.688499
19	1	0	12.474921	−0.281926	0.134706
20	6	0	8.978477	3.643849	−0.238846
21	1	0	9.629826	3.394070	0.572481
22	1	0	9.352976	4.510025	−0.743218
23	1	0	7.998730	3.847467	0.140019
24	6	0	5.033212	−0.273219	0.242776
25	1	0	4.864657	−1.328884	0.288142
26	1	0	4.604020	0.193852	1.104483
27	8	0	4.425735	0.256136	−0.938601
28	6	0	3.019466	−0.001358	−0.907677
29	6	0	2.149212	0.930026	−0.325364
30	6	0	2.511585	−1.185114	−1.459669
31	6	0	0.771079	0.677589	−0.294923
32	1	0	2.536992	1.833890	0.096010
33	6	0	1.133453	−1.437546	−1.429237
34	1	0	3.176047	−1.896202	−1.904355
35	6	0	0.263210	−0.506216	−0.846828
36	1	0	0.106612	1.388703	0.149715
37	1	0	0.745667	−2.341376	−1.850676
38	6	0	−1.251206	−0.783660	−0.813298
39	6	0	−3.327293	−0.792368	−0.175323
40	7	0	−1.800233	−1.849846	−1.296300
41	7	0	−3.136087	−1.855449	−0.885799
42	8	0	−2.214161	0.106135	−0.237755
43	6	0	−4.601365	−0.526519	0.647858
44	1	0	−4.478471	−0.923967	1.633674
45	6	0	−5.803748	−1.206045	−0.033384
46	8	0	−5.635185	−2.256542	−0.705409
47	6	0	−7.212409	−0.599976	0.107771
48	6	0	−7.400112	0.569866	0.856209
49	6	0	−8.306580	−1.218278	−0.512230
50	6	0	−8.681986	1.121430	0.984605
51	1	0	−6.564689	1.041939	1.329622
52	6	0	−9.588454	−0.666705	−0.383841
53	1	0	−8.163266	−2.111499	−1.083649
54	6	0	−9.776152	0.503152	0.364573
55	1	0	−8.825309	2.014634	1.556043
56	1	0	−10.423880	−1.138774	−0.857243
57	35	0	−11.523239	1.254921	0.539554
58	1	0	−4.774071	0.527776	0.707317

Rotational constants (GHZ): 0.3685246 0.0154712 0.0152030
Standard basis: 6-311++G(d,p) (5D, 7F)
There are 957 symmetry adapted basis functions of A symmetry.
Integral buffers will be 131072 words long.
Raffenetti 2 integral format.
Two-electron integral symmetry is turned on.
957 basis functions, 1525 primitive gaussians, 994 cartesian basis functions
138 alpha electrons 138 beta electrons
nuclear repulsion energy 3481.0360156751 Hartrees.
NAtoms= 58 NActive= 58 NUniq= 58 SFac= 1.00D+00 NAtFMM= 60 NAOKFM=F Big=F

Polarizable Continuum Model (PCM)
==================================
Model : PCM.
Atomic radii : UFF (Universal Force Field).
Polarization charges : Total charges.
Charge compensation : None.
Solution method : Matrix inversion.
Cavity type : Scaled VdW (van der Waals Surface) (Alpha=1.100).
Cavity algorithm : GePol (No added spheres)
Default sphere list used, NSphG= 58.
Lebedev-Laikov grids with approx. 5.0 points / Ang**2.
Smoothing algorithm: Karplus/York (Gamma=1.0000).
Polarization charges: spherical gaussians, with
point-specific exponents (IZeta= 3).
Self-potential: point-specific (ISelfS= 7).
Self-field : sphere-specific E.n sum rule (ISelfD= 2).
Solvent : DiMethylSulfoxide, Eps= 46.826000 Eps(inf)= 2.007889

R16b.out

Title Card Required

Charge = 0 Multiplicity = 2
Symbolic Z-Matrix:

C	−3.79103	−2.04127	0.1557
C	−2.3898	−2.05614	0.14061
C	−1.67793	−0.85835	−0.00921
C	−2.36728	0.35431	−0.14392
C	−3.76851	0.36919	−0.12885
C	−4.4804	−0.82861	0.02093
H	−1.86346	−2.98203	0.24348
H	−1.82373	1.26885	−0.25828
O	−4.51741	−3.26353	0.30861
O	−5.91023	−0.81342	0.03624
O	−4.4719	1.60663	−0.26631
C	−3.68181	2.66704	0.27793
H	−2.75184	2.72508	−0.24808
H	−4.20813	3.59295	0.17511
H	−3.49427	2.47653	1.314
C	−6.38396	0.27211	−0.76507

H	−6.01321	1.19502	−0.3705
H	−6.0393	0.15007	−1.77066
H	−7.45384	0.2835	−0.75363
C	−4.74305	−3.51325	1.69844
H	−5.30816	−2.70755	2.11844
H	−5.28659	−4.42779	1.81285
H	−3.80324	−3.59127	2.20397
C	−0.13812	−0.87468	−0.02582
H	0.22056	−0.06992	−0.63295
H	0.23056	−0.76034	0.97213
O	0.31963	−2.11814	−0.56355
C	1.74946	−2.13307	−0.57909
C	2.45719	−2.61979	0.52821
C	2.44294	−1.66095	−1.70163
C	3.85843	−2.63427	0.51302
H	1.92769	−2.98035	1.38525
C	3.84417	−1.67544	−1.71682
H	1.90255	−1.28941	−2.54712
C	4.55191	−2.16206	−0.60948
H	4.39881	−3.00587	1.35849
H	4.37367	−1.31495	−2.57389
C	6.09173	−2.17788	−0.62613
C	8.15695	−2.11136	0.04293
N	6.80686	−1.75878	−1.6183
N	8.13573	−1.71598	−1.1878
O	6.89785	−2.65237	0.45797
C	9.39101	−2.00638	0.95816
H	9.3784	−1.06541	1.46739
C	10.67088	−2.11309	0.10838
O	10.67103	−1.70689	−1.08266
C	11.95054	−2.71693	0.71617
C	11.95036	−3.16924	2.04257
C	13.11521	−2.81413	−0.05714
C	13.11484	−3.7188	2.59564
H	11.06111	−3.09499	2.63303
C	14.27968	−3.3637	0.49593
H	13.11535	−2.46875	−1.06987
C	14.27949	−3.81603	1.82232
H	13.1147	−4.06416	3.60837
H	15.16893	−3.43794	−0.09452
Br	15.86657	−4.56507	2.57612

Stoichiometry C26H22BrN2O6(2)
Framework group C1[X(C26H22BrN2O6)]
Deg. of freedom 165
Full point group C1 NOp 1
Largest Abelian subgroup C1 NOp 1
Largest concise Abelian subgroup C1 NOp 1

Standard orientation:

Center Number	Atomic Number	Atomic Type	Coordinates (Angstroms) X	Y	Z

1	6	0	8.399153	1.391479	−0.478860
2	6	0	7.020917	1.139585	−0.448604
3	6	0	6.530392	0.004430	0.210721
4	6	0	7.418087	−0.878828	0.839812
5	6	0	8.796324	−0.626958	0.809549
6	6	0	9.286869	0.508186	0.150189
7	1	0	6.343129	1.813978	−0.928910
8	1	0	7.043542	−1.745532	1.343243
9	8	0	8.899659	2.549832	−1.151628
10	8	0	10.693247	0.765155	0.119254
11	8	0	9.702121	−1.528265	1.451488
12	6	0	9.052859	−2.141629	2.568252
13	1	0	8.193402	−2.681178	2.228955
14	1	0	9.730625	−2.816011	3.048609
15	1	0	8.748737	−1.386647	3.262812
16	6	0	11.405211	−0.474422	0.157441
17	1	0	11.158334	−0.999987	1.056180
18	1	0	11.132480	−1.068620	−0.689583
19	1	0	12.457544	−0.282177	0.134298
20	6	0	8.961654	3.644612	−0.233738
21	1	0	9.613134	3.393742	0.577147
22	1	0	9.336185	4.511341	−0.737135
23	1	0	7.982020	3.847927	0.145582
24	6	0	5.015860	−0.272405	0.243955
25	1	0	4.847144	−1.328097	0.288073
26	1	0	4.586925	0.193687	1.106321
27	8	0	4.408218	0.258485	−0.936648
28	6	0	3.001914	0.001179	−0.905740
29	6	0	2.131934	0.931994	−0.322110
30	6	0	2.493726	−1.181823	−1.459063
31	6	0	0.753766	0.679742	−0.291684
32	1	0	2.519948	1.835283	0.100281
33	6	0	1.115560	−1.434070	−1.428646
34	1	0	3.157979	−1.892476	−1.904755
35	6	0	0.245590	−0.503309	−0.844921
36	1	0	0.089508	1.390422	0.153959
37	1	0	0.727539	−2.337324	−1.851102
38	6	0	−1.268864	−0.780550	−0.811407
39	6	0	−3.344817	−0.789700	−0.173004
40	7	0	−1.818165	−1.846060	−1.295590
41	7	0	−3.153932	−1.851947	−0.884813
42	8	0	−2.231553	0.108699	−0.234579
43	6	0	−4.618672	−0.524648	0.650770
44	1	0	−4.495639	−0.923293	1.636084
45	6	0	−5.821308	−1.203152	−0.031043
46	8	0	−5.653057	−2.252857	−0.704381
47	6	0	−7.229842	−0.597028	0.111147
48	6	0	−7.417198	0.571933	0.861047
49	6	0	−8.324244	−1.214400	−0.509374
50	6	0	−8.698956	1.123546	0.990385
51	1	0	−6.581599	1.043295	1.334857
52	6	0	−9.606002	−0.662777	−0.380042

53	1	0	−8.181194	−2.106948	−1.081909
54	6	0	−9.793353	0.506199	0.369834
55	1	0	−8.842014	2.016078	1.562940
56	1	0	−10.441604	−1.134136	−0.853841
57	35	0	−11.540282	1.258035	0.546099

Rotational constants (GHZ): 0.3687222 0.0154809 0.0152123
Standard basis: 6-311++G(d,p) (5D, 7F)
There are 950 symmetry adapted basis functions of A symmetry.
Integral buffers will be 131072 words long.
Raffenetti 2 integral format.
Two-electron integral symmetry is turned on.
950 basis functions, 1516 primitive gaussians, 987 cartesian basis functions
138 alpha electrons 137 beta electrons
nuclear repulsion energy 3453.1760965192 Hartrees.
NAtoms= 57 NActive= 57 NUniq= 57 SFac= 1.00D+00 NAtFMM= 60 NAOKFM=F Big=F

Polarizable Continuum Model (PCM)
=====================================
Model : PCM.
Atomic radii : UFF (Universal Force Field).
Polarization charges : Total charges.
Charge compensation : None.
Solution method : Matrix inversion.
Cavity type : Scaled VdW (van der Waals Surface) (Alpha=1.100).
Cavity algorithm : GePol (No added spheres)
Default sphere list used, NSphG= 57.
Lebedev-Laikov grids with approx. 5.0 points / Ang**2.
Smoothing algorithm: Karplus/York (Gamma=1.0000).
Polarization charges: spherical gaussians, with
point-specific exponents (IZeta= 3).
Self-potential: point-specific (ISelfS= 7).
Self-field : sphere-specific E.n sum rule (ISelfD= 2).
Solvent : DiMethylSulfoxide, Eps= 46.826000 Eps(inf)= 2.007889
----

r26b.out

Title Card Required

Charge = 0 Multiplicity = 2
Symbolic Z-Matrix:

C	−3.23876	−2.19773	0.62514
C	−1.89106	−1.89804	0.38402
C	−1.53	−0.63336	−0.09987
C	−2.51733	0.33186	−0.34288
C	−3.86515	0.03213	−0.10072
C	−4.2255	−1.23281	0.38334
H	−1.13781	−2.63493	0.56917
H	−2.24218	1.29763	−0.71318
O	−3.6087	−3.48817	1.1179
O	−5.60018	−1.53951	0.63098
O	−4.87326	1.01646	−0.34707

C	−4.32644	2.32196	−0.1429
H	−3.50768	2.47451	−0.81454
H	−5.08097	3.05813	−0.32762
H	−3.98162	2.41018	0.86605
C	−6.426	−0.79597	−0.26916
H	−6.26309	0.25107	−0.12067
H	−6.17896	−1.056	−1.2773
H	−7.45408	−1.02696	−0.08297
C	−3.6152	−3.46943	2.54759
H	−4.31874	−2.74027	2.8916
H	−3.89345	−4.43478	2.91533
H	−2.63857	−3.21926	2.90574
C	−0.0491	−0.30278	−0.36516
H	0.55127	0.12335	0.4112
O	0.52327	−0.56476	−1.64942
C	1.92049	−0.8345	−1.50446
C	2.40469	−1.41773	−0.32566
C	2.80611	−0.51445	−2.54255
C	3.77465	−1.67996	−0.18476
H	1.72893	−1.66278	0.46692
C	4.17558	−0.77657	−2.40166
H	2.43667	−0.06982	−3.44285
C	4.6601	−1.35832	−1.22239
H	4.1443	−2.12574	0.71506
H	4.85119	−0.5322	−3.19462
C	6.16572	−1.64339	−1.06622
C	8.12393	−1.9186	−0.16768
N	7.04837	−1.37622	−1.97232
N	8.30857	−1.55289	−1.39395
O	6.75197	−2.22815	0.10192
C	9.24686	−2.00405	0.88352
H	9.33651	−1.06346	1.38557
C	10.5794	−2.34346	0.1891
O	10.78501	−1.96985	−0.99472
C	11.6605	−3.14021	0.94439
C	11.43077	−3.55676	2.26258
C	12.87369	−3.44866	0.31385
C	12.41295	−4.28305	2.94975
H	10.50543	−3.32026	2.74448
C	13.85604	−4.17514	1.00072
H	13.04996	−3.12975	−0.6921
C	13.62546	−4.59246	2.31861
H	12.23696	−4.60145	3.95619
H	14.78224	−4.41108	0.51907
Br	14.96356	−5.58288	3.2547
H	9.01152	−2.76761	1.59515

Stoichiometry C26H22BrN2O6(2)
Framework group C1[X(C26H22BrN2O6)]
Deg. of freedom 165
Full point group C1 NOp 1
Largest Abelian subgroup C1 NOp 1
Largest concise Abelian subgroup C1 NOp 1

Standard orientation:

Center Number	Atomic Number	Atomic Type	Coordinates (Angstroms) X	Y	Z
1	6	0	7.670011	1.879897	−0.630456
2	6	0	6.446584	1.224597	−0.435351
3	6	0	6.407425	0.002701	0.249754
4	6	0	7.592424	−0.564073	0.739698
5	6	0	8.815846	0.092063	0.545225
6	6	0	8.854251	1.314097	−0.139899
7	1	0	5.542443	1.657123	−0.809687
8	1	0	7.563164	−1.497640	1.262328
9	8	0	7.711557	3.126263	−1.330280
10	8	0	10.101777	1.984426	−0.338599
11	8	0	10.025355	−0.484551	1.045713
12	6	0	9.740088	−1.248786	2.220273
13	1	0	9.047091	−2.027502	1.979269
14	1	0	10.645274	−1.680266	2.594220
15	1	0	9.314597	−0.610595	2.966145
16	6	0	11.154568	1.020424	−0.424641
17	1	0	11.195543	0.454807	0.482718
18	1	0	10.968917	0.361954	−1.247410
19	1	0	12.087111	1.523479	−0.573840
20	6	0	7.568375	4.201714	−0.398982
21	1	0	8.365809	4.165113	0.313582
22	1	0	7.601019	5.133746	−0.923210
23	1	0	6.631428	4.111184	0.109547
24	6	0	5.063379	−0.718403	0.464888
25	1	0	4.483766	−0.519117	1.341870
26	8	0	4.590500	−1.654859	−0.507227
27	6	0	3.160355	−1.677596	−0.493492
28	6	0	2.442947	−0.527520	−0.137345
29	6	0	2.476093	−2.851809	−0.836097
30	6	0	1.041314	−0.552134	−0.122959
31	1	0	2.964851	0.369321	0.123748
32	6	0	1.074950	−2.876387	−0.821683
33	1	0	3.023480	−3.729749	−1.108684
34	6	0	0.357481	−1.726939	−0.464078
35	1	0	0.493524	0.326106	0.148464
36	1	0	0.553093	−3.773076	−1.083546
37	6	0	−1.182457	−1.754905	−0.445040
38	6	0	−3.240235	−1.321963	0.098832
39	7	0	−1.888866	−2.795759	−0.743467
40	7	0	−3.213077	−2.517326	−0.393059
41	8	0	−1.998292	−0.633798	−0.087687
42	6	0	−4.462866	−0.711237	0.809805
43	1	0	−4.410451	−0.927301	1.856394
44	6	0	−5.754385	−1.314683	0.225911
45	8	0	−5.750542	−2.487147	−0.230828
46	6	0	−7.050894	−0.482511	0.201509
47	6	0	−7.054515	0.823526	0.709606

48	6	0	−8.226801	−1.030979	−0.328581
49	6	0	−8.233005	1.581804	0.686208
50	1	0	−6.157470	1.241835	1.115480
51	6	0	−9.405467	−0.272846	−0.352301
52	1	0	−8.224751	−2.028491	−0.715457
53	6	0	−9.408385	1.033620	0.154942
54	1	0	−8.235271	2.579310	1.073813
55	1	0	−10.303265	−0.691491	−0.757506
56	35	0	−11.014122	2.067184	0.122297
57	1	0	−4.467349	0.348709	0.663832

Rotational constants (GHZ): 0.2506246 0.0166910 0.0158778
Standard basis: 6-311++G(d,p) (5D, 7F)
There are 950 symmetry adapted basis functions of A symmetry.
Integral buffers will be 131072 words long.
Raffenetti 2 integral format.
Two-electron integral symmetry is turned on.
950 basis functions, 1516 primitive gaussians, 987 cartesian basis functions
138 alpha electrons 137 beta electrons
nuclear repulsion energy 3462.7881226289 Hartrees.
NAtoms = 57 NActive = 57 NUniq = 57 SFac = 1.00D+00 NAtFMM = 60 NAOKFM=F Big=F

Polarizable Continuum Model (PCM)
=================================
Model : PCM.
Atomic radii : UFF (Universal Force Field).
Polarization charges: Total charges.
Charge compensation: None.
Solution method : Matrix inversion.
Cavity type : Scaled VdW (van der Waals Surface) (Alpha = 1.100).
Cavity algorithm : GePol (No added spheres)
Default sphere list used, NSphG = 57.
Lebedev-Laikov grids with approx. 5.0 points / Ang**2.
Smoothing algorithm: Karplus/York (Gamma = 1.0000).
Polarization charges: spherical gaussians, with
point-specific exponents (IZeta = 3).
Self-potential: point-specific (ISelfS = 7).
Self-field: sphere-specific E.n sum rule (ISelfD = 2).
Solvent : DiMethylSulfoxide, Eps = 46.826000 Eps(inf) = 2.007889
